# Mammalian ranges are experiencing erosion of natural darkness

**DOI:** 10.1038/srep12042

**Published:** 2015-07-09

**Authors:** James P. Duffy, Jonathan Bennie, América P. Durán, Kevin J. Gaston

**Affiliations:** 1 Environment and Sustainability Institute, University of Exeter, Penryn, Cornwall TR10 9FE, UK

## Abstract

The continuous increase in the intensity and extent of anthropogenic artificial light has significantly shaped Earth’s nighttime environment. This environmental change has effects across the natural world, with consequences for organismal physiology and behaviour and the abundances and distributions of species. Here, we evaluate for the first time the relations between the spatio-temporal patterns of anthropogenic nighttime light and the distribution of terrestrial mammals, one of the most endangered species groups and one that expresses varying time partitioning strategies. Using descriptive statistics, trend tests and spatial prioritization analysis we show that in most places on earth there is a terrestrial mammal species whose range is experiencing detectable artificial light. For most species this tends only to be for small parts of their range, and those affected across large parts are typically rare. Over time (1992–2012), an increase in mean light intensity was found for the ranges of the majority of species, with very few experiencing a decrease. Moreover, nocturnal species are more likely to experience an increase in light within their ranges. This is of conservation concern as many terrestrial mammals are nocturnal and therefore often particularly vulnerable to a pressure such as artificial light at night.

Natural light regimes are being disrupted over an increasingly large extent of the Earth’s surface. This has resulted in part from the direct effects of artificial light at night (ALAN), predominantly produced by electric lighting. However, it is also caused by the skyglow that results from the diffuse scattering of these light emissions in the atmosphere. Indeed, by 2001, 19% of the global land surface was already estimated to be above a threshold of light set for polluted status^1^.

ALAN has a wide diversity of biological impacts, affecting both diurnal and nocturnal species^2^,^3^,^4^,^5^. These can be characterised as comprising influences on natural light regimes as a resource and as a source of information to organisms^6^. The former include effects on photosynthesis, the partitioning of activity between day and night, and dark repair and recovery, and the latter effects on circadian clocks and photoperiodism, visual perception, and spatial orientation (for review of empirical examples see^6^). This array of influences has given rise to substantial concerns, and growing evidence, about the consequences for the abundance and distribution of species, community structure, and ecosystem processes and dynamics^7^,^8^,^9^,^10^, and as to the ways in which these can be mitigated^11^,^12^.

Despite these concerns, there are a lack of estimates regarding how the occurrence of, and trends in, ALAN are distributed with respect to the geographic ranges of the species in particular taxonomic groups. Key questions concern how many species have ranges that are experiencing ALAN, what proportions of their ranges are influenced and how this is changing, and in which regions those species which are most extensively influenced reside.

Here we address these issues using terrestrial mammals as a case study, investigating the extent and change in ALAN within their ranges. These provide an interesting study group because they are globally distributed, occupy a broad range of environments, and exhibit a wide diversity of time partitioning behaviour that can in substantial part be predicted based on patterns of natural light and darkness^13^. Moreover, ALAN has already been shown to have a wide diversity of impacts on mammals including on their circadian rhythms and photoperiodism^14^, immune responses^15^, foraging^16^, movements^17^,^18^,^19^, and reproduction^20^.

## Results

**Figure 1:**
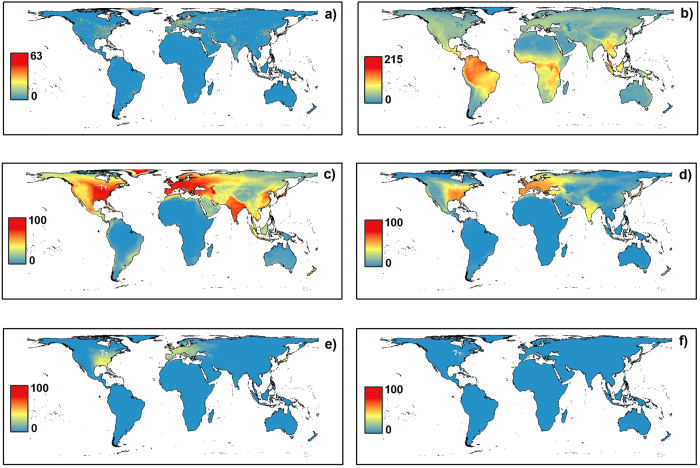
The global distributions of (**a**) nighttime lights, shown using a 2012 DMSP-OLS nighttime lights image (downloaded from http://ngdc.noaa.gov/eog/dmsp/downloadV4composites.html); (**b**) mammalian species richness; the numbers of species with given percentages of their geographic range experiencing detectable ALAN (DN > 5.5) for (**c**) >10%; (**d**) >20%; (**e**) >40% and (**f**) >60%. Richness maps created using range maps from the IUCN^30^ in R (R Core Team. R: A language and environment for statistical computing. R Foundation for Statistical Computing, Vienna, Austria) using the ‘raster’ package (Hijmans, R.J. & van Etten, J. raster: Geographic data analysis and modeling. Version 2.1–49). Final display made in ArcGIS (ESRI. ArcGIS Desktop: Version 10, Environmental System Research Institute, Redlands, CA).

**Figure 2:**
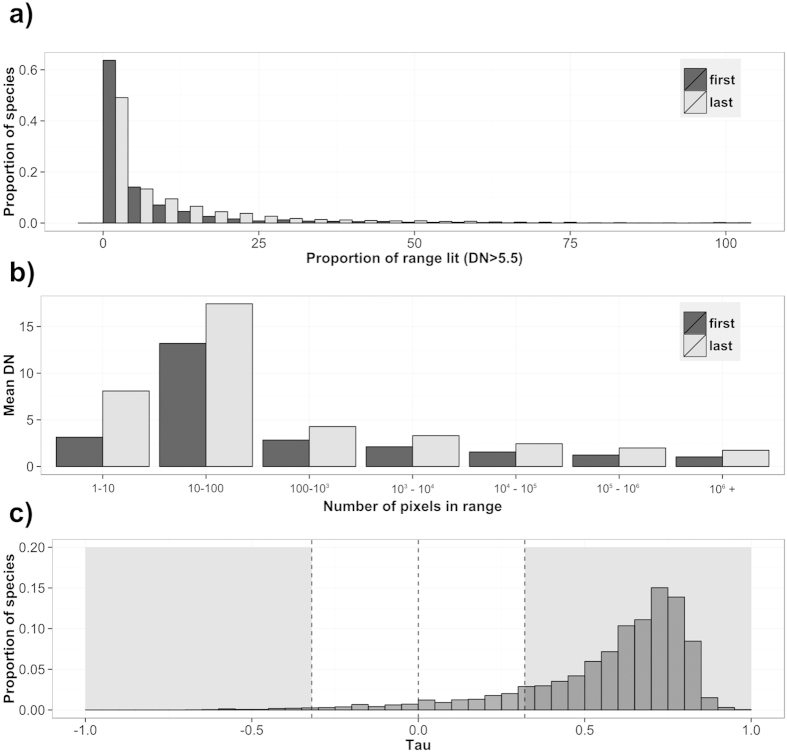
The magnitude and trend of conflict between ALAN and mammals. (**a**) The difference in the spread of light between the first and last four years for all species, (**b**) A comparison of the average intensity of light between the first and last four years for affected species (those that have a mean DN higher than 0), (**c**) The strength of trend in ALAN over the 21 year study period by tau value (Mann-Kendall trend test). Grey shaded areas indicate significantly negative and positive results (p < 0.05) respectively, for affected species.

In very few places was there a terrestrial mammal species whose geographic range was not experiencing some detectable ALAN (Fig. 1(c)). Examples of these areas include small pockets of land found in Madagascar, central Australia, Baja California in Mexico, the Amazon rainforest, parts of large islands and several small islands in south-east Asia. For many species lighting occurred in only a small proportion of their range, with 3594 experiencing ALAN in less than 10% of their range during 1992–1995 (Fig. 2a). As the proportion of the geographic range experiencing light increased, the geographical focus shifted mainly to the Northern Hemisphere, specifically, N. America, Europe, and Japan (Fig. 1(d–e)). Few species were experiencing ALAN in more than 60% of their range, with those with ranges bound to small islands predominantly falling into this group (Fig. 1f). Indeed, those species experiencing ALAN over high proportions of their geographic ranges were typically rare (small range sizes), with the more widely distributed species almost invariably occurring in many places with no ALAN (Fig. 2b).

Of the 4370 mammal species studied, many have seen change in the ALAN within their range between 1992–2012. The majority (n = 3624) experienced a significant increase in the mean nighttime light (Mann-Kendall trend test, Τ > 0, p < 0.05) (Fig. 2c). Nocturnal species were most prominent in this group (62.4% of species), whereas 18.4% were diurnal, 7% cathemeral, 2.5% crepuscular and the remainder lacked information on time partitioning strategy. Forty-one species experienced significant decreases in ALAN within their ranges (Mann-Kendall trend test, Τ < 0, p < 0.05) (Fig. 2c). Nineteen of these species were nocturnal, 9 diurnal, 5 cathemeral, 1 crepuscular and 7 lacked information. Nocturnal species were significantly more likely to have experienced an increase in ALAN, and less likely to experience no change than species with other time partitioning behaviours (χ-squared=84.45, p < 0.001).

We found no significant difference between species in different Red List categories with regards to the strength of the trend (for significantly positive trending species) in ALAN, the change in the proportion of lit pixels (see methods section for description), or the change in mean DN values (see Supplementary Information) within their range over time.

For two periods, three spatial prioritization analyses were performed with one representing mammals (Only Mammals), one representing ALAN (Only Light), and a third representing mammals and, when possible, avoiding ALAN areas (Balanced) (see Methods section for more details). Using the resulting priority values from these analyses, we used Spearman rank coefficients to assess the extent of conflict between different priority sites. In the period 1992–1995, the correlation between *Only Mammals* and *Only Light* priority areas was low (0.22, p < 0.01), indicating that a low relative proportion of mammals’ ranges experienced ALAN. The strength of this correlation slightly increased (0.29, p < 0.01) for the period 2009–2012. However, the decrease in the correlation between *Only Mammals* and *Balanced* priority areas from the period 1992–1995 (0.80, p < 0.01) to 2009–2012 (0.73, p < 0.01) suggests that avoiding ALAN within areas that over-represent the occurrence of mammals became more difficult with time. This is also reflected in the increase over time of the correlation between *Only Light* and *Balanced* (−0.03, p < 0.01 [1992–1995]; −0.06, p < 0.01 [2009–2012]), showing that balancing a set of areas that represent mammals and ALAN without overlapping has tended to become harder.

## Discussion

Much concern has been expressed with regards to the potential impacts of ALAN on mammals, and many studies have documented significant effects, particularly on foraging and movement patterns^17^,^20^,^21^,^22^,^23^. Whilst the effects of ALAN can be highly species specific and critically influenced by sensory (e.g. visual acuity) and environmental (e.g. habitat cover) variables^24^, mammals are especially vulnerable because a high proportion of species in the group are nocturnal^13^. Here we show that, in addition, the majority of species experience ALAN in some portion of their geographic range, that in most cases this ALAN is increasing, and that for some rare species this can be occurring over most of their range (Fig 2b). These increases could have both positive and negative effects on mammals. ALAN can effectively increase the length of available activity time for diurnal species, reduce it for nocturnal species and cause more complex changes to the activity cycles of crepuscular and cathemeral species. While some species may be able to utilise the additional light for foraging or other behaviours, others may suffer from increased predation risk^25^, or altered patterns of time partitioning through competition for resources^26^,^27^.

As often with other global studies of anthropogenic impacts, these results are only indicative of what might be occurring. First, albeit being the best that are freely available, the species range data are relatively coarse in resolution compared to the ALAN data, and therefore the levels of overlap may be somewhat over or under estimated. However, equally, the ALAN data do not capture the full extent of skyglow, which may propagate emissions even hundreds of kilometres from the source^28^, suggesting that the overlap between species ranges and ALAN is underestimated. Second, ALAN co-occurs with, and is arguably an important component of, urbanisation, and thus rarely acts as an anthropogenic pressure in isolation. Moreover, it is likely to act in additive and synergistic ways with an array of such pressures, including habitat fragmentation, climate change and chemical pollution^29^.

Given their high conservation profile and the predominance of nocturnal species, mammals are of particular concern with regard to the impacts of ALAN. However, there is little reason to believe that the high proportion of species shown here with geographic ranges experiencing ALAN, and the growth in this effect, are atypical of many other groups of organisms.

## Material and Methods

### Data Sources

Analyses were based on global mammal species range maps downloaded from IUCN (International Union for the Conservation of Nature)^30^. The terrestrial and non-fossorial (following^13^) subset of species was used (including bats), excluding *Melomys rubicola*, which has a range too small for analysis. All data were projected to a Behrmann equal-area projection using ArcGIS (ESRI. ArcGIS Desktop: Version 10, Environmental System Research Institute, Redlands, CA). Species were classified as (i) diurnal, nocturnal, crepuscular, cathemeral or unknown, following^13^; and (ii) according to their IUCN threat category^30^, excluding those listed as ‘Extinct in the Wild’ or ‘Extinct’, leaving 4370 species. Species classed as ‘Data Deficient’ by the IUCN lack spatial information and were therefore not included in this analysis.

Nighttime stable lights composite images were downloaded from the National Oceanic and Atmospheric Administration archives (1992–2012, n = 21), created with data from the Defense Meteorological Satellite Program’s Operational Linescan System (DMSP/OLS). The images are at 1 km resolution, and each pixel is represented by a digital number (DN) between zero and 63. Zero represents darkness, while brightly lit areas saturate at values of 63. Images were intercalibrated and drift-corrected according to^31^.

### Extraction & Analysis

The DMSP data for all years were extracted for each species using GDAL utility tools (GDAL: Geospatial Data Abstraction Library: Version 1.10, Open Source Geospatial Foundation). Pixels were associated with a mammalian range if their centre point fell within the polygon boundary. This extraction resulted in a raster file per species per year that could then be further analysed in R (R Core Team. R: A language and environment for statistical computing. R Foundation for Statistical Computing, Vienna, Austria), using the ‘raster’ (Hijmans, R.J. & van Etten, J. raster: Geographic data analysis and modeling. Version 2.1–49) package. Using the package ‘Kendall’ (McLeod, A.I. Kendall: Kendall rank correlation and Mann-Kendall trend test. Version 2.2), Mann-Kendall trend tests identified significant trends in yearly-mean nighttime light values for each species. Chi-squared tests were also performed in R to explore the relationship between time-partitioning behaviour and changes in ALAN within species ranges.

It has been shown that over 94% of observed increases in DN of more than 3 units and over 93% of observed decreases of the same magnitude could be attributed to a known change on the ground consistent with the direction of change (i.e. growth in urban areas, deindustrialisation)^31^. We defined a threshold of darkness of <5.5 DN. Lit pixels were those with any value above this threshold. By using a threshold effectively twice the detection limit for change, we defined a conservative estimate of lit area and limited the extent to which dark sites may be classified as lit due to noise in the data or calibration errors.

### Zonation analysis

The spatial prioritization software Zonation^32^ was used to investigate spatial conflicts between mammal occurrence and ALAN, and their changes over time. Data were aggregated to 10 km. Due to the non-additive nature of the DMSP data, the images containing average DN values for the periods 1992–1995 and 2009–2012 were converted to binary, with a 1 representing a positive DN and a 0 for 0 DN. For these periods we generated three hierarchical prioritization maps, in which the representation of mammals and ALAN on the Earth’s surface was optimized according to the following rules: i) *Only Mammals*, spatial prioritization aiming to optimize the representation of mammals (i.e. relative proportion of mammals’ ranges). Light raster was weighted as 0 (hence ignored in the prioritization selection) and each of the species as 1; ii) *Only Light*, spatial prioritization aiming to optimize the representation of lit areas. Here the light raster was weighted as 1 and each of the mammal species weighted as 0; and iii) *Balanced*, spatial prioritization aiming to optimize the representation of mammals while simultaneously excluding lit areas, thus reducing potential conservation conflicts. Light raster was weighted as −1 (hence ALAN is avoided within mammal priority areas) and all the species were weighted equally ω = 1/4370. This implies that species were jointly equal to the ALAN value 1.

## Additional Information

**How to cite this article**: Duffy, J. P. *et al*. Mammalian ranges are experiencing erosion of natural darkness. *Sci. Rep*. **5**, 12042; doi: 10.1038/srep12042 (2015).

## Supplementary Material

Supplementary Information
