# Supplementary material for: Mammalian ranges are experiencing erosion of natural darkness
Source: Sci Rep. 2015 Jul 9;5:12042. doi: 10.1038/srep12042 (PMC4496780; doi:10.1038/srep12042)
Supplement: Supplementary Information [file srep12042-s1.pdf]

Supplementary Information

Mammalian ranges are experiencing erosion of natural darkness

James P. Duffy[1]*, Jonathan Bennie[1], América P. Durán[1] and Kevin J. Gaston[1]

1 -Environment and Sustainability Institute, University of Exeter, Penryn, Cornwall TR10 9FE, UK

**Corresponding author – james.philip.duffy@gmail.com*

Figure S1: **The change in mean ALAN values within species ranges between the first (1992-1995) and last (2009-2012) four years, grouped by Red List Status.**

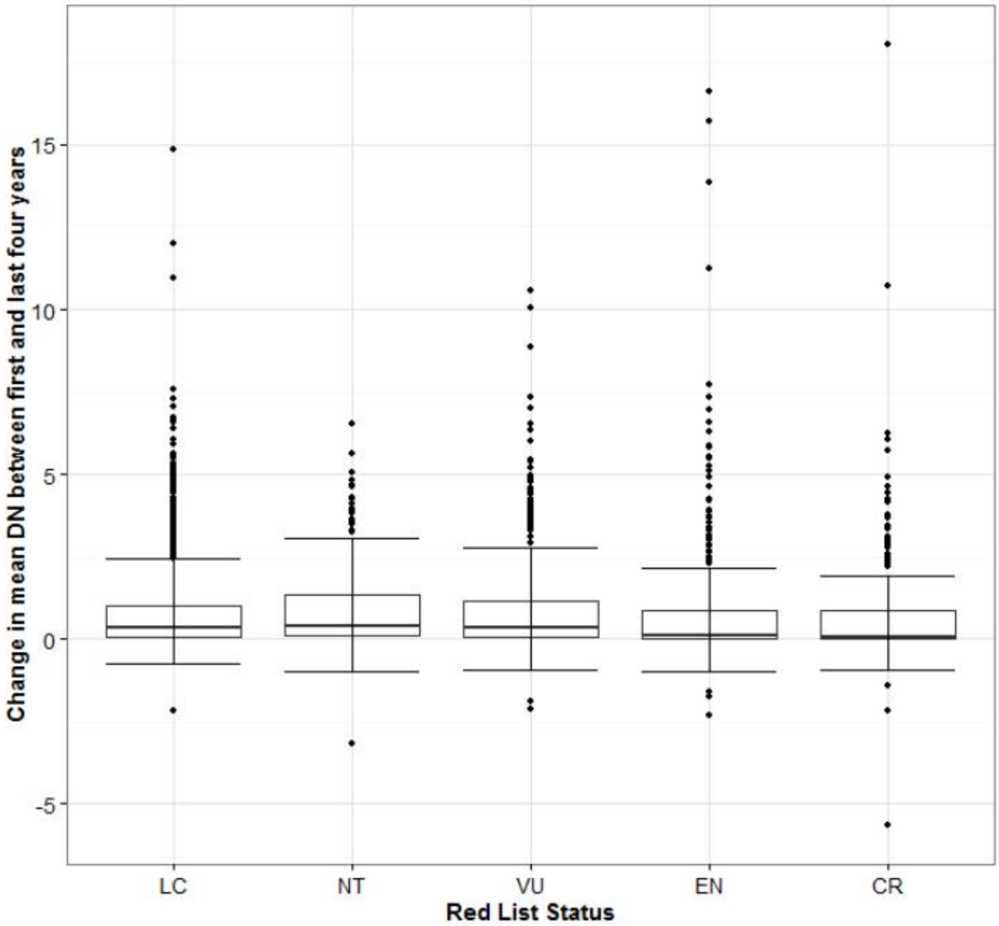

Figure S2: Output maps from spatial prioritization analysis for each scenario: Only mammals (a), Only light (1992-1995) (b), Only light (2009-2012) (c),

Balanced (1992-1995) (d) and Balanced (2009-2012) (e). Values between 0 and 1 indicate the rank of the cell in the removal process with those close to 0 removed first (lower priority) and those close to 1 (higher priority) removed last. Figure created using ArcMap 10.1 (ESRI, 2012).

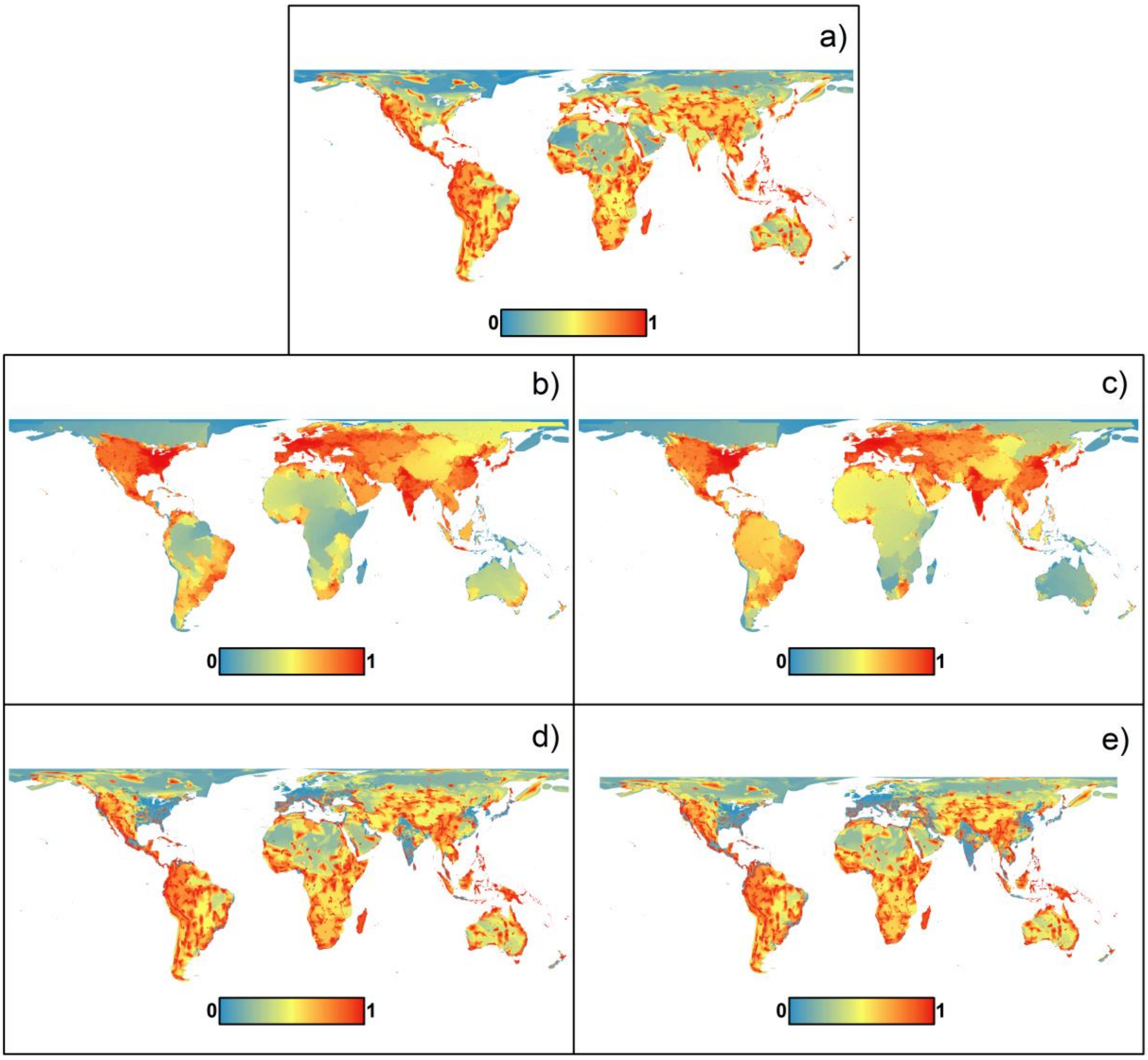

Table S1: The values of ALAN and Mann-Kendall trend analysis results for all species.

Species	Average DN (1992-1995)	Average DN (2008-2012)	Tau	p-value
Abeomelomys_sevia	0.09	0.13	0.15	0.35
Abrawayaomys_ruschii	0.07	0.3	0.74	0.00
Abrocoma_bennettii	1.56	3.31	0.84	0.00
Abrocoma_boliviensis	0.86	1.83	0.25	0.12
Abrocoma_cinerea	0.1	0.29	0.77	0.00
Abrothrix_andinus	0.2	0.44	0.78	0.00
Abrothrix_hershkovitzi	0	0	-0.12	0.55

Abrothrix_illuteus	0.64	1.83	0.79	0.00
Abrothrix_jelskii	0.18	0.47	0.72	0.00
Abrothrix_lanosus	0.18	0.33	0.51	0.00
Abrothrix_longipilis	0.38	0.85	0.76	0.00
Abrothrix_markhami	0	0	-0.12	0.55
Abrothrix_olivaceus	0.33	0.72	0.75	0.00
Abrothrix_sanborni	0.02	0.13	0.75	0.00
Acerodon_celebensis	0.21	0.61	0.59	0.00
Acerodon_humilis	0.01	0.06	0.35	0.03
Acerodon_jubatus	0.75	1.71	0.62	0.00
Acerodon_leucotis	0.05	0.24	0.58	0.00
Acerodon_mackloti	0.21	0.61	0.59	0.00
Acinonyx_jubatus	0.06	0.11	0.66	0.00
Acomys_airensis	0	0.01	0.59	0.00
Acomys_cahirinus	0.45	0.83	0.73	0.00
Acomys_cineraceus	0.1	0.24	0.75	0.00
Acomys_dimidiatus	1.87	3.8	0.86	0.00
Acomys_ignitus	0.3	0.53	0.67	0.00
Acomys_johannis	0.06	0.16	0.66	0.00
Acomys_kempi	0.01	0.01	0.45	0.00
Acomys_louisae	0.01	0.02	0.57	0.00
Acomys_mullah	0.03	0.08	0.67	0.00
Acomys_percivali	0.02	0.05	0.49	0.00
Acomys_russatus	1.61	3.5	0.79	0.00
Acomys_seurati	0.02	0.05	0.67	0.00
Acomys_spinosissimus	0.16	0.37	0.70	0.00
Acomys_subspinosus	1.88	2.52	0.60	0.00
Acomys_wilsoni	0.05	0.11	0.61	0.00
Aconaemys_fuscus	0.18	0.53	0.71	0.00
Acrobates_pygmaeus	0.62	0.87	0.65	0.00
Addax_nasomaculatus	0.05	0.11	0.71	0.00
Aegialomys_galapagoensis	0.37	0.79	0.47	0.00
Aegialomys_xanthaeolus	1.11	2.5	0.77	0.00
Aepeomys_lugens	1.8	2.34	0.58	0.00
Aepeomys_reigi	2.8	4.34	0.55	0.00
Aepyceros_melampus	0.18	0.38	0.74	0.00
Aepyprymnus_rufescens	0.45	0.72	0.66	0.00
Aeretes_melanopterus	0.1	0.59	0.78	0.00
Aethalops_aequalis	0.13	0.16	0.12	0.45
Aethalops_alecto	1.63	3.77	0.80	0.00
Aethomys_bocagei	0.16	0.58	0.79	0.00
Aethomys_chrysophilus	0.08	0.14	0.60	0.00
Aethomys_granti	0.06	0.09	0.49	0.00
Aethomys_hindei	0.04	0.08	0.65	0.00
Aethomys_ineptus	2.24	3.62	0.76	0.00
Aethomys_kaiseri	0.04	0.08	0.67	0.00

Aethomys_namaquensis	0.44	0.71	0.69	0.00
Aethomys_nyikae	0.06	0.14	0.70	0.00
Aethomys_thomasi	0.01	0.13	0.68	0.00
Ailuropoda_melanoleuca	0.01	0.09	0.55	0.00
Ailurops_melanotis	0.02	0.15	0.25	0.12
Ailurops_ursinus	0.21	0.61	0.61	0.00
Ailurus_fulgens	0.05	0.36	0.82	0.00
Akodon_aerosus	0.06	0.25	0.68	0.00
Akodon_affinis	2.28	2.95	0.43	0.01
Akodon_albiventer	0.11	0.29	0.76	0.00
Akodon_azarae	0.94	1.76	0.75	0.00
Akodon_bogotensis	2.88	3.81	0.53	0.00
Akodon_boliviensis	0.27	0.68	0.79	0.00
Akodon_budini	0.79	1.56	0.68	0.00
Akodon_cursor	2.59	4.77	0.74	0.00
Akodon_dayi	0.37	0.52	0.48	0.00
Akodon_dolores	1.41	2.95	0.77	0.00
Akodon_fumeus	0.29	0.52	0.70	0.00
Akodon_iniscatus	0.21	0.41	0.61	0.00
Akodon_juninensis	0.22	0.58	0.70	0.00
Akodon_kofordi	0.01	0.08	0.52	0.00
Akodon_latebricola	0.15	0.98	0.74	0.00
Akodon_lutescens	0.67	1.57	0.81	0.00
Akodon_mimus	0.55	1.17	0.75	0.00
Akodon_molinae	0.26	0.63	0.76	0.00
Akodon_mollis	0.42	1.38	0.76	0.00
Akodon_montensis	2.17	4.33	0.81	0.00
Akodon_orophilus	0.08	0.22	0.62	0.00
Akodon_paranaensis	1.43	3.42	0.82	0.00
Akodon_reigi	0.22	0.4	0.52	0.00
Akodon_serrensis	2.41	4.41	0.77	0.00
Akodon_siberiae	0.5	1.17	0.78	0.00
Akodon_simulator	0.63	1.58	0.77	0.00
Akodon_spegazzinii	0.66	1.57	0.79	0.00
Akodon_subfuscus	0.34	0.75	0.71	0.00
Akodon_surdus	0.08	0.25	0.47	0.00
Akodon_sylvanus	0.05	0.14	0.75	0.00
Akodon_toba	0.09	0.24	0.76	0.00
Akodon_torques	0.15	0.39	0.62	0.00
Alcelaphus_buselaphus	0.18	0.31	0.71	0.00
Alces_alces	1.97	2.73	0.43	0.01
Alces_americanus	0.6	0.63	0.09	0.61
Alionycteris_paucidentata	0.1	0.25	0.41	0.01
Allactaga_balikunica	0.02	0.09	0.76	0.00
Allactaga_bullata	0.08	0.28	0.80	0.00
Allactaga_elater	0.71	1.17	0.70	0.00

Allactaga_euphratica	1.51	2.45	0.68	0.00
Allactaga_hotsoni	0.15	0.41	0.69	0.00
Allactaga_major	1.69	1.86	0.01	0.98
Allactaga_severtzovi	0.76	0.83	0.35	0.03
Allactaga_sibirica	0.23	0.62	0.79	0.00
Allactaga_tetradactyla	2.95	6.56	0.87	0.00
Allactaga_vinogradovi	1.6	1.93	0.31	0.05
Allactaga_williamsi	1.68	3.51	0.74	0.00
Allactodipus_bobrinskii	0.25	0.55	0.55	0.00
Allenopithecus_nigroviridis	0	0	0.63	0.00
Allocricetulus_curtatus	0.07	0.26	0.76	0.00
Allocricetulus_eversmanni	0.74	0.96	0.39	0.01
Alopex_lagopus	0.13	0.19	0.52	0.00
Alouatta_arctoidea	1.98	3.39	0.71	0.00
Alouatta_belzebul	0.49	0.91	0.66	0.00
Alouatta_caraya	0.35	0.78	0.77	0.00
Alouatta_discolor	0.03	0.09	0.70	0.00
Alouatta_guariba	2.06	4.17	0.82	0.00
Alouatta_juara	0.52	0.78	0.52	0.00
Alouatta_macconnelli	0.11	0.23	0.76	0.00
Alouatta_nigerrima	0.02	0.06	0.73	0.00
Alouatta_palliata	1.2	2.46	0.74	0.00
Alouatta_pigra	0.46	1.06	0.59	0.00
Alouatta_puruensis	0.04	0.15	0.75	0.00
Alouatta_sara	0.22	0.24	-0.06	0.74
Alouatta_seniculus	0.52	0.78	0.52	0.00
Alouatta_ululata	0.49	1.43	0.76	0.00
Alticola_argentatus	0.56	0.85	0.66	0.00
Alticola_barakshin	0	0.01	0.47	0.00
Alticola_lemminus	0.04	0.03	-0.21	0.19
Alticola_macrotis	0.01	0.02	0.45	0.00
Alticola_montosa	0.17	0.51	0.70	0.00
Alticola_roylei	0.06	0.59	0.76	0.00
Alticola_semicanus	0.03	0.08	0.62	0.00
Alticola_stoliczkanus	0.02	0.09	0.82	0.00
Alticola_strelzowi	0.13	0.21	0.37	0.02
Alticola_tuvinicus	0.18	0.19	-0.10	0.53
Ametrida_centurio	0.36	0.62	0.70	0.00
Ammodorcas_clarkei	0	0	0.16	0.32
Ammospermophilus_harrisii	1.86	2.59	0.69	0.00
Ammospermophilus_interpres	0.91	1.24	0.63	0.00
Ammospermophilus_leucurus	1.25	1.5	0.55	0.00
Ammospermophilus_nelsoni	5.05	5.71	0.22	0.17
Ammotragus_lervia	0.07	0.22	0.75	0.00
Amorphochilus_schnablii	1.48	3.03	0.79	0.00
Anathana_ellioti	2.32	5.68	0.79	0.00

Andalgalomys_olrogi	0.09	0.37	0.77	0.00
Andalgalomys_pearsoni	0	0.03	0.70	0.00
Andinomys_edax	0.18	0.49	0.79	0.00
Anisomys_imitator	0.03	0.07	0.45	0.00
Anomalurus_beecrofti	0.41	0.49	0.30	0.07
Anomalurus_derbianus	0.25	0.34	0.45	0.00
Anomalurus_pusillus	0.05	0.11	0.69	0.00
Anotomys_leander	5.98	10.57	0.86	0.00
Anoura_caudifer	0.62	1.18	0.73	0.00
Anoura_cultrata	1.63	2.43	0.65	0.00
Anoura_geoffroyi	1	1.85	0.72	0.00
Anoura_latidens	1.46	2.05	0.59	0.00
Anoura_luismanueli	2.87	3.81	0.56	0.00
Anourosorex_assamensis	0.65	1.29	0.59	0.00
Anourosorex_squamipes	0.25	1.31	0.80	0.00
Anourosorex_yamashinai	13.96	19.85	0.82	0.00
Antechinomys_laniger	0.02	0.02	0.15	0.35
Antechinus_adustus	0.04	0.15	0.36	0.02
Antechinus_agilis	1.18	1.61	0.67	0.00
Antechinus_bellus	0.02	0.04	0.35	0.03
Antechinus_flavipes	0.49	0.71	0.57	0.00
Antechinus_godmani	0.05	0.21	0.35	0.03
Antechinus_leo	0	0.01	0.26	0.11
Antechinus_minimus	0.37	0.51	0.38	0.02
Antechinus_stuartii	2.16	2.56	0.50	0.00
Antechinus_subtropicus	5.94	7.94	0.69	0.00
Antechinus_swainsonii	1.16	1.47	0.56	0.00
Antidorcas_marsupialis	0.42	0.59	0.67	0.00
Antilocapra_americana	1.27	1.61	0.34	0.03
Antilope_cervicapra	2.43	5.34	0.68	0.00
Antrozous_pallidus	2.05	2.58	0.58	0.00
Aonyx_capensis	0.26	0.4	0.65	0.00
Aonyx_cinerea	0.97	2.62	0.81	0.00
Aonyx_congicus	0.05	0.11	0.69	0.00
Aotus_azarae	0.12	0.29	0.75	0.00
Aotus_brumbacki	0.33	0.87	0.48	0.00
Aotus_griseimembra	1.75	2.64	0.41	0.01
Aotus_lemurinus	1.98	2.84	0.51	0.00
Aotus_miconax	0.08	0.25	0.67	0.00
Aotus_nancymaae	0.01	0.06	0.70	0.00
Aotus_nigriceps	0.03	0.12	0.78	0.00
Aotus_trivirgatus	0.06	0.13	0.70	0.00
Aotus_vociferans	0.09	0.16	0.67	0.00
Aplodontia_rufa	3.91	3.86	0.02	0.93
Apodemus_agrarius	2.43	4.15	0.74	0.00
Apodemus_alpicola	4.37	8.12	0.73	0.00

Apodemus_argenteus	12.67	14.27	0.52	0.00
Apodemus_chevrieri	0.28	1.41	0.80	0.00
Apodemus_draco	0.56	2.07	0.81	0.00
Apodemus_epimelas	3.19	6.26	0.71	0.00
Apodemus_flavicollis	5.24	7.78	0.67	0.00
Apodemus_gurkha	0	0	0.03	0.92
Apodemus_hyrcanicus	4.65	7.69	0.86	0.00
Apodemus_latronum	0.06	0.51	0.81	0.00
Apodemus_mystacinus	2.16	4.45	0.75	0.00
Apodemus_pallipes	0.06	0.24	0.74	0.00
Apodemus_peninsulae	0.88	1.92	0.81	0.00
Apodemus_ponticus	2.04	3.02	0.50	0.00
Apodemus_rusiges	0.47	0.83	0.58	0.00
Apodemus_semotus	14.06	19.99	0.82	0.00
Apodemus_speciosus	12.61	14.19	0.52	0.00
Apodemus_sylvaticus	6.53	9.91	0.84	0.00
Apodemus_uralensis	2.1	2.75	0.35	0.03
Apodemus_witherbyi	2.19	3.89	0.70	0.00
Apomys_camiguinensis	0	0	-0.19	0.32
Apomys_datae	0.28	0.74	0.60	0.00
Apomys_hylocetes	0.02	0.17	0.37	0.02
Apomys_insignis	0.38	0.96	0.46	0.00
Apomys_microdon	0.34	0.8	0.54	0.00
Apomys_musculus	0.23	0.62	0.44	0.01
Aproteles_bulmerae	0	0	-0.12	0.55
Arabitragus_jayakari	2.18	7.71	0.92	0.00
Arborimus_albipes	3.25	3.21	0.02	0.93
Arborimus_longicaudus	3.65	3.6	0.05	0.79
Arborimus_pomo	1.43	1.13	-0.45	0.00
Archboldomys_luzonensis	0	0.09	0.06	0.76
Arctictis_binturong	0.58	1.65	0.78	0.00
Arctocebus_aureus	0.1	0.18	0.56	0.00
Arctocebus_calabarensis	2.82	2.49	-0.20	0.22
Arctogalidia_trivirgata	0.46	1.26	0.77	0.00
Arctonyx_collaris	0.96	3.02	0.85	0.00
Ardops_nichollsi	10.95	18	0.87	0.00
Arielulus_aureocollaris	0.01	0.28	0.53	0.00
Arielulus_circumdatus	0.95	2.46	0.78	0.00
Arielulus_societatis	1.82	3.3	0.57	0.00
Arielulus_torquatus	13.29	18.89	0.82	0.00
Ariteus_flavescens	7.39	9.9	0.50	0.00
Artibeus_amplus	1	1.55	0.62	0.00
Artibeus_anderseni	0.07	0.16	0.76	0.00
Artibeus_aztecus	2.54	4.13	0.70	0.00
Artibeus_cinereus	0.11	0.28	0.77	0.00
Artibeus_concolor	0.21	0.45	0.73	0.00

Artibeus_fimbriatus	1.93	3.9	0.81	0.00
Artibeus_fraterculus	0.96	2.15	0.77	0.00
Artibeus_glaucus	0.55	0.91	0.65	0.00
Artibeus_gnomus	0.29	0.62	0.77	0.00
Artibeus_hirsutus	1.44	2.12	0.50	0.00
Artibeus_incomitatus	0	0	-0.12	0.55
Artibeus_jamaicensis	1.62	2.9	0.66	0.00
Artibeus_lituratus	0.64	1.23	0.73	0.00
Artibeus_obscurus	0.39	0.82	0.75	0.00
Artibeus_phaeotis	0.94	1.62	0.63	0.00
Artibeus_planirostris	0.45	0.93	0.74	0.00
Artibeus_toltecus	1.69	3.02	0.66	0.00
Artibeus_watsoni	0.94	1.77	0.70	0.00
Arvicanthis_abyssinicus	0.06	0.22	0.75	0.00
Arvicanthis_ansorgei	0.05	0.15	0.75	0.00
Arvicanthis_blicki	0.02	0.05	0.55	0.00
Arvicanthis_nairobae	0.27	0.61	0.63	0.00
Arvicanthis_neumanni	0.02	0.04	0.54	0.00
Arvicanthis_niloticus	0.14	0.3	0.79	0.00
Arvicanthis_rufinus	0.39	0.68	0.67	0.00
Arvicola_amphibius	2.71	3.78	0.66	0.00
Arvicola_sapidus	6.67	10.65	0.88	0.00
Arvicola_scherman	7.02	11.04	0.76	0.00
Asellia_patrizii	0.08	0.24	0.72	0.00
Asellia_tridens	0.56	1.04	0.72	0.00
Aselliscus_stoliczkanus	0.36	1.44	0.81	0.00
Aselliscus_tricuspidatus	0.05	0.11	0.33	0.04
Atelerix_albiventris	0.07	0.16	0.70	0.00
Atelerix_algirus	2.81	5.63	0.83	0.00
Atelerix_frontalis	0.76	1.14	0.71	0.00
Atelerix_sclateri	0.01	0.02	0.40	0.01
Ateles_belzebuth	0.06	0.15	0.51	0.00
Ateles_chamek	0.05	0.11	0.72	0.00
Ateles_fusciceps	0.79	1.37	0.53	0.00
Ateles_geoffroyi	1.32	2.59	0.66	0.00
Ateles_hybridus	1.33	1.98	0.45	0.00
Ateles_marginatus	0.03	0.09	0.68	0.00
Ateles_paniscus	0.04	0.1	0.70	0.00
Atelocynus_microtis	0.05	0.13	0.77	0.00
Atherurus_africanus	0.39	0.49	0.36	0.02
Atherurus_macrourus	0.69	2.38	0.84	0.00
Atilax_paludinosus	0.23	0.36	0.68	0.00
Atlantoxerus_getulus	0.27	0.82	0.76	0.00
Auliscomys_boliviensis	0.31	0.74	0.77	0.00
Auliscomys_pictus	0.19	0.52	0.69	0.00
Auliscomys_sublimis	0.14	0.36	0.78	0.00

Avahi_cleesei	0	0	-0.12	0.55
Avahi_laniger	0	0.01	0.51	0.00
Avahi_occidentalis	0	0	-0.16	0.42
Axis_axis	2.28	4.92	0.70	0.00
Axis_calamianensis	0	0.1	0.59	0.00
Axis_kuhlii	0.01	0.12	0.04	0.83
Axis_porcinus	1.35	2.47	0.68	0.00
Babyrousa_babyrussa	0.02	0.03	0.01	0.98
Babyrousa_celebensis	0.07	0.27	0.57	0.00
Babyrousa_togeanensis	0	0	-0.12	0.55
Baiomys_musculus	2.36	4.02	0.67	0.00
Baiomys_taylori	3.52	4.81	0.66	0.00
Baiyankamys_shawmayeri	0.08	0.13	0.26	0.11
Balantiopteryx_infusca	5.45	7.91	0.59	0.00
Balantiopteryx_io	0.95	1.88	0.60	0.00
Balantiopteryx_plicata	2.7	4.4	0.69	0.00
Balionycteris_maculata	0.81	2.4	0.84	0.00
Bandicota_bengalensis	2.18	4.51	0.76	0.00
Bandicota_indica	1.81	4.09	0.79	0.00
Bandicota_savilei	0.75	2.55	0.79	0.00
Barbastella_barbastellus	6.8	10.29	0.84	0.00
Barbastella_leucomelas	0.92	1.94	0.78	0.00
Bassaricyon_alleni	0.22	0.36	0.67	0.00
Bassaricyon_beddardi	0	0.01	0.57	0.00
Bassaricyon_gabbii	0.88	1.54	0.60	0.00
Bassariscus_astutus	2.62	3.38	0.58	0.00
Bassariscus_sumichrasti	1.44	2.75	0.65	0.00
Batomys_granti	0.35	0.86	0.71	0.00
Batomys_russatus	0.01	0.1	0.36	0.03
Batomys_salomonseni	0.34	1.06	0.49	0.00
Bauerus_dubiaquercus	1.08	2.01	0.63	0.00
Bdeogale_crassicauda	0.07	0.16	0.64	0.00
Bdeogale_jacksoni	0.32	0.77	0.52	0.00
Bdeogale_nigripes	0.06	0.13	0.70	0.00
Bdeogale_omnivora	0.34	0.58	0.52	0.00
Beamys_hindei	0.16	0.3	0.72	0.00
Beatragus_hunteri	0	0	0.03	0.88
Berylmys_berdmorei	0.42	1.87	0.77	0.00
Berylmys_bowersi	0.85	2.64	0.83	0.00
Bettongia_gaimardi	0.81	1.05	0.38	0.02
Bettongia_lesueur	3.34	2.33	-0.42	0.01
Bettongia_penicillata	0	0	-0.05	0.82
Bettongia_tropica	0	0.02	0.18	0.29
Bibimys_chacoensis	0.93	2.22	0.76	0.00
Bibimys_labiosus	2.68	5.27	0.79	0.00
Bibimys_torresi	1.3	2.89	0.55	0.00

Bison_bison	0.4	0.4	0.01	0.98
Bison_bonasus	0.72	3.61	0.50	0.00
Biswamoyopterus_biswasi	0.1	0.3	0.36	0.02
Blanfordimys_afghanus	0.3	0.64	0.68	0.00
Blanfordimys_bucharensis	1.76	1.34	-0.08	0.65
Blarina_brevicauda	6.64	7.29	0.23	0.16
Blarina_carolinensis	7.5	9.3	0.54	0.00
Blarina_hylophaga	4.57	5.56	0.42	0.01
Blarinella_griselda	0.41	1.68	0.80	0.00
Blarinella_quadraticauda	0.08	0.84	0.76	0.00
Blarinella_wardi	0.17	1.34	0.85	0.00
Blastocerus_dichotomus	0.44	0.99	0.81	0.00
Bos_gaurus	0.57	1.48	0.72	0.00
Bos_javanicus	0.2	0.75	0.78	0.00
Bos_mutus	0	0	0.12	0.49
Bos_sauveli	0	0.06	0.56	0.00
Boselaphus_tragocamelus	2.42	5.13	0.70	0.00
Brachiones_przewalskii	0.07	0.29	0.76	0.00
Brachylagus_idahoensis	1.3	1.39	0.17	0.29
Brachyphylla_cavernarum	23.48	27.52	0.65	0.00
Brachyphylla_nana	1.5	3.08	0.57	0.00
Brachytarsomys_albicauda	0	0.02	0.52	0.00
Brachytarsomys_villosa	0	0	-0.12	0.55
Brachyteles_arachnoides	8.01	11.73	0.77	0.00
Brachyteles_hypoxanthus	1.63	4.05	0.72	0.00
Brachyuromys_betsileoensis	0.01	0.03	0.50	0.00
Brachyuromys_ramirohitra	0.01	0.02	0.52	0.00
Bradypus_pygmaeus	0	0	-0.12	0.55
Bradypus_torquatus	2.7	5.35	0.79	0.00
Bradypus_tridactylus	0.1	0.2	0.72	0.00
Bradypus_variegatus	0.54	1.05	0.72	0.00
Brucepattersonius_iheringi	1.39	3.1	0.80	0.00
Bubalus_arnee	0.14	0.45	0.63	0.00
Bubalus_depressicornis	0.06	0.23	0.60	0.00
Bubalus_mindorensis	0	0.04	0.24	0.17
Bubalus_quarlesi	0.05	0.16	0.65	0.00
Budorcas_taxicolor	0.18	0.98	0.82	0.00
Bullimus_bagobus	0.34	0.9	0.46	0.00
Bullimus_gamay	0.16	0.39	0.26	0.11
Bullimus_luzonicus	0.2	0.52	0.47	0.00
Bunolagus_monticularis	0.04	0.07	0.64	0.00
Bunomys_andrewsi	0.22	0.62	0.65	0.00
Bunomys_chrysocomus	0.21	0.63	0.61	0.00
Bunomys_coelestis	0	0	-0.12	0.55
Bunomys_fratrorum	0.59	1.71	0.50	0.00
Bunomys_penitus	0.01	0.01	0.19	0.24

Bunomys_prolatus	0	0	-0.12	0.55
Burramys_parvus	0.4	0.72	0.13	0.41
Cabassous_chacoensis	0.07	0.19	0.74	0.00
Cabassous_tatouay	1.25	2.59	0.83	0.00
Cabassous_unicinctus	0.25	0.54	0.78	0.00
Cacajao_ayresi	0	0	-0.12	0.55
Cacajao_calvus	0	0.02	0.68	0.00
Cacajao_hosomi	0	0.01	0.50	0.00
Cacajao_melanocephalus	0	0.03	0.68	0.00
Caenolestes_caniventer	1.31	3.46	0.85	0.00
Caenolestes_condorensis	0	0	-0.12	0.55
Caenolestes_convelatus	0.86	1.79	0.72	0.00
Caenolestes_fuliginosus	2.4	3.96	0.71	0.00
Callibella_humilis	0	0	0.51	0.00
Callicebus_aureipalatii	0	0.01	0.60	0.00
Callicebus_baptista	0.07	0.21	0.81	0.00
Callicebus_barbarabrownae	0.56	1.43	0.73	0.00
Callicebus_bernhardi	0.03	0.1	0.70	0.00
Callicebus_brunneus	0.08	0.3	0.78	0.00
Callicebus_caligatus	0.01	0.05	0.70	0.00
Callicebus_caquetensis	0.01	0.07	0.28	0.08
Callicebus_cinerascens	0.01	0.05	0.71	0.00
Callicebus_coimbrai	2.66	5.83	0.79	0.00
Callicebus_cupreus	0.01	0.04	0.53	0.00
Callicebus_discolor	0.31	0.47	0.63	0.00
Callicebus_donacophilus	0.15	0.05	-0.59	0.00
Callicebus_dubius	0.06	0.23	0.77	0.00
Callicebus_hoffmannsi	0.02	0.07	0.76	0.00
Callicebus_lucifer	0.01	0.01	0.66	0.00
Callicebus_lugens	0.05	0.09	0.66	0.00
Callicebus_medemi	0.11	0.18	0.42	0.01
Callicebus_melanochir	0.73	1.65	0.76	0.00
Callicebus_modestus	0.01	0.04	0.28	0.09
Callicebus_moloch	0.04	0.14	0.74	0.00
Callicebus_nigrifrons	3.12	5.56	0.75	0.00
Callicebus_oenanthe	0.35	1.22	0.69	0.00
Callicebus_olallae	0	0	-0.12	0.55
Callicebus_ornatus	0.33	1.49	0.72	0.00
Callicebus_pallescens	0.02	0.06	0.72	0.00
Callicebus_personatus	0.93	2.6	0.70	0.00
Callicebus_purinus	0.03	0.06	0.22	0.17
Callicebus_regulus	0	0.02	0.67	0.00
Callicebus_torquatus	0.01	0.06	0.70	0.00
Callimico_goeldii	0.03	0.08	0.73	0.00
Callistomys_pictus	2.13	3.34	0.67	0.00
Callithrix_aurita	5.76	9.26	0.78	0.00

Callithrix_flaviceps	0.65	2.63	0.67	0.00
Callithrix_geoffroyi	0.41	1.46	0.78	0.00
Callithrix_jacchus	0.87	1.75	0.71	0.00
Callithrix_kuhlii	0.63	1.19	0.70	0.00
Callithrix_penicillata	0.61	1.38	0.76	0.00
Callosciurus_adamsi	0.66	1.52	0.71	0.00
Callosciurus_baluensis	0.1	0.14	0.30	0.07
Callosciurus_caniceps	1.63	4.27	0.80	0.00
Callosciurus_erythraeus	0.93	2.88	0.85	0.00
Callosciurus_finlaysonii	1.05	2.74	0.71	0.00
Callosciurus_inornatus	0.26	1.07	0.74	0.00
Callosciurus_melanogaster	0	0.01	-0.06	0.76
Callosciurus_nigrovittatus	1.93	4.46	0.79	0.00
Callosciurus_notatus	1.11	2.55	0.77	0.00
Callosciurus_orestes	0.03	0.04	0.23	0.16
Callosciurus_phayrei	0.12	0.66	0.80	0.00
Callosciurus_prevostii	0.66	1.69	0.75	0.00
Callosciurus_pygerythrus	0.36	0.89	0.72	0.00
Callosciurus_quinquestriatus	0.15	1.29	0.84	0.00
Calomys_boliviae	0.53	1.15	0.77	0.00
Calomys_callidus	0.38	1.04	0.71	0.00
Calomys_callosus	0.3	0.7	0.74	0.00
Calomys_expulsus	0.42	1.03	0.72	0.00
Calomys_fecundus	0.08	0.23	0.70	0.00
Calomys_hummelincki	1.26	2.54	0.72	0.00
Calomys_laucha	0.78	1.51	0.76	0.00
Calomys_lepidus	0.16	0.41	0.74	0.00
Calomys_musculinus	0.48	0.96	0.78	0.00
Calomys_sorellus	0.17	0.52	0.68	0.00
Calomys_tener	0.77	1.73	0.76	0.00
Calomys_tocantinsi	0.04	0.11	0.72	0.00
Calomys_venustus	1.38	2.81	0.73	0.00
Calomyscus_bailwardi	3.19	6.11	0.79	0.00
Calomyscus_baluchi	0.24	0.35	0.42	0.01
Calomyscus_elburzensis	0.99	2.05	0.67	0.00
Calomyscus_hotsoni	0.23	0.7	0.70	0.00
Calomyscus_mystax	2.61	5.15	0.85	0.00
Calomyscus_urartensis	1.45	3.25	0.65	0.00
Caluromys_derbianus	1.39	2.65	0.71	0.00
Caluromys_lanatus	0.49	0.99	0.79	0.00
Caluromys_philander	0.68	1.34	0.75	0.00
Caluromysiops_irrupta	0.04	0.2	0.77	0.00
Camelus_ferus	0	0.02	0.43	0.01
Canis_adustus	0.07	0.15	0.71	0.00
Canis_aureus	0.86	1.71	0.74	0.00
Canis_latrans	3	3.51	0.45	0.00

Canis_lupus	0.94	1.49	0.73	0.00
Canis_mesomelas	0.24	0.4	0.70	0.00
Canis_rufus	0.33	0.51	0.25	0.12
Canis_simensis	0	0	0.25	0.18
Cannomys_badius	0.4	1.29	0.72	0.00
Cansumys_canus	0.42	1.45	0.75	0.00
Capra_aegagrus	1.53	3.24	0.80	0.00
Capra_caucasica	0.19	0.73	0.54	0.00
Capra_cylindricornis	0.04	0.18	0.58	0.00
Capra_falconeri	1.05	1	0.10	0.53
Capra_ibex	2.52	4.73	0.67	0.00
Capra_nubiana	0.58	1.51	0.80	0.00
Capra_pyrenaica	2.6	5.02	0.78	0.00
Capra_sibirica	0.05	0.16	0.75	0.00
Capra_walie	0	0	-0.12	0.55
Capreolus_capreolus	5.13	7.64	0.74	0.00
Capreolus_pygargus	0.9	1.51	0.71	0.00
Capricornis_crispus	8.36	10	0.46	0.00
Capricornis_milneedwardsii	0.39	1.51	0.81	0.00
Capricornis_rubidus	0.01	0.08	0.74	0.00
Capricornis_sumatraensis	0.2	0.77	0.65	0.00
Capricornis_swinhoei	0.99	2.13	0.51	0.00
Capricornis_thar	0.18	0.58	0.75	0.00
Caprolagus_hispidus	0.51	1.34	0.70	0.00
Capromys_pilorides	0.89	2.45	0.59	0.00
Caracal_aurata	0.09	0.21	0.73	0.00
Caracal_caracal	0.62	1.2	0.75	0.00
Cardioderma_cor	0.04	0.09	0.65	0.00
Carollia_brevicauda	0.43	0.82	0.70	0.00
Carollia_castanea	0.56	0.98	0.64	0.00
Carollia_manu	0.06	0.19	0.51	0.00
Carollia_perspicillata	0.57	1.13	0.73	0.00
Carollia_sowelli	1.34	2.62	0.66	0.00
Carollia_subrufa	1.9	4.24	0.72	0.00
Carpomys_phaeurus	0.03	0.14	0.36	0.02
Caryomys_eva	0.1	0.63	0.76	0.00
Caryomys_inez	1.51	3.51	0.81	0.00
Casinycteris_argynnis	0	0.02	0.64	0.00
Castor_canadensis	2.97	3.36	0.35	0.03
Castor_fiber	3.27	4.8	0.50	0.00
Catagonus_wagneri	0.02	0.05	0.55	0.00
Cavia_aperea	0.88	1.68	0.75	0.00
Cavia_fulgida	2.95	5.23	0.73	0.00
Cavia_intermedia	0	0	-0.12	0.55
Cavia_magna	3.32	6.83	0.85	0.00
Cavia_tschudii	0.23	0.62	0.74	0.00

Cebuella_pygmaea	0.06	0.12	0.68	0.00
Cebus_albifrons	0.23	0.4	0.64	0.00
Cebus_apella	0.09	0.22	0.73	0.00
Cebus_capucinus	0.79	1.6	0.63	0.00
Cebus_cay	0.31	0.73	0.80	0.00
Cebus_flavius	6.24	9.22	0.60	0.00
Cebus_kaapori	0.35	0.92	0.74	0.00
Cebus_libidinosus	0.33	0.79	0.75	0.00
Cebus_macrocephalus	0.04	0.12	0.66	0.00
Cebus_nigritus	2.48	5.02	0.81	0.00
Cebus_olivaceus	0.48	0.84	0.71	0.00
Cebus_robustus	0.36	1.29	0.78	0.00
Cebus_xanthosternos	0.71	1.59	0.74	0.00
Centronycteris_centralis	0.91	1.52	0.64	0.00
Centronycteris_maximiliani	0.25	0.56	0.73	0.00
Centurio_senex	1.69	2.85	0.64	0.00
Cephalophus_adersi	0.52	1.42	0.68	0.00
Cephalophus_callipygus	0.09	0.2	0.67	0.00
Cephalophus_dorsalis	0.08	0.2	0.73	0.00
Cephalophus_harveyi	0.15	0.29	0.61	0.00
Cephalophus_jentinki	0.02	0.13	0.71	0.00
Cephalophus_leucogaster	0.05	0.13	0.74	0.00
Cephalophus_natalensis	0.2	0.41	0.71	0.00
Cephalophus_niger	0.82	1.2	0.48	0.00
Cephalophus_nigrifrons	0.14	0.18	0.30	0.06
Cephalophus_ogilbyi	1.16	1.18	0.00	1.00
Cephalophus_rufilatus	0.08	0.17	0.69	0.00
Cephalophus_silvicultor	0.23	0.3	0.40	0.01
Cephalophus_spadix	0.03	0.02	-0.08	0.65
Cephalophus_weynsi	0.01	0.02	0.48	0.00
Cephalophus_zebra	0.02	0.14	0.69	0.00
Ceratotherium_simum	0.18	0.31	0.70	0.00
Cercartetus_caudatus	0.09	0.17	0.43	0.01
Cercartetus_concinnus	0.36	0.48	0.52	0.00
Cercartetus_lepidus	0.26	0.35	0.38	0.02
Cercartetus_nanus	1.12	1.44	0.57	0.00
Cercocebus_agilis	0.02	0.03	0.58	0.00
Cercocebus_atys	0.14	0.39	0.58	0.00
Cercocebus_galeritus	0	0	-0.12	0.55
Cercocebus_sanjei	0	0	-0.22	0.20
Cercocebus_torquatus	3.78	3.43	-0.30	0.07
Cercopithecus_ascanius	0.02	0.05	0.66	0.00
Cercopithecus_campbelli	0.24	0.63	0.70	0.00
Cercopithecus_cephus	0.09	0.16	0.57	0.00
Cercopithecus_diana	0.22	0.66	0.65	0.00
Cercopithecus_dryas	0	0	-0.12	0.55

Cercopithecus_erythrogaster	7.95	7.02	-0.35	0.03
Cercopithecus_erythrotis	0.26	0.49	0.68	0.00
Cercopithecus_hamlyni	0.01	0.03	0.47	0.00
Cercopithecus_lhoesti	0	0	0.46	0.00
Cercopithecus_mitis	0.13	0.29	0.74	0.00
Cercopithecus_mona	1.81	1.78	-0.10	0.53
Cercopithecus_neglectus	0.03	0.07	0.67	0.00
Cercopithecus_nictitans	0.62	0.64	0.01	0.98
Cercopithecus_petaurista	0.26	0.69	0.69	0.00
Cercopithecus_pogonias	0.04	0.1	0.70	0.00
Cercopithecus_preussi	0.08	0.12	0.02	0.93
Cercopithecus_sclateri	10.92	8.81	-0.39	0.01
Cercopithecus_solatus	0.01	0.05	0.64	0.00
Cerdocyon_thous	0.79	1.58	0.77	0.00
Cerradomys_maracajuensis	0.3	0.69	0.74	0.00
Cerradomys_scotti	0.25	0.57	0.74	0.00
Cerradomys_subflavus	1.12	2.37	0.77	0.00
Cervus_elaphus	2.15	3.49	0.77	0.00
Cervus_nippon	9.06	10.57	0.49	0.00
Chacodelphys_formosa	0.26	0.84	0.76	0.00
Chaetodipus_arenarius	0.21	0.38	0.65	0.00
Chaetodipus_artus	1.33	2.14	0.53	0.00
Chaetodipus_baileyi	1.94	2.72	0.69	0.00
Chaetodipus_californicus	11.59	11.89	0.21	0.19
Chaetodipus_dalquesti	0.99	2.7	0.77	0.00
Chaetodipus_eremicus	0.68	1.05	0.61	0.00
Chaetodipus_fallax	11.35	12.58	0.58	0.00
Chaetodipus_formosus	1.9	2.2	0.46	0.00
Chaetodipus_goldmani	1.25	1.89	0.46	0.00
Chaetodipus_hispidus	2.55	3.3	0.54	0.00
Chaetodipus_intermedius	1.57	2.19	0.68	0.00
Chaetodipus_nelsoni	0.78	1.27	0.58	0.00
Chaetodipus_penicillatus	2.45	3.19	0.64	0.00
Chaetodipus_pernix	1.48	2.26	0.49	0.00
Chaetodipus_rudinoris	0.77	1.14	0.63	0.00
Chaetodipus_spinatus	1.22	1.7	0.63	0.00
Chaetomys_subspinosus	1.86	4.17	0.80	0.00
Chaetophractus_nationi	0.15	0.38	0.78	0.00
Chaetophractus_vellerosus	0.28	0.69	0.80	0.00
Chaetophractus_villosus	0.45	0.9	0.76	0.00
Chalinolobus_dwyeri	0.87	1.16	0.62	0.00
Chalinolobus_gouldii	0.15	0.22	0.56	0.00
Chalinolobus_morio	0.44	0.6	0.59	0.00
Chalinolobus_neocaledonicus	0.42	0.98	0.70	0.00
Chalinolobus_nigrogriseus	0.18	0.27	0.65	0.00
Chalinolobus_picatus	0.05	0.1	0.53	0.00

Chalinolobus_tuberculatus	0.81	1.18	0.52	0.00
Cheirogaleus_major	0	0	0.09	0.61
Cheirogaleus_medius	0	0.01	0.29	0.07
Cheiromeles_parvidens	0.65	1.37	0.65	0.00
Cheiromeles_torquatus	1.1	2.49	0.76	0.00
Chelemys_macronyx	0.22	0.6	0.75	0.00
Chelemys_megalonyx	1.63	3.73	0.81	0.00
Chilomys_instans	2.18	3.56	0.73	0.00
Chilonatalus_micropus	1.5	3.09	0.57	0.00
Chilonatalus_tumidifrons	0.34	0.62	0.45	0.00
Chimarrogale_hantu	1.41	3.48	0.69	0.00
Chimarrogale_himalayica	1.18	3.6	0.83	0.00
Chimarrogale_phaeura	0.27	0.22	-0.09	0.61
Chimarrogale_platycephalus	10.37	11.99	0.55	0.00
Chimarrogale_styani	0.16	0.83	0.82	0.00
Chinchilla_chinchilla	0	0	0.19	0.28
Chinchilla_lanigera	0.2	1.38	0.70	0.00
Chinchillula_sahamae	0.17	0.47	0.73	0.00
Chionomys_gud	0.4	0.7	0.47	0.00
Chionomys_nivalis	2.8	5.1	0.82	0.00
Chionomys_roberti	0.79	1.42	0.50	0.00
Chiroderma_doriae	1.2	2.44	0.78	0.00
Chiroderma_improvisum	13.83	20.18	0.75	0.00
Chiroderma_salvini	1.06	1.78	0.62	0.00
Chiroderma_trinitatum	0.28	0.5	0.70	0.00
Chiroderma_villosum	0.37	0.71	0.70	0.00
Chiromyscus_chiropus	0.18	0.8	0.73	0.00
Chironax_melanocephalus	1.52	4.8	0.88	0.00
Chironectes_minimus	0.83	1.57	0.74	0.00
Chiropodomys_gliroides	1.13	3.17	0.82	0.00
Chiropodomys_karlkoopmani	0	0	-0.36	0.03
Chiropotes_albinasus	0.03	0.1	0.74	0.00
Chiropotes_chiropotes	0.04	0.08	0.70	0.00
Chiropotes_satanas	0.4	1	0.73	0.00
Chiropotes_utahickae	0.04	0.19	0.78	0.00
Chiruromys_forbesi	0.08	0.11	0.17	0.29
Chiruromys_lamia	0.04	0.04	0.03	0.88
Chiruromys_vates	0.03	0.09	0.30	0.06
Chlorocebus_aethiops	0.07	0.2	0.73	0.00
Chlorocebus_cynosuros	0.05	0.15	0.78	0.00
Chlorocebus_djamdjamensis	0.01	0.05	0.53	0.00
Chlorocebus_pygerythrus	0.19	0.37	0.67	0.00
Chlorocebus_sabaeus	0.07	0.21	0.74	0.00
Chlorocebus_tantalus	0.08	0.15	0.68	0.00
Chodsigoa_hypsibia	0.32	1.12	0.78	0.00
Chodsigoa_lamula	0.05	0.91	0.79	0.00

Chodsigoa_parca	0.32	1.43	0.82	0.00
Chodsigoa_smithii	0.11	0.8	0.75	0.00
Choeroniscus_godmani	1.04	1.72	0.59	0.00
Choeroniscus_minor	0.17	0.39	0.74	0.00
Choeroniscus_periosus	0.16	0.34	0.60	0.00
Choeronycteris_mexicana	1.85	2.86	0.67	0.00
Choeropsis_liberiensis	2.04	1.67	-0.34	0.03
Choloepus_didactylus	0.06	0.15	0.77	0.00
Choloepus_hoffmanni	0.37	0.68	0.55	0.00
Chrotogale_owstoni	0.14	0.78	0.70	0.00
Chrotomys_gonzalesi	0	0.05	0.14	0.45
Chrotomys_mindorensis	4.11	6.58	0.67	0.00
Chrotomys_silaceus	0.02	0.14	0.34	0.03
Chrotomys_whiteheadi	0.22	0.63	0.57	0.00
Chrotopterus_auritus	0.53	1.06	0.76	0.00
Chrysocyon_brachyurus	0.58	1.27	0.77	0.00
Cistugo_lesueuri	0.3	0.4	0.44	0.01
Cistugo_seabrae	0.03	0.06	0.43	0.01
Civettictis_civetta	0.15	0.25	0.69	0.00
Cloeotis_percivali	0.76	1.58	0.73	0.00
Clyomys_laticeps	0.78	1.8	0.77	0.00
Coccymys_ruemmleri	0.04	0.09	0.39	0.01
Coelops_frithii	1.26	3.02	0.76	0.00
Coelops_robinsoni	0.05	0.22	0.56	0.00
Coendou_bicolor	0.25	0.55	0.70	0.00
Coendou_prehensilis	0.49	0.97	0.76	0.00
Coendou_rothschildi	0.76	1.84	0.73	0.00
Coleura_afra	0.05	0.11	0.70	0.00
Coleura_seychellensis	8.4	12.85	0.73	0.00
Colobus_angolensis	0.03	0.06	0.68	0.00
Colobus_guereza	0.03	0.07	0.65	0.00
Colobus_polykomos	0.05	0.12	0.66	0.00
Colobus_satanas	0.19	0.33	0.56	0.00
Colobus_vellerosus	0.33	0.89	0.70	0.00
Colomys_goslingi	0.08	0.17	0.70	0.00
Condylura_cristata	5.91	6.21	0.13	0.41
Conepatus_chinga	0.38	0.9	0.79	0.00
Conepatus_humboldtii	0.14	0.3	0.65	0.00
Conepatus_leuconotus	1.87	2.79	0.64	0.00
Conepatus_semistriatus	1.6	2.87	0.70	0.00
Congosorex_phillipsorum	0	0	-0.12	0.55
Congosorex_verheyeni	0	0.01	0.43	0.01
Conilurus_penicillatus	0.05	0.09	0.56	0.00
Connochaetes_gnou	1.48	1.96	0.65	0.00
Connochaetes_taurinus	0.32	0.53	0.70	0.00
Cormura_brevirostris	0.19	0.38	0.72	0.00

Corynorhinus_mexicanus	3.38	5.01	0.68	0.00
Corynorhinus_rafinesquii	8.56	10.56	0.55	0.00
Corynorhinus_townsendii	2.19	2.61	0.50	0.00
Craseonycteris_thonglongyai	0.55	1.3	0.64	0.00
Crateromys_australis	0.01	0.1	0.36	0.03
Crateromys_heaneyi	0.01	0.09	0.32	0.05
Crateromys_schadenbergi	0.01	0.13	0.50	0.00
Cremnomys_cutchicus	2.23	5.77	0.82	0.00
Cremnomys_elvira	1.04	5.69	0.68	0.00
Cricetomys_emini	0.36	0.44	0.33	0.04
Cricetomys_gambianus	0.1	0.23	0.74	0.00
Cricetulus_alticola	0	0	0.48	0.00
Cricetulus_barabensis	1.14	2.81	0.83	0.00
Cricetulus_kamensis	0.03	0.17	0.78	0.00
Cricetulus_lama	0.1	0.69	0.80	0.00
Cricetulus_longicaudatus	0.52	1.32	0.74	0.00
Cricetulus_migratorius	1.47	2.09	0.59	0.00
Cricetulus_sokolovi	0.11	0.36	0.76	0.00
Cricetulus_tibetanus	0	0	0.52	0.00
Cricetus_cricetus	2.81	3.75	0.46	0.00
Crocidura_aleksandrisi	1.43	3.01	0.76	0.00
Crocidura_allex	0.27	0.91	0.54	0.00
Crocidura_andamanensis	0.02	0.05	0.16	0.36
Crocidura_ansellorum	0	0.03	0.29	0.10
Crocidura_arabica	6.07	17.04	0.91	0.00
Crocidura_arispa	0.33	1.1	0.44	0.01
Crocidura_attenuata	0.99	3.02	0.85	0.00
Crocidura_attila	0.04	0.08	0.65	0.00
Crocidura_baileyi	0	0.05	0.69	0.00
Crocidura_baluensis	1.14	1.05	-0.11	0.49
Crocidura_batesi	0.12	0.19	0.50	0.00
Crocidura_beatus	0.25	0.68	0.45	0.00
Crocidura_beccarii	0.15	0.49	0.48	0.00
Crocidura_bottegoides	0.03	0.07	0.56	0.00
Crocidura_brunnea	5.11	10.17	0.75	0.00
Crocidura_buettikoferi	12.48	9.32	-0.54	0.00
Crocidura_caliginea	0.01	0.02	0.32	0.04
Crocidura_canariensis	7.41	13.7	0.79	0.00
Crocidura_cinderella	0.04	0.11	0.75	0.00
Crocidura_congobelgica	0	0.01	0.27	0.10
Crocidura_crenata	0.06	0.14	0.67	0.00
Crocidura_crossei	1.11	1.26	0.14	0.38
Crocidura_cyanea	0.52	0.84	0.69	0.00
Crocidura_denti	0.03	0.06	0.63	0.00
Crocidura_desperata	0.18	0.25	0.35	0.03
Crocidura_dolichura	0.72	0.66	-0.27	0.10

Crocidura_dsinezumi	14.05	15.73	0.51	0.00
Crocidura_eisentrauti	0.21	0.08	0.20	0.25
Crocidura_elgonius	0.48	1	0.61	0.00
Crocidura_elongata	0.14	0.46	0.57	0.00
Crocidura_flavescens	1.37	2.2	0.69	0.00
Crocidura_foetida	0.24	0.57	0.71	0.00
Crocidura_foxi	0.07	0.16	0.72	0.00
Crocidura_fuliginosa	0.57	2.03	0.84	0.00
Crocidura_fulvastra	0.04	0.08	0.68	0.00
Crocidura_fumosa	0.06	0.38	0.67	0.00
Crocidura_fuscomurina	0.08	0.17	0.72	0.00
Crocidura_glassi	0.04	0.1	0.56	0.00
Crocidura_gmelini	0.7	0.91	0.55	0.00
Crocidura_goliath	0.05	0.15	0.77	0.00
Crocidura_grandiceps	2.91	5.16	0.75	0.00
Crocidura_grassei	0.03	0.09	0.69	0.00
Crocidura_grayi	1.48	2.77	0.63	0.00
Crocidura_greenwoodi	0	0	0.05	0.79
Crocidura_harenna	0	0	-0.12	0.55
Crocidura_hikmiya	1.57	7.42	0.72	0.00
Crocidura_hildegardeae	0.05	0.1	0.58	0.00
Crocidura_hirta	0.16	0.32	0.73	0.00
Crocidura_hispida	0	0.14	0.42	0.01
Crocidura_hutanis	1	1.64	0.39	0.01
Crocidura_indochinensis	0.4	1.6	0.78	0.00
Crocidura_jacksoni	0.1	0.22	0.68	0.00
Crocidura_jenkinsi	0.16	0.14	0.21	0.20
Crocidura_jouvenetae	0.18	0.61	0.70	0.00
Crocidura_kivuana	0	0.17	0.62	0.00
Crocidura_lamottei	0.14	0.31	0.76	0.00
Crocidura_lanosa	0.01	0.04	0.53	0.00
Crocidura_lasiura	2.57	5.78	0.88	0.00
Crocidura_latona	0.01	0.01	0.38	0.02
Crocidura_lea	0.14	0.47	0.55	0.00
Crocidura_lepidura	0.77	1.43	0.50	0.00
Crocidura_leucodon	4.87	7.62	0.81	0.00
Crocidura_levicula	0.12	0.39	0.70	0.00
Crocidura_littoralis	0.02	0.04	0.53	0.00
Crocidura_lucina	0	0.01	0.35	0.03
Crocidura_ludia	0	0.01	0.55	0.00
Crocidura_luna	0.09	0.19	0.66	0.00
Crocidura_lusitania	0.04	0.12	0.70	0.00
Crocidura_macarthuri	0.01	0.01	0.30	0.06
Crocidura_macmillani	0	0.13	0.75	0.00
Crocidura_malayana	2.76	8.07	0.93	0.00
Crocidura_manengubae	1.44	2.65	0.77	0.00

Crocidura_maquassiensis	2.23	3.4	0.73	0.00
Crocidura_mariquensis	0.33	0.62	0.72	0.00
Crocidura_maurisca	0.02	0.05	0.54	0.00
Crocidura_maxi	3.17	6.37	0.73	0.00
Crocidura_miya	7.39	12.88	0.70	0.00
Crocidura_monax	0.39	0.95	0.54	0.00
Crocidura_monticola	0.9	1.87	0.62	0.00
Crocidura_montis	0.06	0.36	0.58	0.00
Crocidura_muricauda	0.26	0.75	0.66	0.00
Crocidura_nana	0	0.61	0.55	0.00
Crocidura_nanilla	0.03	0.09	0.74	0.00
Crocidura_negligens	2.38	7.32	0.92	0.00
Crocidura_negrina	0.38	0.96	0.50	0.00
Crocidura_nicobarica	0	0.05	0.08	0.69
Crocidura_nigeriae	4.63	4.09	-0.34	0.03
Crocidura_nigricans	0.02	0.2	0.70	0.00
Crocidura_nigripes	0.16	0.54	0.53	0.00
Crocidura_nigrofusca	0.05	0.11	0.67	0.00
Crocidura_nimbae	0.03	0.09	0.46	0.00
Crocidura_niobe	0.13	0.27	0.48	0.00
Crocidura_obscurior	0.24	0.73	0.67	0.00
Crocidura_olivieri	0.19	0.32	0.76	0.00
Crocidura_orientalis	10.8	12.45	0.14	0.38
Crocidura_orii	2.2	2.4	0.12	0.45
Crocidura_pachyura	4.28	7.93	0.84	0.00
Crocidura_palawanensis	0.06	0.25	0.57	0.00
Crocidura_paradoxura	0.12	0.43	0.49	0.00
Crocidura_parvipes	0.04	0.08	0.64	0.00
Crocidura_pasha	0.16	0.39	0.72	0.00
Crocidura_phaeura	0.08	0.29	0.61	0.00
Crocidura_picea	0.05	0.17	0.25	0.12
Crocidura_poensis	1.14	1.31	0.16	0.32
Crocidura_ramona	3.56	6.54	0.70	0.00
Crocidura_rhoditis	0.15	0.48	0.54	0.00
Crocidura_roosevelti	0.01	0.04	0.66	0.00
Crocidura_russula	6.84	10.49	0.89	0.00
Crocidura_serezkyensis	2.62	2.39	-0.07	0.69
Crocidura_shantungensis	1.91	5.03	0.87	0.00
Crocidura_sibirica	0.34	0.53	0.50	0.00
Crocidura_sicula	13.09	16.54	0.74	0.00
Crocidura_silacea	1.75	2.99	0.74	0.00
Crocidura_smithii	0.01	0.04	0.57	0.00
Crocidura_somalica	0	0.02	0.64	0.00
Crocidura_stenocephala	0.05	0.21	0.40	0.01
Crocidura_suaveolens	2.08	3.28	0.70	0.00
Crocidura_tanakae	16.87	23.92	0.82	0.00

Crocidura_tansaniana	0.06	0.07	0.02	0.93
Crocidura_tarella	0.01	0.11	0.32	0.05
Crocidura_telfordi	0.12	0.1	-0.32	0.04
Crocidura_thalia	0.01	0.09	0.74	0.00
Crocidura_theresae	0.18	0.57	0.67	0.00
Crocidura_thomensis	0.8	1.99	0.60	0.00
Crocidura_trichura	1.26	3.86	0.68	0.00
Crocidura_turba	0.07	0.15	0.72	0.00
Crocidura_usambarae	0.05	0.08	0.13	0.41
Crocidura_viaria	0.04	0.11	0.70	0.00
Crocidura_voi	0.06	0.11	0.64	0.00
Crocidura_vorax	0.39	1.55	0.80	0.00
Crocidura_watasei	17.32	15.17	-0.44	0.01
Crocidura_whitakeri	1.36	3.29	0.82	0.00
Crocidura_wimmeri	24.08	34.79	0.79	0.00
Crocidura_xantippe	0.02	0.04	0.22	0.17
Crocidura_yankariensis	0.02	0.06	0.69	0.00
Crocidura_zarudnyi	0.14	0.41	0.70	0.00
Crocidura_zimmermanni	3.69	6.15	0.62	0.00
Crocuta_crocuta	0.07	0.18	0.72	0.00
Crossarchus_alexandri	0	0.01	0.65	0.00
Crossarchus_obscurus	0.27	0.73	0.69	0.00
Crossarchus_platycephalus	2.07	1.87	-0.30	0.07
Crossomys_moncktoni	0.06	0.13	0.36	0.02
Crunomys_melanius	0.52	1.58	0.47	0.00
Cryptonanus_chacoensis	0.61	1.13	0.77	0.00
Cryptoprocta_ferox	0.01	0.02	0.52	0.00
Cryptotis_colombiana	11.53	12.29	0.11	0.49
Cryptotis_endersi	0.86	0.8	-0.16	0.32
Cryptotis_equatoris	2.29	5.1	0.86	0.00
Cryptotis_goldmani	1.14	2.11	0.57	0.00
Cryptotis_goodwini	1.35	4.04	0.80	0.00
Cryptotis_gracilis	4.3	6.21	0.63	0.00
Cryptotis_griseoventris	1.27	3.55	0.75	0.00
Cryptotis_magna	1.08	1.64	0.54	0.00
Cryptotis_mayensis	0.86	1.52	0.57	0.00
Cryptotis_medellinia	5.4	6.32	0.32	0.04
Cryptotis_mera	0	0	-0.12	0.55
Cryptotis_meridensis	2.32	3.04	0.52	0.00
Cryptotis_merriami	0.44	1.68	0.69	0.00
Cryptotis_mexicana	2.37	4.01	0.58	0.00
Cryptotis_montivaga	1.33	4.21	0.84	0.00
Cryptotis_nelsoni	0	0.1	0.11	0.56
Cryptotis_nigrescens	3.12	4.8	0.63	0.00
Cryptotis_obscura	1.39	2.93	0.53	0.00
Cryptotis_parva	7.33	8.85	0.60	0.00

Cryptotis_phillipsii	0.28	0.96	0.59	0.00
Cryptotis_squamipes	0.77	1.62	0.60	0.00
Cryptotis_tamensis	0.68	1.27	0.48	0.00
Cryptotis_thomasi	0.81	1.23	0.30	0.07
Ctenodactylus_gundi	0.92	1.4	0.57	0.00
Cuniculus_paca	0.49	1	0.75	0.00
Cuniculus_taczanowskii	0.77	1.32	0.63	0.00
Cuon_alpinus	0.69	1.79	0.80	0.00
Cyclopes_didactylus	0.24	0.48	0.69	0.00
Cynictis_penicillata	0.49	0.75	0.70	0.00
Cynocephalus_volans	0.34	0.89	0.46	0.00
Cynogale_bennettii	0.91	2.28	0.79	0.00
Cynomops_greenhalli	0.65	1.13	0.70	0.00
Cynomops_mexicanus	1.5	2.67	0.60	0.00
Cynomops_planirostris	0.36	0.73	0.74	0.00
Cynomys_gunnisoni	1.35	1.71	0.60	0.00
Cynomys_leucurus	1	1.17	0.19	0.24
Cynomys_ludovicianus	1.54	1.85	0.39	0.01
Cynomys_mexicanus	0.15	0.52	0.54	0.00
Cynomys_parvidens	1.12	1.56	0.48	0.00
Cynopterus_brachyotis	1.24	3.3	0.83	0.00
Cynopterus_horsfieldii	1.08	2.52	0.77	0.00
Cynopterus_luzoniensis	0.63	1.35	0.65	0.00
Cynopterus_minutus	0.81	1.66	0.61	0.00
Cynopterus_nusatenggara	0.45	1.08	0.66	0.00
Cynopterus_sphinx	1.53	3.38	0.78	0.00
Cynopterus_titthaecheilus	1.68	3.3	0.64	0.00
Cyttarops_alecto	1.03	1.7	0.64	0.00
Dactylomys_boliviensis	0.05	0.06	0.03	0.88
Dactylomys_dactylinus	0.04	0.11	0.77	0.00
Dactylonax_palpator	0.04	0.09	0.38	0.02
Dactylopsila_megalura	0.03	0.09	0.60	0.00
Dactylopsila_tatei	0	0	-0.12	0.55
Dactylopsila_trivirgata	0.05	0.1	0.51	0.00
Dama_dama	7.04	10.7	0.85	0.00
Dama_mesopotamica	4.16	7.54	0.88	0.00
Damaliscus_lunatus	0.15	0.31	0.70	0.00
Damaliscus_pygargus	1.98	2.85	0.72	0.00
Dasycercus_blythi	0.02	0.04	0.46	0.00
Dasycercus_cristicauda	0	0	0.35	0.05
Dasykaluta_rosamondae	0.04	0.14	0.70	0.00
Dasymys_incomtus	0.18	0.31	0.71	0.00
Dasymys_montanus	0.01	0	-0.20	0.24
Dasymys_rufulus	0.76	0.89	0.26	0.11
Dasyprocta_coibae	0	0	-0.12	0.55
Dasyprocta_fuliginosa	0.09	0.16	0.65	0.00

Dasyprocta_guamara	0.15	0.31	0.53	0.00
Dasyprocta_leporina	0.2	0.45	0.75	0.00
Dasyprocta_mexicana	2.86	4.68	0.70	0.00
Dasyprocta_prymnolopha	0.65	1.4	0.72	0.00
Dasyprocta_punctata	0.79	1.45	0.64	0.00
Dasyprocta_ruatanica	2.51	10.21	0.88	0.00
Dasypus_hybridus	0.91	1.81	0.75	0.00
Dasypus_kappleri	0.05	0.14	0.76	0.00
Dasypus_novemcinctus	1.23	1.9	0.70	0.00
Dasypus_pilosus	0.1	0.29	0.67	0.00
Dasypus_sabanicola	0.58	1.26	0.70	0.00
Dasypus_septemcinctus	0.58	1.27	0.80	0.00
Dasyuroides_byrnei	0	0	0.30	0.06
Dasyurus_albopunctatus	0.05	0.1	0.55	0.00
Dasyurus_geoffroii	0.18	0.28	0.45	0.00
Dasyurus_hallucatus	0.18	0.37	0.68	0.00
Dasyurus_maculatus	1.29	1.68	0.60	0.00
Dasyurus_spartacus	0.02	0.06	0.63	0.00
Dasyurus_viverrinus	0.4	0.54	0.36	0.02
Daubentonia_madagascariensis	0	0.01	0.53	0.00
Delanymys_brooksi	0.2	0.63	0.62	0.00
Delomys_collinus	3.44	6.46	0.72	0.00
Delomys_dorsalis	2.57	4.62	0.77	0.00
Delomys_sublineatus	4.18	7.25	0.75	0.00
Deltamys_kempi	2.51	3.52	0.69	0.00
Dendrogale_murina	0.57	2.82	0.89	0.00
Dendrohyrax_arboreus	0.13	0.25	0.73	0.00
Dendrohyrax_dorsalis	0.41	0.48	0.20	0.22
Dendrohyrax_validus	0.23	0.4	0.44	0.01
Dendrolagus_bennettianus	0.01	0	-0.16	0.35
Dendrolagus_dorianus	0.01	0.05	0.40	0.01
Dendrolagus_goodfellowi	0.06	0.11	0.25	0.12
Dendrolagus_inustus	0.09	0.17	0.64	0.00
Dendrolagus_lumholtzi	0.08	0.25	0.36	0.02
Dendrolagus_matschiei	0	0	-0.32	0.08
Dendrolagus_mayri	0	0	-0.12	0.55
Dendrolagus_mbaiso	0.1	0.39	0.70	0.00
Dendrolagus_notatus	0.08	0.14	0.27	0.10
Dendrolagus_pulcherrimus	0	0	-0.12	0.55
Dendrolagus_scottae	0	0	-0.12	0.55
Dendrolagus_spadix	0.06	0.15	0.31	0.05
Dendrolagus_stellarum	0.05	0.13	0.64	0.00
Dendrolagus_ursinus	0.01	0.01	0.43	0.01
Dendromus_insignis	0.1	0.38	0.66	0.00
Dendromus_kahuziensis	0	0	-0.12	0.55
Dendromus_kivu	0.08	0.19	0.52	0.00

Dendromus_lovati	0.27	0.59	0.72	0.00
Dendromus_melanotis	0.23	0.44	0.70	0.00
Dendromus_mesomelas	1.08	1.54	0.67	0.00
Dendromus_messorius	0.03	0.05	0.35	0.03
Dendromus_mystacalis	0.21	0.45	0.71	0.00
Dendromus_nyikae	0.09	0.17	0.61	0.00
Dendromus_oreas	0.09	0.09	0.29	0.07
Deomys_ferrugineus	0.09	0.19	0.74	0.00
Dephomys_defua	0.24	0.73	0.69	0.00
Desmana_moschata	3.93	4.66	0.10	0.57
Desmodilliscus_braueri	0.03	0.08	0.70	0.00
Desmodillus_auricularis	0.38	0.54	0.67	0.00
Desmodus_rotundus	0.65	1.25	0.73	0.00
Desmomys_harringtoni	0.06	0.18	0.74	0.00
Desmomys_yaldeni	0	0.06	0.64	0.00
Diaemus_youngi	0.54	1.04	0.75	0.00
Dicerorhinus_sumatrensis	0.01	0	-0.22	0.16
Diceros_bicornis	0.22	0.38	0.72	0.00
Diclidurus_albus	0.46	0.92	0.71	0.00
Diclidurus_isabellus	0.01	0.04	0.62	0.00
Diclidurus_scutatus	0.05	0.13	0.77	0.00
Dicrostonyx_groenlandicus	0.02	0.05	0.38	0.02
Dicrostonyx_hudsonius	0.03	0.04	0.19	0.24
Dicrostonyx_nelsoni	0.04	0.05	0.09	0.61
Dicrostonyx_nunatakensis	0	0	0.00	1.00
Dicrostonyx_richardsoni	0.01	0.01	-0.10	0.53
Dicrostonyx_torquatus	0.16	0.32	0.50	0.00
Didelphis_albiventris	0.67	1.42	0.80	0.00
Didelphis_aurita	1.91	3.92	0.79	0.00
Didelphis_imperfecta	0.02	0.04	0.52	0.00
Didelphis_marsupialis	0.46	0.85	0.68	0.00
Didelphis_pernigra	1.01	1.85	0.70	0.00
Didelphis_virginiana	6.1	7.32	0.58	0.00
Dinaromys_bogdanovi	0.97	3.46	0.73	0.00
Dinomys_branickii	0.14	0.26	0.58	0.00
Diphylla_ecaudata	0.9	1.67	0.73	0.00
Diplogale_hosei	0.09	0.13	0.39	0.01
Diplomesodon_pulchellum	0.48	0.66	0.49	0.00
Diplomys_labilis	0.37	0.75	0.74	0.00
Diplothrix_legata	2.74	2.55	0.14	0.38
Dipodomys_agilis	13.66	13.82	0.17	0.29
Dipodomys_californicus	3.38	3.28	-0.06	0.74
Dipodomys_compactus	2.48	4.17	0.47	0.00
Dipodomys_deserti	2.3	2.91	0.62	0.00
Dipodomys_elator	3.08	3.16	0.00	1.00
Dipodomys_gravipes	1.47	3.12	0.72	0.00

Dipodomys_heermanni	9.87	10.69	0.43	0.01
Dipodomys_ingens	5.46	5.95	0.11	0.49
Dipodomys_insularis	0	0	-0.12	0.55
Dipodomys_margaritae	0.11	0.01	-0.38	0.03
Dipodomys_merriami	1.33	1.8	0.62	0.00
Dipodomys_microps	0.79	0.93	0.51	0.00
Dipodomys_nelsoni	0.55	0.9	0.68	0.00
Dipodomys_nitratoides	9.73	10.92	0.40	0.01
Dipodomys_ordii	1.4	1.78	0.54	0.00
Dipodomys_panamintinus	4.92	5.17	0.30	0.07
Dipodomys_phillipsii	6.16	8.87	0.68	0.00
Dipodomys_simulans	9.94	10.66	0.46	0.00
Dipodomys_spectabilis	1.36	1.86	0.55	0.00
Dipodomys_stephensi	27.99	33.23	0.57	0.00
Dipodomys_venustus	15.23	14.63	-0.19	0.24
Dipus_sagitta	0.19	0.5	0.78	0.00
Distoechurus_pennatus	0.04	0.09	0.48	0.00
Dobsonia_anderseni	0.03	0.07	0.24	0.14
Dobsonia_beauforti	0.51	0.51	0.05	0.79
Dobsonia_chapmani	3	2.7	-0.23	0.16
Dobsonia_crenulata	0.18	0.54	0.58	0.00
Dobsonia_emersa	0.41	0.47	0.21	0.19
Dobsonia_exoleta	0.21	0.6	0.61	0.00
Dobsonia_inermis	0.03	0.05	0.30	0.07
Dobsonia_minor	0.04	0.09	0.54	0.00
Dobsonia_moluccensis	0.05	0.1	0.55	0.00
Dobsonia_pannietensis	0.14	0	-0.59	0.00
Dobsonia_peronii	0.44	1.06	0.66	0.00
Dobsonia_praedatrix	0.03	0.05	0.13	0.41
Dobsonia_viridis	0.1	0.19	0.39	0.01
Dolichotis_patagonum	0.21	0.54	0.75	0.00
Dolichotis_salinicola	0.22	0.46	0.77	0.00
Dorcatragus_megalotis	0	0.01	0.33	0.04
Dorcopsis_atrata	0	0	-0.12	0.55
Dorcopsis_hageni	0.02	0.06	0.64	0.00
Dorcopsis_luctuosa	0.05	0.07	0.43	0.01
Dorcopsis_muelleri	0.09	0.17	0.60	0.00
Dorcopsulus_macleayi	0	0	0.19	0.27
Dorcopsulus_vanheurni	0.04	0.07	0.37	0.02
Dremomys_everetti	0.07	0.08	0.12	0.45
Dremomys_gularis	0.28	1.26	0.76	0.00
Dremomys_lokriah	0.09	0.34	0.74	0.00
Dremomys_pernyi	0.66	2.16	0.81	0.00
Dremomys_pyrrhomerus	0.42	1.92	0.80	0.00
Dremomys_rufigenis	0.36	1.49	0.81	0.00
Dromiciops_gliroides	0.49	1.33	0.76	0.00

Dryomys_niethammeri	0.02	0.03	-0.09	0.61
Dryomys_nitedula	2.24	3.85	0.75	0.00
Dyacopterus_brooksi	0.75	1.42	0.49	0.00
Dyacopterus_spadiceus	0.78	1.91	0.72	0.00
Dymecodon_pilirostris	4.62	4.87	0.07	0.69
Echimys_chrysurus	0.09	0.24	0.76	0.00
Echinoprocta_rufescens	1.93	2.74	0.46	0.00
Echinops_telfairi	0	0.01	0.32	0.04
Echinosorex_gymnura	0.71	1.82	0.75	0.00
Echiothrix_centrosa	0.11	0.5	0.58	0.00
Echiothrix_leucura	0.59	1.65	0.49	0.00
Echymipera_clara	0.02	0.05	0.64	0.00
Echymipera_davidi	0	0	-0.26	0.17
Echymipera_kalubu	0.04	0.09	0.55	0.00
Echymipera_rufescens	0.04	0.08	0.55	0.00
Ectophylla_alba	0.11	0.38	0.62	0.00
Eidolon_dupreanum	0.02	0.04	0.46	0.00
Eidolon_helvum	0.27	0.43	0.73	0.00
Eira_barbara	0.62	1.17	0.73	0.00
Elaphodus_cephalophus	0.6	2.37	0.81	0.00
Elephantulus_brachyrhynchus	0.22	0.38	0.74	0.00
Elephantulus_edwardii	0.66	0.89	0.59	0.00
Elephantulus_intufi	0.06	0.16	0.71	0.00
Elephantulus_myurus	0.86	1.37	0.70	0.00
Elephantulus_rozeti	1.11	2.71	0.78	0.00
Elephantulus_rufescens	0.05	0.11	0.62	0.00
Elephantulus_rupestris	0.15	0.22	0.47	0.00
Elephas_maximus	0.41	1.24	0.73	0.00
Eligmodontia_moreni	0.23	0.74	0.72	0.00
Eligmodontia_morgani	0.09	0.16	0.51	0.00
Eligmodontia_puerulus	0.09	0.25	0.74	0.00
Eligmodontia_typus	0.24	0.49	0.69	0.00
Eliomys_melanurus	2.59	5.21	0.78	0.00
Eliomys_munbyanus	1.42	3.17	0.79	0.00
Eliomys_quercinus	6.99	10.8	0.89	0.00
Eliurus_grandidieri	0	0.01	0.51	0.00
Eliurus_majori	0	0.02	0.55	0.00
Eliurus_minor	0.01	0.02	0.56	0.00
Eliurus_myoxinus	0.01	0.02	0.38	0.02
Eliurus_penicillatus	0	0	-0.12	0.55
Eliurus_petteri	0	0	0.25	0.15
Eliurus_tanala	0	0.02	0.55	0.00
Eliurus_webbi	0	0.02	0.54	0.00
Emballonura_alecto	0.38	0.85	0.69	0.00
Emballonura_atrata	0.01	0.04	0.51	0.00
Emballonura_beccarii	0.04	0.08	0.44	0.01

Emballonura_dianae	0.07	0.17	0.34	0.03
Emballonura_monticola	0.99	2.29	0.77	0.00
Emballonura_raffrayana	0.07	0.14	0.32	0.04
Emballonura_semicaudata	2.3	2.34	0.01	0.98
Emballonura_serii	0.04	0.07	0.20	0.22
Emballonura_tiavato	0.01	0.02	0.19	0.24
Enchisthenes_hartii	1.19	2.04	0.65	0.00
Enhydra_lutris	0.03	0.02	-0.39	0.01
Eoglaucomys_fimbriatus	0.38	1.11	0.72	0.00
Eolagurus_luteus	0.3	0.98	0.79	0.00
Eolagurus_przewalskii	0.03	0.11	0.80	0.00
Eonycteris_robusta	1.04	1.87	0.60	0.00
Eonycteris_spelaea	0.85	2.23	0.78	0.00
Eothenomys_cachinus	0.03	0.56	0.82	0.00
Eothenomys_chinensis	0.11	1.02	0.75	0.00
Eothenomys_custos	0.08	0.82	0.85	0.00
Eothenomys_melanogaster	0.59	2.41	0.84	0.00
Eothenomys_miletus	0.42	1.79	0.82	0.00
Eothenomys_olitor	0.35	1.62	0.85	0.00
Eothenomys_proditor	0.08	0.69	0.83	0.00
Eothenomys_wardi	0.03	0.69	0.81	0.00
Eozapus_setchuanus	0.05	0.34	0.80	0.00
Episoriculus_caudatus	0.16	0.99	0.81	0.00
Episoriculus_fumidus	2.56	4.44	0.74	0.00
Episoriculus_leucops	0.06	0.32	0.79	0.00
Episoriculus_macrurus	0.23	1.26	0.80	0.00
Epixerus_ebii	0.25	0.6	0.67	0.00
Epomophorus_angolensis	0.06	0.35	0.79	0.00
Epomophorus_crypturus	0.2	0.38	0.64	0.00
Epomophorus_gambianus	0.12	0.25	0.72	0.00
Epomophorus_labiatus	0.06	0.13	0.69	0.00
Epomophorus_minimus	0.09	0.25	0.64	0.00
Epomophorus_wahlbergi	0.23	0.44	0.77	0.00
Epomops_buettikoferi	0.43	0.79	0.70	0.00
Epomops_dobsonii	0.07	0.15	0.70	0.00
Epomops_franqueti	0.29	0.34	0.16	0.32
Eptesicus_andinus	1.84	2.93	0.68	0.00
Eptesicus_bobrinskoi	0.21	0.51	0.61	0.00
Eptesicus_bottae	1.99	3.53	0.80	0.00
Eptesicus_brasiliensis	0.53	1.06	0.75	0.00
Eptesicus_chiriquinus	1.65	2.56	0.62	0.00
Eptesicus_floweri	0.06	0.14	0.72	0.00
Eptesicus_furinalis	0.59	1.15	0.74	0.00
Eptesicus_fuscus	3.93	4.59	0.43	0.01
Eptesicus_gobiensis	0.34	0.58	0.73	0.00
Eptesicus_guadeloupensis	14.34	21.36	0.76	0.00

Eptesicus_hottentotus	0.4	0.65	0.70	0.00
Eptesicus_innoxius	1.14	2.75	0.76	0.00
Eptesicus_japonensis	7.13	7.37	0.01	0.98
Eptesicus_malagasyensis	0	0	-0.03	0.87
Eptesicus_matroka	0.14	0.24	0.64	0.00
Eptesicus_nasutus	1.81	3.53	0.80	0.00
Eptesicus_nilssonii	2.3	3.27	0.50	0.00
Eptesicus_pachyotis	0.2	0.82	0.83	0.00
Eptesicus_serotinus	3.35	5.53	0.85	0.00
Equus_africanus	0.01	0.02	0.45	0.00
Equus_ferus	0	0	0.20	0.27
Equus_grevyi	0.01	0.02	0.50	0.00
Equus_hemionus	0.26	0.54	0.51	0.00
Equus_kiang	0.01	0.03	0.66	0.00
Equus_quagga	0.44	0.75	0.73	0.00
Equus_zebra	0.27	0.38	0.64	0.00
Eremodipus_lichtensteini	0.37	0.71	0.59	0.00
Erethizon_dorsatum	1.44	1.54	0.07	0.69
Erinaceus_amurensis	1.71	4.34	0.87	0.00
Erinaceus_concolor	2.23	4.44	0.77	0.00
Erinaceus_europaeus	7.52	10.62	0.82	0.00
Erinaceus_roumanicus	2.95	4.35	0.58	0.00
Eropeplus_canus	0	0.01	0.24	0.14
Erophylla_bombifrons	4.53	6.02	0.55	0.00
Erophylla_sezekorni	1.58	3.26	0.56	0.00
Erythrocebus_patas	0.06	0.13	0.70	0.00
Euchoreutes_naso	0.13	0.43	0.81	0.00
Euderma_maculatum	1.19	1.46	0.48	0.00
Eudorcas_albonotata	0	0	0.40	0.02
Eudorcas_rufifrons	0.03	0.07	0.65	0.00
Eudorcas_thomsonii	0.2	0.39	0.70	0.00
Eulemur_albifrons	0	0	-0.01	1.00
Eulemur_cinereiceps	0	0	-0.21	0.27
Eulemur_collaris	0	0.08	0.36	0.03
Eulemur_coronatus	0	0.01	0.27	0.11
Eulemur_flavifrons	0	0	-0.12	0.55
Eulemur_fulvus	0.01	0.1	0.75	0.00
Eulemur_macaco	0.02	0.03	0.19	0.24
Eulemur_mongoz	0.1	0.15	0.23	0.16
Eulemur_rubriventer	0	0.01	0.27	0.10
Eulemur_rufifrons	0	0	0.07	0.68
Eulemur_sanfordi	0.06	0.09	0.32	0.04
Eumops_auripendulus	0.55	1.08	0.73	0.00
Eumops_bonariensis	0.57	1.11	0.75	0.00
Eumops_dabbenei	0.61	0.96	0.62	0.00
Eumops_floridanus	34.6	36.82	0.58	0.00

Eumops_glaucinus	0.58	1.09	0.72	0.00
Eumops_hansae	0.28	0.49	0.63	0.00
Eumops_patagonicus	0.62	1.23	0.79	0.00
Eumops_perotis	0.65	1.14	0.74	0.00
Eumops_trumbulli	0.04	0.11	0.76	0.00
Eumops_underwoodi	1.83	3.04	0.66	0.00
Euneomys_mordax	0.08	0.27	0.76	0.00
Euneomys_petersoni	0.09	0.2	0.58	0.00
Euoticus_elegantulus	0.11	0.25	0.71	0.00
Euoticus_pallidus	4.32	3.56	-0.40	0.01
Eupetaurus_cinereus	0.02	0.05	0.47	0.00
Euphractus_sexcinctus	0.62	1.32	0.80	0.00
Eupleres_goudotii	0	0.02	0.41	0.01
Euryoryzomys_lamia	1.32	2.77	0.78	0.00
Euryoryzomys_legatus	0.42	0.91	0.69	0.00
Euryoryzomys_macconnelli	0.07	0.18	0.78	0.00
Euryoryzomys_nitidus	0.08	0.15	0.69	0.00
Euryoryzomys_russatus	2.27	4.65	0.82	0.00
Exilisciurus_concinnus	0.34	0.89	0.46	0.00
Exilisciurus_whiteheadi	0.04	0.09	0.50	0.00
Falsistrellus_affinis	0.56	1.29	0.76	0.00
Falsistrellus_mackenziei	0.69	1.05	0.43	0.01
Falsistrellus_tasmaniensis	1.58	2.04	0.64	0.00
Felis_chaus	2.04	4.1	0.77	0.00
Felis_margarita	0.65	1.04	0.69	0.00
Felis_nigripes	0.42	0.63	0.68	0.00
Felis_silvestris	0.71	1.29	0.75	0.00
Feroculus_feroculus	3	6.29	0.69	0.00
Fossa_fossana	0	0.02	0.56	0.00
Fukomys_anselli	1.23	2.02	0.67	0.00
Fukomys_bocagei	0.01	0.1	0.73	0.00
Fukomys_damarensis	0.03	0.07	0.66	0.00
Fukomys_darlingi	0.37	0.41	0.01	0.98
Fukomys_kafuensis	0.01	0.02	0.31	0.05
Fukomys_mechowi	0.05	0.12	0.69	0.00
Fukomys_ochraceocinereus	0.01	0.01	0.41	0.01
Fukomys_zechi	0.11	0.3	0.78	0.00
Funambulus_layardi	2.23	6.5	0.73	0.00
Funambulus_palmarum	2.31	5.64	0.79	0.00
Funambulus_pennantii	2.38	4.55	0.70	0.00
Funambulus_sublineatus	1.5	3.86	0.68	0.00
Funambulus_tristriatus	2.45	5.62	0.70	0.00
Funisciurus_anerythrus	0.21	0.24	0.08	0.65
Funisciurus_carruthersi	0.1	0.22	0.50	0.00
Funisciurus_congicus	0.03	0.11	0.75	0.00
Funisciurus_isabella	0.07	0.14	0.67	0.00

Funisciurus_lemniscatus	0.16	0.33	0.71	0.00
Funisciurus_leucogenys	2.6	2.4	-0.29	0.07
Funisciurus_pyrropus	0.34	0.43	0.44	0.01
Furipterus_horrens	0.58	1.09	0.75	0.00
Galago_gallarum	0.01	0.02	0.59	0.00
Galago_matschiei	0.19	0.4	0.69	0.00
Galago_moholi	0.12	0.27	0.73	0.00
Galago_senegalensis	0.06	0.14	0.70	0.00
Galagoides_cocos	1.34	2.28	0.57	0.00
Galagoides_demidovii	0.3	0.38	0.33	0.04
Galagoides_granti	0.05	0.13	0.67	0.00
Galagoides_orinus	0.08	0.11	0.27	0.10
Galagoides_rondoensis	0.31	0.78	0.43	0.01
Galagoides_thomasi	0.28	0.35	0.31	0.05
Galagoides_zanzibaricus	0.73	1.31	0.61	0.00
Galea_flavidens	0.22	0.56	0.71	0.00
Galea_musteloides	0.25	0.61	0.82	0.00
Galea_spixii	0.32	0.77	0.75	0.00
Galemys_pyrenaicus	5.45	9.49	0.84	0.00
Galeopterus_variegatus	0.75	1.92	0.77	0.00
Galictis_cuja	0.75	1.53	0.79	0.00
Galictis_vittata	0.38	0.73	0.70	0.00
Galidia_elegans	0	0.02	0.54	0.00
Galidictis_fasciata	0	0.03	0.52	0.00
Galidictis_grandidieri	0	0	-0.12	0.55
Gazella_bennettii	1.43	2.93	0.68	0.00
Gazella_cuvieri	0.49	1.37	0.72	0.00
Gazella_dorcas	0.19	0.3	0.67	0.00
Gazella_gazella	3.85	7.68	0.86	0.00
Gazella_leptoceros	0.47	0.47	0.09	0.61
Gazella_spekei	0	0.01	0.58	0.00
Gazella_subgutturosa	0.74	1.42	0.75	0.00
Genetta_abyssinica	0.06	0.17	0.73	0.00
Genetta_angolensis	0.06	0.13	0.69	0.00
Genetta_bourloni	0.02	0.09	0.69	0.00
Genetta_cristata	7.28	5.4	-0.52	0.00
Genetta_genetta	0.61	1.11	0.78	0.00
Genetta_johnstoni	0.15	0.38	0.70	0.00
Genetta_maculata	0.15	0.23	0.59	0.00
Genetta_pardina	0.11	0.32	0.70	0.00
Genetta_servalina	0.06	0.12	0.67	0.00
Genetta_thierryi	0.16	0.36	0.73	0.00
Genetta_tigrina	1.31	1.92	0.64	0.00
Genetta_victoriae	0.01	0.02	0.34	0.03
Geocapromys_brownii	4.28	6.06	0.43	0.01
Geocapromys_ingrahami	0	0	-0.12	0.55

Geogale_aurita	0	0	-0.06	0.76
Geoxus_valdivianus	0.32	0.9	0.75	0.00
Gerbilliscus_afra	1.25	1.68	0.61	0.00
Gerbilliscus_boehmi	0.06	0.15	0.68	0.00
Gerbilliscus_brantsii	0.46	0.76	0.72	0.00
Gerbilliscus_gambiana	0.08	0.25	0.79	0.00
Gerbilliscus_guineae	0.06	0.16	0.69	0.00
Gerbilliscus_inclusus	0.05	0.1	0.43	0.01
Gerbilliscus_kempi	0.07	0.15	0.69	0.00
Gerbilliscus_leucogaster	0.2	0.36	0.73	0.00
Gerbilliscus_nigricaudus	0.07	0.15	0.58	0.00
Gerbilliscus_phillipsi	0	0.02	0.56	0.00
Gerbilliscus_robustus	0.04	0.11	0.72	0.00
Gerbilliscus_validus	0.04	0.12	0.76	0.00
Gerbillurus_paeba	0.14	0.25	0.63	0.00
Gerbillurus_setzeri	0.07	0.11	0.52	0.00
Gerbillurus_tytonis	0.07	0.11	0.53	0.00
Gerbillurus_vallinus	0.04	0.08	0.48	0.00
Gerbillus_amoenus	0.58	0.72	0.50	0.00
Gerbillus_andersoni	4.96	9.68	0.90	0.00
Gerbillus_aquilus	0.16	0.39	0.73	0.00
Gerbillus_campestris	1.08	2.41	0.77	0.00
Gerbillus_cheesmani	1.23	2.5	0.77	0.00
Gerbillus_dasyurus	2.09	3.81	0.79	0.00
Gerbillus_dunni	0.02	0.04	0.60	0.00
Gerbillus_famulus	0.86	2.24	0.72	0.00
Gerbillus_floweri	3.39	7.04	0.88	0.00
Gerbillus_gerbillus	0.28	0.48	0.70	0.00
Gerbillus_gleadowi	0.85	2.17	0.70	0.00
Gerbillus_harwoodi	0.2	0.46	0.66	0.00
Gerbillus_henleyi	0.78	1.69	0.81	0.00
Gerbillus_hesperinus	1.43	3.12	0.75	0.00
Gerbillus_hoogstraali	1.76	5.08	0.84	0.00
Gerbillus_juliani	0	0	0.15	0.35
Gerbillus_latastei	1.26	2.96	0.80	0.00
Gerbillus_mackillingini	0.02	0.08	0.71	0.00
Gerbillus_maghrebi	2.12	4.6	0.80	0.00
Gerbillus_mesopotamiae	4.55	6.56	0.67	0.00
Gerbillus_nanus	0.83	1.55	0.72	0.00
Gerbillus_nigeriae	0.07	0.17	0.73	0.00
Gerbillus_perpallidus	3.28	8.17	0.91	0.00
Gerbillus_poecilops	4.03	8.06	0.82	0.00
Gerbillus_pulvinatus	0	0	0.12	0.54
Gerbillus_pusillus	0.03	0.08	0.69	0.00
Gerbillus_pyramidum	0.38	0.81	0.81	0.00
Gerbillus_rosalinda	0.02	0.14	0.77	0.00

Gerbillus_rupicola	0.01	0.05	0.63	0.00
Gerbillus_simoni	1.5	3.6	0.83	0.00
Gerbillus_tarabuli	0.15	0.22	0.57	0.00
Gerbillus_watersi	0.47	1.23	0.77	0.00
Giraffa_camelopardalis	0.14	0.29	0.70	0.00
Glaucomys_sabrinus	1.76	1.75	-0.10	0.57
Glaucomys_volans	9.09	10.5	0.49	0.00
Glauconycteris_alboguttata	0.02	0.04	0.61	0.00
Glauconycteris_argentata	0.06	0.13	0.71	0.00
Glauconycteris_beatrix	0.2	0.3	0.59	0.00
Glauconycteris_poensis	0.88	0.97	0.08	0.65
Glauconycteris_superba	0.81	1.68	0.27	0.10
Glauconycteris_variegata	0.08	0.17	0.70	0.00
Glironia_venusta	0.11	0.21	0.72	0.00
Glirulus_japonicus	10.15	11.86	0.51	0.00
Glis_glis	5.44	8.8	0.80	0.00
Glischropus_tylopus	0.74	2.12	0.78	0.00
Glossophaga_commissarisi	0.48	0.88	0.66	0.00
Glossophaga_leachii	2.19	3.86	0.68	0.00
Glossophaga_morenoi	3.21	4.62	0.62	0.00
Glossophaga_soricina	0.66	1.26	0.73	0.00
Glyphonycteris_daviesi	0.07	0.16	0.76	0.00
Glyphonycteris_sylvestris	0.55	0.96	0.75	0.00
Golunda_ellioti	2.59	5.32	0.74	0.00
Gorilla_beringei	0	0	-0.07	0.69
Gorilla_gorilla	0.09	0.2	0.68	0.00
Gracilinanus_aceramarcae	0.01	0.06	0.48	0.00
Gracilinanus_agilis	0.57	1.23	0.76	0.00
Gracilinanus_dryas	3.22	4.32	0.57	0.00
Gracilinanus_marica	6.08	9.11	0.72	0.00
Gracilinanus_microtarsus	2.7	5.14	0.77	0.00
Grammomys_cometes	0.4	0.9	0.68	0.00
Grammomys_dolichurus	0.16	0.33	0.74	0.00
Grammomys_dryas	0.01	0.12	0.61	0.00
Grammomys_gigas	0.05	0.16	0.24	0.14
Grammomys_ibeanus	0.13	0.32	0.68	0.00
Grammomys_kuru	0.56	0.65	0.20	0.22
Grammomys_macmillani	0.02	0.05	0.52	0.00
Grammomys_minnae	0.01	0.06	0.60	0.00
Graomys_domorum	0.36	0.87	0.71	0.00
Graomys_griseoflavus	0.22	0.55	0.80	0.00
Graphiurus_christyi	0.02	0.03	0.35	0.03
Graphiurus_kelleni	0.12	0.25	0.67	0.00
Graphiurus_lorraineus	0.14	0.22	0.65	0.00
Graphiurus_microtis	0.22	0.42	0.73	0.00
Graphiurus_murinus	0.59	1.09	0.70	0.00

Graphiurus_nagtglasii	1.31	1.4	0.00	1.00
Graphiurus_ocularis	0.68	0.94	0.59	0.00
Graphiurus_platyops	0.97	1.87	0.72	0.00
Graphiurus_rupicola	0.08	0.08	-0.08	0.65
Gulo_gulo	0.38	0.49	0.59	0.00
Gymnobelideus_leadbeateri	0.18	0.27	0.18	0.26
Gymnuromys_roberti	0	0.02	0.49	0.00
Habromys_chinanteco	0	0	-0.12	0.55
Habromys_delicatulus	58.89	60.58	0.30	0.06
Habromys_ixtlani	0.01	0	-0.23	0.21
Habromys_lepturus	0	0.82	0.55	0.00
Habromys_lophurus	2.3	6.17	0.82	0.00
Habromys_schmidlyi	7.27	8.8	0.32	0.04
Habromys_simulatus	2.47	4.09	0.45	0.00
Hadromys_humei	2.3	3.18	0.30	0.07
Haeromys_minahassae	0.28	0.92	0.53	0.00
Haeromys_pusillus	0.11	0.18	0.38	0.02
Handleyomys_alfaroi	1.52	2.89	0.70	0.00
Handleyomys_chapmani	2.48	4.44	0.58	0.00
Handleyomys_fuscatus	1.31	1.84	0.45	0.00
Handleyomys_intectus	7.15	8.39	0.44	0.01
Handleyomys_melanotis	1.47	2.25	0.53	0.00
Handleyomys_rhabdops	1.73	4.52	0.75	0.00
Handleyomys_rostratus	1.74	3.06	0.64	0.00
Handleyomys_saturatior	1.37	3.52	0.74	0.00
Hapalemur_alaotrensis	0	0	-0.12	0.55
Hapalemur_aureus	0	0	-0.21	0.27
Hapalemur_griseus	0	0.02	0.44	0.01
Hapalemur_meridionalis	0.01	0.08	0.32	0.05
Hapalemur_occidentalis	0	0.01	0.12	0.45
Hapalomys_delacouri	0.39	1.79	0.81	0.00
Hapalomys_longicaudatus	2.86	5.36	0.79	0.00
Haplonycteris_fischeri	0.94	1.88	0.62	0.00
Harpiocephalus_harpia	2.11	4.46	0.76	0.00
Harpyionycteris_celebensis	0.21	0.63	0.61	0.00
Harpyionycteris_whiteheadi	0.48	1.11	0.52	0.00
Heimyscus_fumosus	0.03	0.08	0.69	0.00
Helarctos_malayanus	0.62	1.76	0.80	0.00
Heliosciurus_gambianus	0.06	0.14	0.74	0.00
Heliosciurus_mutabilis	0.04	0.11	0.64	0.00
Heliosciurus_rufobrachium	0.46	0.57	0.32	0.04
Heliosciurus_ruwenzorii	0.08	0.19	0.52	0.00
Helogale_hirtula	0	0.01	0.44	0.01
Helogale_parvula	0.07	0.17	0.70	0.00
Hemibelideus_lemuroides	0.04	0.12	0.31	0.05
Hemicentetes_nigriceps	0.02	0.03	0.21	0.19

Hemicentetes_semispinosus	0.01	0.03	0.51	0.00
Hemiechinus_auritus	0.83	1.47	0.79	0.00
Hemiechinus_collaris	2.26	4	0.65	0.00
Hemigalus_derbyanus	0.67	1.73	0.75	0.00
Hemitragus_jemlahicus	0.02	0.15	0.72	0.00
Herpestes_brachyurus	0.64	1.53	0.74	0.00
Herpestes_edwardsii	2.5	5.02	0.76	0.00
Herpestes_flavescens	0.02	0.07	0.68	0.00
Herpestes_fuscus	2.41	5.74	0.77	0.00
Herpestes_ichneumon	0.39	0.69	0.75	0.00
Herpestes_javanicus	1.57	3.32	0.80	0.00
Herpestes_naso	0.13	0.15	0.09	0.61
Herpestes_ochraceus	0	0.01	0.64	0.00
Herpestes_pulverulentus	0.42	0.63	0.66	0.00
Herpestes_sanguineus	0.12	0.24	0.72	0.00
Herpestes_smithii	2.46	5.52	0.72	0.00
Herpestes_urva	0.98	2.98	0.85	0.00
Herpestes_vitticollis	2.45	6.43	0.80	0.00
Hesperoptenus_blanfordi	1.5	4.38	0.87	0.00
Hesperoptenus_tickelli	0.98	2.64	0.78	0.00
Hesperoptenus_tomesi	1.48	4.8	0.90	0.00
Heterohyrax_brucei	0.07	0.17	0.71	0.00
Heteromys_anomalus	3.41	5	0.64	0.00
Heteromys_australis	1.63	2.23	0.46	0.00
Heteromys_desmarestianus	0.89	2.2	0.69	0.00
Heteromys_gaumeri	0.85	1.57	0.61	0.00
Heteromys_nelsoni	0.04	2.54	0.76	0.00
Heteromys_oasicus	7.73	10.75	0.50	0.00
Heteromys_oresterus	0.72	2.59	0.56	0.00
Heteromys_teleus	1.34	4.03	0.81	0.00
Hippocamelus_antisensis	0.13	0.37	0.72	0.00
Hippocamelus_bisulcus	0.01	0.06	0.73	0.00
Hippopotamus_amphibius	0.12	0.16	0.37	0.02
Hipposideros_abae	0.19	0.37	0.71	0.00
Hipposideros_armiger	1.04	3.13	0.85	0.00
Hipposideros_ater	0.82	1.9	0.75	0.00
Hipposideros_beatus	0.25	0.43	0.67	0.00
Hipposideros_bicolor	1.03	2.4	0.77	0.00
Hipposideros_caffer	0.32	0.67	0.76	0.00
Hipposideros_calcaratus	0.07	0.13	0.45	0.00
Hipposideros_cervinus	0.49	1.21	0.74	0.00
Hipposideros_cineraceus	1.04	2.57	0.83	0.00
Hipposideros_commersoni	0.02	0.03	0.47	0.00
Hipposideros_curtus	0.23	0.42	0.61	0.00
Hipposideros_cyclops	0.56	0.67	0.34	0.03
Hipposideros_demissus	0	0	-0.12	0.55

Hipposideros_diadema	0.8	1.98	0.78	0.00
Hipposideros_doriae	0.62	1.5	0.75	0.00
Hipposideros_durgadasi	0.63	1.16	0.30	0.07
Hipposideros_dyacorum	0.33	0.88	0.78	0.00
Hipposideros_fuliginosus	0.08	0.18	0.70	0.00
Hipposideros_fulvus	2.39	4.98	0.71	0.00
Hipposideros_galeritus	1.18	2.76	0.76	0.00
Hipposideros_gigas	0.23	0.36	0.70	0.00
Hipposideros_halophyllus	3.76	7.11	0.58	0.00
Hipposideros_hypophyllus	10.65	24.51	0.96	0.00
Hipposideros_inornatus	0.13	0.22	0.46	0.00
Hipposideros_jonesi	0.11	0.29	0.71	0.00
Hipposideros_khaokhouayensis	0.04	0.38	0.66	0.00
Hipposideros_lamottei	0	0.29	0.45	0.01
Hipposideros_lankadiva	2.25	5.64	0.72	0.00
Hipposideros_larvatus	0.85	2.33	0.80	0.00
Hipposideros_lekaguli	1.09	3.32	0.76	0.00
Hipposideros_lylei	0.66	2.11	0.80	0.00
Hipposideros_madurae	4.39	9.74	0.73	0.00
Hipposideros_maggietaylorae	0.06	0.09	0.38	0.02
Hipposideros_marisae	0.03	0.12	0.56	0.00
Hipposideros_megalotis	0.09	0.2	0.69	0.00
Hipposideros_obscurus	1.27	2.44	0.65	0.00
Hipposideros_orbiculus	4.8	20.49	0.93	0.00
Hipposideros_papua	0.07	0.17	0.43	0.01
Hipposideros_pelingensis	0.21	0.61	0.61	0.00
Hipposideros_pomona	0.65	2.1	0.81	0.00
Hipposideros_pratti	1.05	3.44	0.85	0.00
Hipposideros_pygmaeus	1.07	2.14	0.59	0.00
Hipposideros_ridleyi	1.97	5.56	0.90	0.00
Hipposideros_rotalis	0.02	0.24	0.61	0.00
Hipposideros_ruber	0.07	0.16	0.74	0.00
Hipposideros_scutinares	0.01	0.17	0.72	0.00
Hipposideros_sorenseni	1.84	4.63	0.43	0.01
Hipposideros_speoris	2.75	6.46	0.80	0.00
Hipposideros_stenotis	0.03	0.06	0.61	0.00
Hipposideros_sumbae	0.14	0.51	0.59	0.00
Hipposideros_thomensis	0.8	1.99	0.60	0.00
Hipposideros_turpis	1.65	5.54	0.81	0.00
Hipposideros_vittatus	0.08	0.13	0.51	0.00
Hipposideros_wollastoni	0.13	0.32	0.31	0.05
Hippotragus_equinus	0.05	0.13	0.71	0.00
Hippotragus_niger	0.13	0.28	0.69	0.00
Histiotus_laephotis	0.48	1.1	0.77	0.00
Histiotus_macrotus	0.37	0.88	0.80	0.00
Histiotus_magellanicus	0.17	0.45	0.71	0.00

Histiotus_montanus	0.7	1.31	0.74	0.00
Hodomys_alleni	2.75	3.95	0.62	0.00
Holochilus_brasiliensis	1.26	2.55	0.81	0.00
Holochilus_chacarius	0.38	0.84	0.75	0.00
Holochilus_sciureus	0.25	0.5	0.76	0.00
Hoolock_hoolock	0.67	1.19	0.57	0.00
Hoolock_leuconedys	0.07	0.48	0.80	0.00
Hoplomys_gymnurus	0.32	0.87	0.68	0.00
Hyaena_brunnea	0.39	0.63	0.73	0.00
Hyaena_hyaena	0.8	1.53	0.75	0.00
Hybomys_badius	0.04	0.14	0.32	0.04
Hybomys_basilii	1.28	0.3	-0.46	0.00
Hybomys_lunaris	0.01	0	-0.20	0.24
Hybomys_planifrons	0.02	0.08	0.69	0.00
Hybomys_trivirgatus	1.21	1.62	0.37	0.02
Hybomys_univittatus	0.07	0.14	0.72	0.00
Hydrochoerus_hydrochaeris	0.41	0.86	0.79	0.00
Hydromys_chrysogaster	0.29	0.41	0.60	0.00
Hydropotes_inermis	7.17	17.22	0.90	0.00
Hyemoschus_aquaticus	0.07	0.18	0.72	0.00
Hyladelphys_kalinowskii	0.03	0.08	0.76	0.00
Hylaeamys_laticeps	2.92	5.45	0.74	0.00
Hylaeamys_megacephalus	0.26	0.6	0.78	0.00
Hylaeamys_oniscus	10.33	14.25	0.47	0.00
Hylaeamys_perenensis	0.02	0.05	0.66	0.00
Hylaeamys_yunganus	0.07	0.15	0.73	0.00
Hylobates_agilis	0.75	1.81	0.59	0.00
Hylobates_albibarbis	0.06	0.08	0.14	0.38
Hylobates_klossii	0	0	-0.16	0.33
Hylobates_lar	1.7	4.18	0.81	0.00
Hylobates_moloch	3.32	7.17	0.63	0.00
Hylobates_muelleri	0.3	0.76	0.73	0.00
Hylobates_pileatus	0.45	1.53	0.68	0.00
Hylochoerus_meinertzhageni	0.08	0.21	0.68	0.00
Hylomys_parvus	0.01	0.08	0.31	0.05
Hylomys_suillus	0.91	2.52	0.80	0.00
Hylomyscus_aeta	0.05	0.1	0.75	0.00
Hylomyscus_alleni	0.17	0.41	0.74	0.00
Hylomyscus_baeri	0.63	1.32	0.56	0.00
Hylomyscus_carillus	0.02	0.13	0.69	0.00
Hylomyscus_denniae	0.13	0.3	0.68	0.00
Hylomyscus_grandis	0	0	-0.12	0.55
Hylomyscus_parvus	0.03	0.06	0.61	0.00
Hylomyscus_stella	0.39	0.35	-0.26	0.11
Hylonycteris_underwoodi	1.84	3.14	0.62	0.00
Hylopetes_alboniger	0.28	1.37	0.82	0.00

Hylopetes_nigripes	0.06	0.25	0.57	0.00
Hylopetes_phayrei	0.32	1.13	0.75	0.00
Hylopetes_sipora	0	0.05	0.23	0.21
Hylopetes_spadiceus	1.02	2.7	0.79	0.00
Hyomys_goliath	0.04	0.07	0.36	0.02
Hyosciurus_heinrichi	0.01	0.04	0.27	0.10
Hyosciurus_ileile	0	0.01	0.20	0.21
Hyperacrius_fertilis	0.32	0.88	0.66	0.00
Hyperacrius_wynnei	2.55	4.85	0.76	0.00
Hypogeomys_antimena	0	0	-0.12	0.55
Hypsignathus_monstrosus	0.15	0.36	0.78	0.00
Hypsiprymnodon_moschatus	0.19	0.44	0.41	0.01
Hystrix_africaeaustralis	0.22	0.38	0.70	0.00
Hystrix_brachyura	0.88	2.67	0.84	0.00
Hystrix_crassispinis	0.24	0.57	0.71	0.00
Hystrix_cristata	0.6	1.06	0.78	0.00
Hystrix_indica	2.03	3.87	0.77	0.00
Hystrix_javanica	4.11	8.35	0.73	0.00
Hystrix_pumila	0.05	0.25	0.58	0.00
Hystrix_sumatrae	0.75	1.42	0.49	0.00
Ia_io	0.41	1.54	0.80	0.00
Ichneumia_albicauda	0.22	0.45	0.78	0.00
Ichthyomys_hydrobates	2.08	3.17	0.64	0.00
Ichthyomys_pittieri	15.86	18.23	0.59	0.00
Ictonyx_libyca	0.41	0.77	0.72	0.00
Ictonyx_striatus	0.14	0.26	0.71	0.00
Idionycteris_phyllotis	1.96	2.88	0.66	0.00
Idiurus_macrotis	0.31	0.56	0.66	0.00
Idiurus_zenkeri	0.09	0.19	0.70	0.00
Indri_indri	0	0	0.23	0.17
Iomys_horsfieldii	1.27	3.01	0.77	0.00
Iomys_sipora	0.01	0.02	-0.05	0.81
Irenomys_tarsalis	0.24	0.57	0.70	0.00
Isoodon_auratus	0.16	0.11	-0.40	0.01
Isoodon_macrourus	0.34	0.5	0.64	0.00
Isoodon_obesulus	0.87	1.23	0.59	0.00
Isothrix_bistriata	0.04	0.13	0.76	0.00
Isothrix_negrensis	0.02	0.05	0.48	0.00
Isothrix_pagurus	0.1	0.25	0.76	0.00
Isothrix_sinnamariensis	0	0	-0.12	0.55
Isthmomys_flavidus	0.07	0.24	0.57	0.00
Isthmomys_pirrensis	0	0	0.07	0.69
Jaculus_blanfordi	0.5	1.13	0.70	0.00
Jaculus_jaculus	0.88	1.6	0.71	0.00
Jaculus_orientalis	2.2	4.97	0.84	0.00
Juliomys_pictipes	2.81	5.44	0.79	0.00

Juliomys_rimofrons	1.24	1.8	0.33	0.04
Kadarsanomys_sodyi	1.91	4.84	0.44	0.01
Kannabateomys_amblyonyx	2.33	4.68	0.82	0.00
Kerivoula_africana	0.11	0.16	0.45	0.00
Kerivoula_argentata	0.07	0.12	0.46	0.00
Kerivoula_flora	0.66	1.57	0.65	0.00
Kerivoula_hardwickii	0.78	2.12	0.80	0.00
Kerivoula_intermedia	0.62	1.8	0.83	0.00
Kerivoula_kachinensis	0.26	1.12	0.74	0.00
Kerivoula_lanosa	0.13	0.25	0.73	0.00
Kerivoula_lenis	2.69	7.95	0.93	0.00
Kerivoula_minuta	1	3.15	0.79	0.00
Kerivoula_muscina	0.05	0.08	0.32	0.04
Kerivoula_papillosa	1.19	2.87	0.78	0.00
Kerivoula_pellucida	1.17	2.64	0.75	0.00
Kerivoula_phalaena	0.07	0.21	0.70	0.00
Kerivoula_picta	1.15	3	0.80	0.00
Kerivoula_smithii	0.03	0.06	0.65	0.00
Kerivoula_titania	0.22	0.89	0.73	0.00
Kerivoula_whiteheadi	0.92	1.85	0.62	0.00
Kerodon_rupestris	0.46	1.15	0.73	0.00
Kobus_ellipsiprymnus	0.08	0.18	0.70	0.00
Kobus_kob	0.07	0.15	0.73	0.00
Kobus_leche	0.01	0.04	0.69	0.00
Kobus_megaceros	0	0.03	0.59	0.00
Kobus_vardonii	0	0.01	0.63	0.00
Komodomys_rintjanus	0.06	0.22	0.54	0.00
Kunsia_fronto	1.64	2.97	0.75	0.00
Kunsia_tomentosus	0.05	0.16	0.73	0.00
Laephotis_botswanae	0.24	0.51	0.63	0.00
Laephotis_namibensis	0.02	0.03	0.33	0.04
Laephotis_wintoni	0.1	0.2	0.69	0.00
Lagidium_peruanum	0.21	0.57	0.71	0.00
Lagidium_viscacia	0.14	0.4	0.82	0.00
Lagorchestes_conspicillatus	0.04	0.08	0.61	0.00
Lagorchestes_hirsutus	0	0	-0.12	0.55
Lagostomus_maximus	0.54	1.17	0.78	0.00
Lagostrophus_fasciatus	0	0	-0.12	0.55
Lagothrix_cana	0.02	0.1	0.78	0.00
Lagothrix_lagotricha	0.09	0.13	0.58	0.00
Lagothrix_lugens	1.02	1.9	0.49	0.00
Lagothrix_poeppigii	0.04	0.11	0.69	0.00
Lagurus_lagurus	1.13	1.33	0.18	0.26
Lama_guanicoe	0.22	0.48	0.70	0.00
Lamottemys_okuensis	0	0	-0.12	0.55
Lampronycteris_brachyotis	0.39	0.73	0.72	0.00

Laonastes_aenigmamus	0	0.01	0.17	0.33
Lariscus_hosei	0.09	0.11	0.13	0.41
Lariscus_insignis	1.21	2.78	0.78	0.00
Lariscus_obscurus	0	0	-0.15	0.36
Lasionycteris_noctivagans	4.2	4.74	0.34	0.03
Lasiopodomys_brandtii	0.09	0.33	0.84	0.00
Lasiopodomys_fuscus	0	0.01	0.44	0.01
Lasiopodomys_mandarinus	2.92	6.76	0.83	0.00
Lasiorhinus_krefftii	0.04	0	-0.25	0.19
Lasiorhinus_latifrons	0	0.01	0.30	0.06
Lasiurus_atratus	0.09	0.21	0.71	0.00
Lasiurus_blossevillii	0.88	1.41	0.72	0.00
Lasiurus_borealis	7.53	8.71	0.46	0.00
Lasiurus_cinereus	2.59	3.18	0.53	0.00
Lasiurus_degelidus	7.39	9.9	0.50	0.00
Lasiurus_ega	0.61	1.19	0.74	0.00
Lasiurus_insularis	0.93	2.54	0.60	0.00
Lasiurus_intermedius	4.66	6.24	0.66	0.00
Lasiurus_minor	4.53	6.02	0.55	0.00
Lasiurus_pfeifferi	0.93	2.54	0.60	0.00
Lasiurus_seminolus	8.02	10.01	0.60	0.00
Lasiurus_varius	0.43	1.03	0.78	0.00
Lasiurus_xanthinus	2.26	3.13	0.65	0.00
Latidens_salimalii	2.63	5.5	0.74	0.00
Lavia_frons	0.07	0.15	0.72	0.00
Leggadina_forresti	0.01	0.02	0.30	0.06
Leggadina_lakedownensis	0.03	0.07	0.59	0.00
Lemmiscus_curtatus	1.29	1.46	0.14	0.38
Lemmus_amurensis	0.11	0.11	0.07	0.69
Lemmus_lemmus	1.83	2.53	0.39	0.01
Lemmus_sibiricus	0.15	0.28	0.56	0.00
Lemmus_trimucronatus	0.04	0.05	0.29	0.07
Lemniscomys_barbarus	2.49	5.92	0.82	0.00
Lemniscomys_bellieri	0.07	0.24	0.46	0.00
Lemniscomys_griselda	0	0.06	0.74	0.00
Lemniscomys_linulus	0.02	0.09	0.54	0.00
Lemniscomys_macculus	0.02	0.06	0.66	0.00
Lemniscomys_mittendorfi	0	0	-0.12	0.55
Lemniscomys_rosalia	0.26	0.51	0.71	0.00
Lemniscomys_striatus	0.24	0.4	0.70	0.00
Lemniscomys_zebra	0.07	0.16	0.67	0.00
Lemur_catta	0	0.01	0.27	0.10
Lenomys_meyeri	0.27	0.77	0.57	0.00
Lenothrix_canus	1.68	4.89	0.89	0.00
Lenoxus_apicalis	0.01	0.04	0.26	0.11
Leontopithecus_caissara	0	0.05	0.35	0.04

Leontopithecus_chrysomelas	0.94	1.46	0.54	0.00
Leontopithecus_chrysopygus	1.71	4.03	0.76	0.00
Leontopithecus_rosalia	8.9	15.85	0.79	0.00
Leopardus_colocolo	0.33	0.75	0.77	0.00
Leopardus_geoffroyi	0.45	0.91	0.76	0.00
Leopardus_guigna	0.34	0.91	0.75	0.00
Leopardus_jacobita	0.1	0.31	0.70	0.00
Leopardus_pardalis	0.51	1.04	0.75	0.00
Leopardus_tigrinus	0.51	1.02	0.76	0.00
Leopardus_wiedii	0.55	1.06	0.73	0.00
Leopoldamys_ciliatus	0.37	1.23	0.68	0.00
Leopoldamys_edwardsi	0.74	2.43	0.80	0.00
Leopoldamys_milleti	0.21	2.46	0.82	0.00
Leopoldamys_sabanus	0.81	2.17	0.77	0.00
Leopoldamys_siporanus	0	0	-0.15	0.36
Lepilemur_ankaranensis	0	0.01	0.19	0.28
Lepilemur_edwardsi	0.06	0.11	0.18	0.26
Lepilemur_septentrionalis	0	0	-0.12	0.55
Leporillus_apicalis	0.01	0.01	-0.02	0.93
Leporillus_conditor	0	0	0.14	0.45
Leptailurus_serval	0.16	0.3	0.71	0.00
Leptomys_elegans	0.14	0.26	0.35	0.03
Leptomys_ernstmayri	0.04	0.05	0.00	1.00
Leptomys_signatus	0	0	-0.12	0.55
Leptonycteris_curasoae	2.04	3.15	0.60	0.00
Leptonycteris_nivalis	2.52	3.82	0.66	0.00
Leptonycteris_yerbabuenae	2.15	3.36	0.68	0.00
Lepus_alleni	1.52	2.22	0.59	0.00
Lepus_americanus	1.66	1.65	-0.04	0.83
Lepus_arcticus	0.01	0.02	0.35	0.03
Lepus_brachyurus	14.18	15.86	0.50	0.00
Lepus_californicus	2.55	3.11	0.54	0.00
Lepus_callotis	3.84	5.68	0.66	0.00
Lepus_capensis	0.76	1.46	0.77	0.00
Lepus_castroviejoi	0.84	2.96	0.59	0.00
Lepus_comus	0.45	1.93	0.83	0.00
Lepus_coreanus	6	9.12	0.82	0.00
Lepus_corsicanus	10.57	14.7	0.79	0.00
Lepus_europaeus	3.07	4.43	0.70	0.00
Lepus_flavigularis	0.31	0.57	0.27	0.10
Lepus_granatensis	4.89	8.32	0.83	0.00
Lepus_habessinicus	0.01	0.05	0.65	0.00
Lepus_hainanus	1.34	5.42	0.88	0.00
Lepus_insularis	0	0	-0.12	0.55
Lepus_mandshuricus	0.87	2.18	0.72	0.00
Lepus_microtis	0.16	0.3	0.72	0.00

Lepus_nigricollis	2.68	5.4	0.75	0.00
Lepus_oiostolus	0.02	0.13	0.82	0.00
Lepus_othus	0.07	0.07	0.08	0.65
Lepus_peguensis	0.29	1.26	0.76	0.00
Lepus_saxatilis	0.94	1.5	0.70	0.00
Lepus_sinensis	2.46	6.34	0.86	0.00
Lepus_starcki	0.22	0.55	0.71	0.00
Lepus_tibetanus	0.16	0.59	0.83	0.00
Lepus_timidus	1	1.34	0.51	0.00
Lepus_tolai	0.86	2.09	0.79	0.00
Lepus_townsendii	2.54	2.85	0.14	0.38
Lepus_yarkandensis	0.09	0.41	0.76	0.00
Lestodelphys_halli	0.12	0.25	0.60	0.00
Lestoros_inca	0.01	0.06	0.53	0.00
Liberiictis_kuhni	0.02	0.06	0.43	0.01
Lichonycteris_obscura	0.41	0.78	0.70	0.00
Limnogale_mergulus	0.12	0.23	0.62	0.00
Limnomys_bryophilus	0	0.16	0.29	0.07
Limnomys_sibuanus	0	0.23	0.23	0.15
Liomys_adspersus	1.38	3.24	0.76	0.00
Liomys_irroratus	3.41	5.09	0.67	0.00
Liomys_pictus	1.65	2.52	0.57	0.00
Liomys_salvini	1.89	4.22	0.73	0.00
Liomys_spectabilis	1.8	3.04	0.67	0.00
Lionycteris_spurrelli	0.33	0.56	0.65	0.00
Lissonycteris_angolensis	0.07	0.15	0.72	0.00
Litocranius_walleri	0.01	0.02	0.53	0.00
Lonchophylla_concava	1.39	3.31	0.78	0.00
Lonchophylla_dekeyseri	0.07	0.22	0.70	0.00
Lonchophylla_handleyi	0.04	0.18	0.70	0.00
Lonchophylla_hesperia	0.56	1.21	0.77	0.00
Lonchophylla_mordax	1.22	2.41	0.73	0.00
Lonchophylla_robusta	1.56	2.58	0.66	0.00
Lonchophylla_thomasi	0.31	0.55	0.67	0.00
Lonchorhina_aurita	0.58	1.11	0.72	0.00
Lonchorhina_fernandezi	0	0.01	0.40	0.01
Lonchorhina_marinkellei	0	0.01	0.48	0.00
Lonchorhina_orinocensis	0.05	0.19	0.74	0.00
Lonchothrix_emiliae	0.04	0.13	0.76	0.00
Lontra_canadensis	2.59	2.88	0.29	0.07
Lontra_felina	0.07	0.13	0.44	0.01
Lontra_provocax	0.15	0.3	0.65	0.00
Lophiomys_imhausi	0.14	0.31	0.69	0.00
Lophocebus_albigena	0.06	0.12	0.59	0.00
Lophocebus_aterrimus	0	0	0.58	0.00
Lophostoma_brasiliense	0.4	0.75	0.72	0.00

Lophostoma_carrikeri	0.07	0.18	0.74	0.00
Lophostoma_evotis	1.27	2.32	0.71	0.00
Lophostoma_schulzi	0.06	0.13	0.70	0.00
Lophostoma_silvicolum	0.43	0.83	0.72	0.00
Lophuromys_brevicaudus	0.02	0.05	0.51	0.00
Lophuromys_chrysopus	0.01	0.06	0.69	0.00
Lophuromys_dieterleni	0	0	-0.12	0.55
Lophuromys_eisentrauti	0	0	-0.12	0.55
Lophuromys_flavopunctatus	0.02	0.06	0.70	0.00
Lophuromys_huttereri	0	0	0.03	0.92
Lophuromys_luteogaster	0	0	-0.24	0.20
Lophuromys_medicaudatus	0.37	0.75	0.39	0.01
Lophuromys_melanonyx	0.02	0.03	0.35	0.03
Lophuromys_nudicaudus	0.14	0.23	0.55	0.00
Lophuromys_rahmi	0	0.02	0.35	0.04
Lophuromys_roseveari	0.21	0.22	0.32	0.04
Lophuromys_sikapusi	0.41	0.51	0.35	0.03
Lophuromys_woosnami	0.18	0.33	0.38	0.02
Lorentzimys_nouhuysi	0.04	0.08	0.35	0.03
Loris_lydekkerianus	3.71	8.61	0.85	0.00
Loris_tardigradus	3.78	8.91	0.74	0.00
Loxodonta_africana	0.02	0.05	0.64	0.00
Loxodontomys_micropus	0.4	0.97	0.80	0.00
Loxodontomys_pikumche	2.18	5	0.87	0.00
Lundomys_molitor	0.41	0.79	0.61	0.00
Lutra_lutra	2.22	3.61	0.76	0.00
Lutra_maculicollis	0.25	0.37	0.65	0.00
Lutra_sumatrana	0.88	2.52	0.84	0.00
Lutreolina_crassicaudata	1.17	2.29	0.78	0.00
Lutrogale_perspicillata	1.63	3.64	0.79	0.00
Lycaon_pictus	0.01	0.02	0.54	0.00
Lynx_canadensis	0.85	0.83	-0.09	0.61
Lynx_lynx	0.83	1.15	0.52	0.00
Lynx_pardinus	2.06	3.56	0.67	0.00
Lynx_rufus	3.65	4.3	0.44	0.01
Macaca_arctoides	0.41	1.41	0.80	0.00
Macaca_assamensis	0.23	0.86	0.79	0.00
Macaca_cyclopis	11.77	17.25	0.81	0.00
Macaca_fascicularis	0.99	2.46	0.79	0.00
Macaca_fuscata	10.18	11.4	0.45	0.00
Macaca_hecki	0.04	0.25	0.44	0.01
Macaca_leonina	0.37	1.27	0.77	0.00
Macaca_maura	0.17	0.44	0.36	0.02
Macaca_mulatta	1.25	2.82	0.76	0.00
Macaca_munzala	0	0.08	0.39	0.02
Macaca_nemestrina	0.71	1.77	0.75	0.00

Macaca_nigra	0.93	2.45	0.47	0.00
Macaca_nigrescens	0.25	0.83	0.51	0.00
Macaca_ochreata	0.12	0.4	0.70	0.00
Macaca_pagensis	0.01	0.01	-0.17	0.30
Macaca_radiata	3.21	8.06	0.83	0.00
Macaca_siberu	0	0	-0.12	0.55
Macaca_silenus	0.92	2.85	0.57	0.00
Macaca_sinica	2.16	5.27	0.75	0.00
Macaca_sylvanus	0.95	2.92	0.85	0.00
Macaca_thibetana	0.42	1.83	0.79	0.00
Macaca_tonkeana	0.09	0.31	0.69	0.00
Macroderma_gigas	0.08	0.15	0.64	0.00
Macrogalidia_musschenbroekii	0.23	0.74	0.51	0.00
Macroglossus_minimus	0.68	1.53	0.75	0.00
Macroglossus_sobrinus	1.07	2.84	0.79	0.00
Macrophyllum_macrophyllum	0.53	1.05	0.75	0.00
Macropus_agilis	0.07	0.12	0.59	0.00
Macropus_antilopinus	0.03	0.04	0.48	0.00
Macropus_bernardus	0.03	0.05	0.43	0.01
Macropus_dorsalis	0.41	0.65	0.68	0.00
Macropus_eugenii	0.02	0.04	0.46	0.00
Macropus_fuliginosus	0.15	0.21	0.48	0.00
Macropus_giganteus	0.37	0.51	0.63	0.00
Macropus_irma	0.75	1.08	0.67	0.00
Macropus_parma	0.83	1.01	0.38	0.02
Macropus_parryi	0.67	1.06	0.68	0.00
Macropus_robustus	0.07	0.1	0.51	0.00
Macropus_rufogriseus	0.83	1.12	0.61	0.00
Macropus_rufus	0.02	0.03	0.47	0.00
Macroscelides_proboscideus	0.28	0.4	0.50	0.00
Macrotarsomys_bastardi	0	0.01	0.52	0.00
Macrotarsomys_ingens	0	0	-0.26	0.17
Macrotis_lagotis	0.01	0.02	0.61	0.00
Macrotus_californicus	1.56	2.17	0.63	0.00
Macrotus_waterhousii	2.63	4.15	0.64	0.00
Macruromys_major	0.05	0.1	0.32	0.04
Madoqua_guentheri	0	0.01	0.53	0.00
Madoqua_kirkii	0.06	0.13	0.65	0.00
Madoqua_saltiana	0.01	0.03	0.64	0.00
Madromys_blanfordi	2.26	5.56	0.77	0.00
Makalata_didelphoides	0.17	0.43	0.78	0.00
Makalata_macrura	0.04	0.1	0.73	0.00
Malacomys_cansdalei	0.41	1.13	0.69	0.00
Malacomys_edwardsi	1.16	1.62	0.45	0.00
Malacomys_longipes	0.81	0.71	-0.33	0.04
Malacothrix_typica	0.22	0.31	0.50	0.00

Mallomys_aroaensis	0.05	0.09	0.30	0.06
Mallomys_gunung	0	0	-0.12	0.55
Mallomys_istapantap	0.06	0.1	0.40	0.01
Mallomys_rothschildi	0.05	0.1	0.37	0.02
Mammelomys_lanosus	0.06	0.11	0.49	0.00
Mammelomys_rattoides	0.01	0.02	0.59	0.00
Mandrillus_leucophaeus	0.02	0.07	0.68	0.00
Mandrillus_sphinx	0.2	0.4	0.70	0.00
Manis_crassicaudata	2.62	5.45	0.74	0.00
Manis_culionensis	0.05	0.24	0.58	0.00
Manis_javanica	0.8	2	0.79	0.00
Manis_pentadactyla	0.91	2.65	0.81	0.00
Margaretamys_beccarii	0.28	0.93	0.53	0.00
Margaretamys_elegans	0	0	-0.32	0.07
Marmosa_alstoni	0.61	1.44	0.68	0.00
Marmosa_constantiae	0.21	0.38	0.70	0.00
Marmosa_demerarae	0.31	0.63	0.74	0.00
Marmosa_lepida	0.05	0.11	0.75	0.00
Marmosa_mexicana	1.33	2.63	0.66	0.00
Marmosa_murina	0.36	0.71	0.77	0.00
Marmosa_paraguayanus	2.07	4.17	0.80	0.00
Marmosa_phaeus	0.16	1.24	0.71	0.00
Marmosa_quichua	0.07	0.2	0.56	0.00
Marmosa_regina	0.18	0.32	0.73	0.00
Marmosa_robinsoni	2.63	4.18	0.70	0.00
Marmosa_xerophila	2.51	3.47	0.50	0.00
Marmosops_bishopi	0.11	0.22	0.70	0.00
Marmosops_handleyi	0.01	0.07	0.03	0.88
Marmosops_impavidus	0.54	1.16	0.72	0.00
Marmosops_incanus	1.93	3.53	0.75	0.00
Marmosops_invictus	1.27	2.45	0.79	0.00
Marmosops_juninensis	0.32	0.75	0.67	0.00
Marmosops_neblina	0.02	0.04	0.53	0.00
Marmosops_noctivagus	0.07	0.17	0.80	0.00
Marmosops_ocellatus	0.19	0.39	0.66	0.00
Marmosops_parvidens	0.05	0.13	0.71	0.00
Marmosops_paulensis	6.15	9.42	0.73	0.00
Marmosops_pinheiroi	0.14	0.34	0.79	0.00
Marmota_baibacina	0.81	1.13	0.42	0.01
Marmota_bobak	0.92	1.06	0.15	0.35
Marmota_broweri	0.01	0.01	0.16	0.33
Marmota_caligata	0.36	0.37	0.03	0.88
Marmota_camtschatica	0.07	0.05	-0.33	0.04
Marmota_caudata	0.3	0.36	0.45	0.00
Marmota_flaviventris	1.41	1.57	0.21	0.19
Marmota_himalayana	0.06	0.23	0.81	0.00

Marmota_marmota	6.8	11.29	0.81	0.00
Marmota_menzbieri	0	0	-0.12	0.55
Marmota_monax	4.14	4.53	0.23	0.16
Marmota_olympus	0.03	0.03	-0.03	0.88
Marmota_sibirica	0.07	0.19	0.70	0.00
Marmota_vancouverensis	0.83	0.63	-0.38	0.02
Martes_americana	0.62	0.62	-0.06	0.74
Martes_flavigula	1.03	2.58	0.83	0.00
Martes_foina	2.6	4.16	0.76	0.00
Martes_gwatkinsii	0.68	2.01	0.57	0.00
Martes_martes	4.16	6.25	0.73	0.00
Martes_melampus	11.89	13.49	0.57	0.00
Martes_pennanti	1.52	1.47	-0.10	0.57
Martes_zibellina	0.37	0.49	0.57	0.00
Massoutiera_mzabi	0.09	0.11	0.12	0.45
Mastacomys_fuscus	0.23	0.33	0.36	0.02
Mastomys_awashensis	0.18	0.61	0.69	0.00
Mastomys_coucha	0.75	1.2	0.70	0.00
Mastomys_erythroleucus	0.22	0.3	0.55	0.00
Mastomys_huberti	0.06	0.16	0.78	0.00
Mastomys_kollmannspergeri	0.01	0.06	0.72	0.00
Mastomys_natalensis	0.16	0.27	0.66	0.00
Mastomys_shortridgei	0.01	0.03	0.62	0.00
Maxomys_alticola	0.23	0.36	0.24	0.14
Maxomys_bartelsii	2.75	5.81	0.52	0.00
Maxomys_hellwaldii	0.25	0.73	0.61	0.00
Maxomys_inas	1.68	3.43	0.63	0.00
Maxomys_inflatus	0.14	0.53	0.49	0.00
Maxomys_moi	0.06	0.71	0.83	0.00
Maxomys_musschenbroekii	0.21	0.63	0.61	0.00
Maxomys_pagensis	0	0	-0.15	0.36
Maxomys_panglima	0.05	0.23	0.58	0.00
Maxomys_rajah	0.68	1.75	0.75	0.00
Maxomys_surifer	0.92	2.45	0.79	0.00
Maxomys_wattsi	0	0	-0.12	0.55
Maxomys_whiteheadi	0.68	1.75	0.75	0.00
Mazama_bororo	6.71	10.84	0.82	0.00
Mazama_bricenii	0.3	0.5	0.44	0.01
Mazama_chunyi	0.12	0.3	0.72	0.00
Mazama_gouazoubira	0.84	1.76	0.81	0.00
Mazama_nemorivaga	0.21	0.4	0.70	0.00
Mazama_pandora	0.93	1.69	0.61	0.00
Mazama_rufina	0.13	0.36	0.68	0.00
Megaderma_lyra	1.64	3.74	0.79	0.00
Megaderma_spasma	0.93	2.32	0.77	0.00
Megadontomys_cryophilus	0.14	0.28	0.38	0.02

Megadontomys_nelsoni	3.24	5.86	0.58	0.00
Megadontomys_thomasi	0	0	-0.08	0.69
Megaerops_ecaudatus	0.68	1.81	0.77	0.00
Megaerops_kusnotoi	3.67	7	0.66	0.00
Megaerops_niphanae	0.73	2.33	0.80	0.00
Megaerops_wetmorei	0.76	2.26	0.77	0.00
Megaloglossus_woermanni	0.37	0.42	0.14	0.38
Megasorex_gigas	1.66	2.4	0.51	0.00
Melanomys_caliginosus	1.25	2.21	0.64	0.00
Melanomys_robustulus	0.03	0.41	0.74	0.00
Melanomys_zunigae	4.94	11.01	0.73	0.00
Meles_anakuma	14.25	15.94	0.50	0.00
Meles_leucurus	1.02	1.93	0.77	0.00
Meles_meles	4.41	6.57	0.78	0.00
Mellivora_capensis	0.5	0.96	0.73	0.00
Melogale_moschata	1.19	3.5	0.84	0.00
Melomys_aerosus	0	0	-0.12	0.55
Melomys_bannisteri	0	0.02	0.17	0.32
Melomys_burtoni	0.46	0.69	0.69	0.00
Melomys_capensis	0.03	0.07	0.37	0.02
Melomys_caurinus	0.01	0.06	0.35	0.03
Melomys_cervinipes	1.17	1.68	0.66	0.00
Melomys_dollmani	0.13	0.22	0.24	0.14
Melomys_fraterculus	0	0	-0.12	0.55
Melomys_frigicola	0.04	0.16	0.54	0.00
Melomys_leucogaster	0.04	0.08	0.34	0.03
Melomys_lutillus	0.03	0.06	0.50	0.00
Melomys_matambuai	0.04	0.03	-0.17	0.29
Melomys_obiensis	0	0.11	0.14	0.43
Melomys_rufescens	0.04	0.09	0.53	0.00
Melomys_talaudium	0.01	0.06	0.35	0.03
Melonycteris_fardoulisi	0.05	0.09	0.26	0.11
Melonycteris_melanops	0.03	0.05	0.13	0.41
Melonycteris_woodfordi	0.01	0.02	0.25	0.12
Melursus_ursinus	2.3	4.91	0.72	0.00
Menetes_berdmorei	0.78	2.4	0.79	0.00
Mephitis_macroura	1.78	2.81	0.67	0.00
Mephitis_mephitis	3.93	4.45	0.39	0.01
Meriones_arimalius	0.15	0.37	0.50	0.00
Meriones_chengi	0.53	0.7	0.01	0.98
Meriones_crassus	1.07	1.9	0.71	0.00
Meriones_dahli	1.37	4.92	0.77	0.00
Meriones_grandis	1.31	3.22	0.81	0.00
Meriones_hurrianae	1.87	3.71	0.72	0.00
Meriones_libycus	1.05	1.83	0.73	0.00
Meriones_meridianus	0.5	0.96	0.73	0.00

Meriones_persicus	0.84	1.87	0.75	0.00
Meriones_rex	2.27	4.93	0.83	0.00
Meriones_sacramenti	15.45	24.3	0.79	0.00
Meriones_shawi	1.65	3.85	0.84	0.00
Meriones_tamariscinus	0.52	0.79	0.59	0.00
Meriones_tristrami	2.56	5.09	0.80	0.00
Meriones_unguiculatus	0.42	0.98	0.78	0.00
Meriones_vinogradovi	1.88	4.08	0.77	0.00
Mesechinus_dauuricus	0.54	1.38	0.77	0.00
Mesechinus_hughi	1	3.02	0.81	0.00
Mesembriomys_gouldii	0.16	0.26	0.63	0.00
Mesembriomys_macrurus	0	0	0.15	0.35
Mesocapromys_angelcabrerai	0	0	-0.12	0.55
Mesocapromys_auritus	0	0	-0.12	0.55
Mesocapromys_nanus	0.27	0.7	0.53	0.00
Mesocapromys_sanfelipensis	0	0	-0.12	0.55
Mesocricetus_auratus	4.1	9.51	0.74	0.00
Mesocricetus_brandti	1.55	3.33	0.72	0.00
Mesocricetus_newtoni	3.24	4.55	0.49	0.00
Mesocricetus_raddei	2.7	2.63	0.01	0.98
Mesomys_hispidus	0.05	0.12	0.77	0.00
Mesomys_occultus	0.09	0.12	0.10	0.53
Mesomys_stimulax	0.12	0.33	0.74	0.00
Mesophylla_macconnelli	0.32	0.58	0.70	0.00
Metachirus_nudicaudatus	0.47	0.92	0.74	0.00
Mico_argentatus	0.05	0.16	0.75	0.00
Mico_intermedius	0	0.02	0.63	0.00
Mico_leucippe	0	0.08	0.72	0.00
Mico_manicorensis	0.01	0.04	0.62	0.00
Mico_mauesi	0	0	-0.12	0.55
Mico_melanurus	0.14	0.26	0.72	0.00
Mico_rondoni	0.17	0.59	0.80	0.00
Mico_saterei	0	0	0.61	0.00
Microakodontomys_transitorius	16.33	32.93	0.89	0.00
Microcavia_australis	0.26	0.62	0.75	0.00
Microcavia_niata	0.07	0.2	0.78	0.00
Microcavia_shiptoni	0.01	0.14	0.73	0.00
Microcebus_berthae	0	0	-0.12	0.55
Microcebus_griseorufus	0.01	0.02	0.41	0.01
Microcebus_murinus	0	0.01	0.45	0.00
Microcebus_ravelobensis	0	0	-0.28	0.10
Microcebus_rufus	0	0.01	0.30	0.07
Microcebus_sambiranensis	0	0	-0.06	0.76
Microcebus_tavaratra	0	0.01	0.12	0.51
Microdillus_peeli	0	0.03	0.58	0.00
Microdipodops_megacephalus	0.48	0.53	0.32	0.04

Microdipodops_pallidus	0.4	0.46	0.30	0.07
Microgale_brevicaudata	0.01	0.02	0.27	0.10
Microgale_cowani	0	0.01	0.50	0.00
Microgale_dobsoni	0.01	0.02	0.57	0.00
Microgale_drouhardi	0	0.01	0.55	0.00
Microgale_dryas	0	0	0.01	1.00
Microgale_fotsifotsy	0	0.01	0.54	0.00
Microgale_gracilis	0	0.02	0.50	0.00
Microgale_gymnorhyncha	0	0.02	0.50	0.00
Microgale_jenkinsae	0	0	-0.12	0.55
Microgale_jobihely	0	0	-0.12	0.55
Microgale_longicaudata	0	0.01	0.12	0.51
Microgale_majori	0	0.02	0.50	0.00
Microgale_monticola	0	0	-0.12	0.55
Microgale_nasoloi	0	0	-0.12	0.55
Microgale_parvula	0	0.01	0.50	0.00
Microgale_principula	0	0.02	0.51	0.00
Microgale_pusilla	0.09	0.16	0.65	0.00
Microgale_soricoides	0	0.01	0.49	0.00
Microgale_taiva	0	0.01	0.37	0.02
Microgale_talazaci	0	0.01	0.50	0.00
Microgale_thomasi	0	0.03	0.55	0.00
Micromys_minutus	2.81	4.43	0.75	0.00
Micronycteris_hirsuta	0.47	0.92	0.70	0.00
Micronycteris_megalotis	0.49	0.94	0.74	0.00
Micronycteris_microtis	0.39	0.73	0.69	0.00
Micronycteris_minuta	0.52	1	0.75	0.00
Micronycteris_schmidtorum	0.59	1.08	0.70	0.00
Microperoryctes_longicauda	0.04	0.09	0.37	0.02
Microperoryctes_papuensis	0	0	-0.05	0.76
Micropotamogale_lamottei	0.09	0.18	0.33	0.04
Micropotamogale_ruwenzorii	0.09	0.18	0.36	0.02
Micropteropus_pusillus	0.1	0.22	0.75	0.00
Microryzomys_altissimus	0.56	1.56	0.77	0.00
Microryzomys_minutus	1.01	1.67	0.68	0.00
Microsciurus_alfari	1.48	2.72	0.67	0.00
Microsciurus_mimulus	0.94	1.39	0.59	0.00
Microtus_abbreviatus	0	0	-0.12	0.55
Microtus_agrestis	3.22	4.54	0.69	0.00
Microtus_arvalis	4.05	6.06	0.69	0.00
Microtus_bavaricus	1.26	3.62	0.45	0.00
Microtus_brachycercus	12.39	18.02	0.82	0.00
Microtus_breweri	0	0	-0.12	0.55
Microtus_cabrerae	3.64	7.17	0.81	0.00
Microtus_californicus	7.96	8.4	0.33	0.04
Microtus_canicaudus	23.06	24.03	0.28	0.09

Microtus_chrotorrhinus	3.59	3.6	-0.05	0.79
Microtus_clarkei	0.2	1.23	0.80	0.00
Microtus_daghestanicus	1.03	1.74	0.52	0.00
Microtus_dogramacii	1.38	2	0.28	0.09
Microtus_duodecimcostatus	5.87	9.32	0.86	0.00
Microtus_fortis	1.12	3.13	0.86	0.00
Microtus_gerbei	5.54	8.58	0.82	0.00
Microtus_gregalis	0.72	1.12	0.62	0.00
Microtus_guatemalensis	2.01	5.54	0.82	0.00
Microtus_guentheri	2.89	5.54	0.78	0.00
Microtus_hyperboreus	0.02	0.01	-0.22	0.17
Microtus_ilaeus	1.17	1.65	0.48	0.00
Microtus_kikuchii	0.01	0.05	0.37	0.02
Microtus_levis	3.06	4	0.30	0.06
Microtus_liechtensteini	4.87	9.62	0.81	0.00
Microtus_limnophilus	0.06	0.29	0.78	0.00
Microtus_longicaudus	1.14	1.24	0.16	0.32
Microtus_lusitanicus	5.36	9.8	0.87	0.00
Microtus_majori	2.27	2.97	0.36	0.02
Microtus_maximowiczii	0.33	0.97	0.76	0.00
Microtus_mexicanus	2.04	3.03	0.65	0.00
Microtus_middendorffii	0.22	0.36	0.50	0.00
Microtus_miurus	0.1	0.11	0.00	1.00
Microtus_mongolicus	0.4	1.13	0.71	0.00
Microtus_montanus	1.56	1.68	0.15	0.35
Microtus_montebelli	14.55	16.39	0.57	0.00
Microtus_multiplex	11.03	16.54	0.87	0.00
Microtus_oaxacensis	0	0.04	0.26	0.11
Microtus_ochrogaster	4.74	5.57	0.34	0.03
Microtus_oeconomus	0.9	1.26	0.51	0.00
Microtus_oregoni	4.45	4.4	0.00	1.00
Microtus_paradoxus	0.86	2.03	0.66	0.00
Microtus_pennsylvanicus	2.7	2.94	0.23	0.16
Microtus_pinetorum	9.71	11.24	0.52	0.00
Microtus_quasiater	5.54	8.43	0.60	0.00
Microtus_richardsoni	1.67	1.79	0.16	0.32
Microtus_sachalinensis	0.72	0.93	0.36	0.02
Microtus_savii	15.27	22	0.85	0.00
Microtus_schelkovnikovi	0.66	1.6	0.62	0.00
Microtus_schidlovskii	0.6	1.69	0.59	0.00
Microtus_socialis	1.32	2.35	0.69	0.00
Microtus_subterraneus	5.59	8.83	0.70	0.00
Microtus_tatricus	2.63	3.46	0.29	0.07
Microtus_thomasi	2.3	5.01	0.70	0.00
Microtus_townsendii	4.26	4.22	0.02	0.93
Microtus_transcaspicus	0.48	1.96	0.72	0.00

Microtus_umbrosus	0.38	1.33	0.65	0.00
Microtus_xanthognathus	0.3	0.33	0.15	0.35
Millardia_gleadowi	0.94	2.01	0.59	0.00
Millardia_kathleenae	0.3	0.62	0.26	0.11
Millardia_kondana	5.21	11.47	0.82	0.00
Millardia_meltada	2.92	5.93	0.74	0.00
Mimetillus_moloneyi	0.4	0.51	0.37	0.02
Mimon_bennettii	1.04	2.06	0.78	0.00
Mimon_cozumelae	1.52	2.46	0.61	0.00
Mimon_crenulatum	0.42	0.83	0.71	0.00
Mindomys_hammondi	7.13	12.68	0.90	0.00
Miniopterus_australis	0.69	1.33	0.69	0.00
Miniopterus_fraterculus	0.33	0.55	0.67	0.00
Miniopterus_fuliginosus	1.7	3.47	0.79	0.00
Miniopterus_fuscus	15.5	13.73	-0.38	0.02
Miniopterus_gleni	0.02	0.04	0.47	0.00
Miniopterus_inflatus	0.02	0.06	0.68	0.00
Miniopterus_magnater	0.58	1.76	0.81	0.00
Miniopterus_majori	0.01	0.02	0.47	0.00
Miniopterus_manavi	0.02	0.04	0.54	0.00
Miniopterus_medius	1.02	2.49	0.79	0.00
Miniopterus_natalensis	0.47	0.89	0.75	0.00
Miniopterus_oceanensis	0.44	0.63	0.66	0.00
Miniopterus_pusillus	0.85	2.41	0.79	0.00
Miniopterus_robustior	0.07	0.37	0.53	0.00
Miniopterus_schreibersii	3.92	6.79	0.84	0.00
Miniopterus_sororculus	0.07	0.12	0.56	0.00
Miniopterus_tristis	0.41	0.87	0.63	0.00
Miopithecus_ogouensis	0.16	0.35	0.72	0.00
Miopithecus_talapoin	0.06	0.3	0.79	0.00
Mirimiri_acrodonta	0.09	0	-0.18	0.30
Mirza_coquereli	0	0	0.33	0.04
Mirza_zaza	0.01	0.03	0.23	0.16
Molossops_aequatorianus	12.96	18.98	0.80	0.00
Molossops_mattogrossensis	0.28	0.63	0.72	0.00
Molossops_temminckii	0.41	0.87	0.78	0.00
Molossus_aztecus	6.32	7.85	0.66	0.00
Molossus_coibensis	0.2	0.44	0.73	0.00
Molossus_currentium	1.18	2.02	0.68	0.00
Molossus_molossus	0.65	1.23	0.73	0.00
Molossus_pretiosus	1.11	1.78	0.61	0.00
Molossus_rufus	0.58	1.14	0.73	0.00
Molossus_sinaloae	1.7	2.82	0.65	0.00
Monodelphis_adusta	0.38	0.58	0.57	0.00
Monodelphis_americana	1.92	3.83	0.77	0.00
Monodelphis_brevicaudata	0.05	0.12	0.76	0.00

Monodelphis_dimidiata	1.57	3.05	0.80	0.00
Monodelphis_domestica	0.42	0.95	0.76	0.00
Monodelphis_emiliae	0.06	0.18	0.76	0.00
Monodelphis_glirina	0.03	0.11	0.76	0.00
Monodelphis_handleyi	0.05	0.47	0.38	0.02
Monodelphis_kunsi	0.34	0.69	0.77	0.00
Monodelphis_osgoodi	0.13	0.3	0.70	0.00
Monodelphis_palliolata	4.24	5.49	0.62	0.00
Monodelphis_reigi	0.05	0.28	0.02	0.95
Monodelphis_ronaldi	0	0	-0.12	0.55
Monodelphis_scalops	3.18	5.82	0.77	0.00
Monodelphis_umbristriata	0.86	1.87	0.73	0.00
Monodelphis_unistriata	3.48	5.77	0.65	0.00
Monophyllus_plethodon	12.36	19.65	0.85	0.00
Monophyllus_redmani	2.75	4.35	0.60	0.00
Monticolomys_koopmani	0.02	0.04	0.12	0.45
Mormoops_blainvillei	2.77	4.38	0.60	0.00
Mormoops_megalophylla	1.69	2.72	0.68	0.00
Mormopterus_acetabulosus	7.95	14.49	0.80	0.00
Mormopterus_beccarii	0.08	0.13	0.64	0.00
Mormopterus_jugularis	0.03	0.07	0.57	0.00
Mormopterus_kalinowskii	0.55	1.23	0.79	0.00
Mormopterus_loriae	0.15	0.24	0.63	0.00
Mormopterus_minutus	0.63	1.83	0.50	0.00
Mormopterus_norfolkensis	4.75	5.79	0.63	0.00
Mormopterus_phrudus	0	0	0.24	0.20
Mormopterus_planiceps	0.3	0.4	0.58	0.00
Moschiola_indica	2.21	4.85	0.70	0.00
Moschiola_kathygre	2.55	7.24	0.74	0.00
Moschiola_meminna	2.09	5.09	0.76	0.00
Moschus_anhuiensis	0.03	0.35	0.58	0.00
Moschus_berezovskii	0.33	1.41	0.78	0.00
Moschus_chrysogaster	0.07	0.3	0.81	0.00
Moschus_cupreus	0.01	0.04	0.61	0.00
Moschus_fuscus	0.01	0.09	0.77	0.00
Moschus_leucogaster	0.08	0.32	0.74	0.00
Moschus_moschiferus	0.37	0.64	0.75	0.00
Mosia_nigrescens	0.07	0.17	0.57	0.00
Mungos_gambianus	0.46	0.68	0.50	0.00
Mungos_mungo	0.13	0.24	0.72	0.00
Mungotictis_decemlineata	0	0	-0.23	0.15
Muntiacus_atherodes	0.24	0.57	0.71	0.00
Muntiacus_crinifrons	1.1	4.56	0.86	0.00
Muntiacus_muntjak	1.11	2.54	0.77	0.00
Muntiacus_reevesi	1.55	4.6	0.85	0.00
Muntiacus_vaginalis	1.53	3.61	0.79	0.00

Muntiacus_vuquangensis	0.03	0.61	0.78	0.00
Murexia_habbema	0.06	0.12	0.34	0.03
Murexia_longicaudata	0.04	0.09	0.50	0.00
Murexia_melanurus	0.05	0.1	0.39	0.01
Murexia_naso	0.04	0.1	0.35	0.03
Murexia_rothschildi	0	0	-0.20	0.25
Muriculus_imberbis	0.36	0.89	0.72	0.00
Murina_aenea	0.68	2.18	0.72	0.00
Murina_aurata	0.29	1.75	0.84	0.00
Murina_cyclotis	0.83	2.58	0.84	0.00
Murina_florium	0.15	0.32	0.53	0.00
Murina_hilgendorfi	1.6	1.95	0.59	0.00
Murina_huttoni	0.49	1.48	0.77	0.00
Murina_puta	13.29	18.89	0.82	0.00
Murina_rozendaali	0.23	0.92	0.57	0.00
Murina_ryukyuana	2.51	2.11	0.05	0.79
Murina_suilla	1.07	2.47	0.77	0.00
Murina_tenebrosa	14.14	8.49	-0.59	0.00
Murina_tubinaris	0.31	0.93	0.74	0.00
Murina_ussuriensis	3.85	4.67	0.42	0.01
Mus_baoulei	0.18	0.65	0.57	0.00
Mus_booduga	2.5	5.17	0.76	0.00
Mus_bufo	0.14	0.28	0.46	0.00
Mus_callewaerti	0	0	-0.30	0.10
Mus_caroli	1.4	3.65	0.87	0.00
Mus_cervicolor	1.84	4	0.76	0.00
Mus_cookii	0.36	0.95	0.70	0.00
Mus_cypriacus	8.04	14.42	0.89	0.00
Mus_famulus	1.01	3.19	0.60	0.00
Mus_fernandoni	1.31	6.79	0.72	0.00
Mus_haussa	0.06	0.13	0.67	0.00
Mus_indutus	0.14	0.31	0.75	0.00
Mus_macedonicus	2.46	4.67	0.74	0.00
Mus_mahomet	0.05	0.18	0.73	0.00
Mus_mattheyi	0.12	0.32	0.58	0.00
Mus_mayori	3.53	7.64	0.70	0.00
Mus_minutoides	0.18	0.31	0.63	0.00
Mus_musculoides	0.24	0.37	0.68	0.00
Mus_musculus	1.81	2.74	0.72	0.00
Mus_orangiae	0.96	1.29	0.56	0.00
Mus_pahari	0.29	1.08	0.79	0.00
Mus_phillipsi	2.79	6.38	0.67	0.00
Mus_platythrix	2.93	7.09	0.83	0.00
Mus_saxicola	2.7	5.78	0.73	0.00
Mus_setulosus	0.18	0.48	0.72	0.00
Mus_setzeri	0.01	0.02	0.43	0.01

Mus_shortridgei	0.13	0.53	0.65	0.00
Mus_sorella	0	0	0.08	0.65
Mus_spicilegus	4	4.9	0.31	0.05
Mus_spretus	4.17	7.61	0.85	0.00
Mus_tenellus	0.02	0.07	0.68	0.00
Mus_terricolor	2.63	5.44	0.76	0.00
Mus_triton	0.14	0.3	0.70	0.00
Mus_vulcani	0.29	1.81	0.42	0.01
Muscardinus_avellanarius	6.38	9.84	0.75	0.00
Musonycteris_harrisoni	1.81	2.5	0.45	0.00
Mustela_africana	0.05	0.14	0.76	0.00
Mustela_altaica	0.46	1.01	0.82	0.00
Mustela_erminea	1.67	2.15	0.55	0.00
Mustela_eversmanii	1.31	1.87	0.56	0.00
Mustela_felipei	0.54	1.4	0.64	0.00
Mustela_frenata	4.13	4.9	0.50	0.00
Mustela_itatsi	12.34	13.93	0.52	0.00
Mustela_kathiah	0.72	2.47	0.81	0.00
Mustela_lutreola	1.97	2.4	0.20	0.22
Mustela_nigripes	1.6	1.97	0.46	0.00
Mustela_nivalis	1.77	2.45	0.66	0.00
Mustela_nudipes	0.66	1.68	0.74	0.00
Mustela_putorius	5.26	8	0.73	0.00
Mustela_sibirica	1.18	2.36	0.80	0.00
Mustela_strigidorsa	0.26	1.15	0.79	0.00
Mustela_subpalmata	14.85	29.71	0.95	0.00
Mydaus_javanensis	0.44	0.92	0.58	0.00
Mydaus_marchei	0.05	0.24	0.58	0.00
Mylomys_dybowskii	0.06	0.12	0.50	0.00
Myocastor_coypus	0.59	1.19	0.78	0.00
Myodes_andersoni	4.79	5.93	0.31	0.05
Myodes_californicus	2.58	2.53	-0.03	0.88
Myodes_centralis	0.16	0.29	0.51	0.00
Myodes_gapperi	2.58	2.69	0.04	0.83
Myodes_glareolus	4.24	5.99	0.66	0.00
Myodes_regulus	8.05	12.32	0.82	0.00
Myodes_rex	3.02	3.69	0.09	0.61
Myodes_rufocanus	0.59	0.78	0.60	0.00
Myodes_rutilus	0.51	0.67	0.53	0.00
Myodes_shanseius	2.78	5.63	0.81	0.00
Myodes_smithii	9	10.11	0.47	0.00
Myoictis_melas	0.04	0.09	0.62	0.00
Myoictis_wallacei	0.01	0.03	0.47	0.00
Myomimus_roachi	2.85	5.13	0.66	0.00
Myomyscus_angolensis	0.01	0.14	0.70	0.00
Myomyscus_brockmani	0.04	0.08	0.61	0.00

Myomyscus_verreauxii	1.62	2.18	0.61	0.00
Myomyscus_yemeni	3.71	8.31	0.86	0.00
Myonycteris_brachycephala	0.06	0.6	0.49	0.00
Myonycteris_relicta	0.23	0.37	0.68	0.00
Myonycteris_torquata	0.13	0.24	0.72	0.00
Myoprocta_acouchy	0.05	0.13	0.70	0.00
Myoprocta_pratti	0.07	0.15	0.80	0.00
Myopterus_whitleyi	0.46	0.37	-0.49	0.00
Myopus_schisticolor	0.55	0.66	0.48	0.00
Myosciurus_pumilio	0.23	0.38	0.56	0.00
Myosorex_babaulti	0.12	0.29	0.50	0.00
Myosorex_blarina	0.01	0	-0.18	0.29
Myosorex_cafer	1.08	2.22	0.70	0.00
Myosorex_eisentrauti	0.96	0	-0.63	0.00
Myosorex_geata	0.22	0.14	-0.33	0.04
Myosorex_kihaulei	0	0	-0.12	0.55
Myosorex_longicaudatus	2.16	2.56	0.46	0.00
Myosorex_okuensis	0.01	0.07	0.23	0.15
Myosorex_rumpii	0	0	-0.12	0.55
Myosorex_sclateri	3.03	5.02	0.62	0.00
Myosorex_varius	1.2	2.1	0.71	0.00
Myosorex_zinki	0.07	0	-0.43	0.01
Myotis_adversus	4.38	8.98	0.87	0.00
Myotis_albescens	0.55	1.08	0.74	0.00
Myotis_altarium	1.03	3.25	0.84	0.00
Myotis_annectans	0.35	1.03	0.64	0.00
Myotis_atacamensis	0.64	1.26	0.75	0.00
Myotis_ater	0.12	0.36	0.62	0.00
Myotis_aurascens	2.24	3.22	0.58	0.00
Myotis_auriculus	0.87	1.34	0.64	0.00
Myotis_austroriparius	6.84	8.5	0.50	0.00
Myotis_bechsteinii	6.86	11.13	0.86	0.00
Myotis_blythii	3.06	5.14	0.82	0.00
Myotis_bocagii	0.17	0.42	0.75	0.00
Myotis_bombinus	0.98	1.29	0.67	0.00
Myotis_brandtii	3.76	5.29	0.56	0.00
Myotis_californicus	2.12	2.62	0.58	0.00
Myotis_capaccinii	5.43	9.06	0.86	0.00
Myotis_chiloensis	0.59	1.32	0.78	0.00
Myotis_chinensis	0.93	2.97	0.84	0.00
Myotis_ciliolabrum	1.31	1.48	0.18	0.26
Myotis_dasycneme	3.14	4.46	0.50	0.00
Myotis_daubentonii	2.95	4.29	0.68	0.00
Myotis_davidii	3.68	6.21	0.74	0.00
Myotis_dinellii	0.38	0.86	0.77	0.00
Myotis_dominicensis	9.93	15.36	0.76	0.00

Myotis_elegans	1.14	2.21	0.64	0.00
Myotis_emarginatus	4.38	7.17	0.90	0.00
Myotis_evotis	2.11	2.31	0.25	0.12
Myotis_fimbriatus	1.02	3.35	0.83	0.00
Myotis_findleyi	0.64	1.45	0.03	0.88
Myotis_formosus	1.58	3.54	0.81	0.00
Myotis_fortidens	1.88	2.99	0.61	0.00
Myotis_gomantongensis	0.24	0.57	0.71	0.00
Myotis_goudoti	0.02	0.04	0.53	0.00
Myotis_grisescens	7.82	9.56	0.47	0.00
Myotis_hajastanicus	1.28	5.04	0.84	0.00
Myotis_hasseltii	0.93	2.08	0.74	0.00
Myotis_horsfieldii	1.11	2.84	0.81	0.00
Myotis_ikonnikovi	1.23	1.87	0.63	0.00
Myotis_keaysi	1.15	2.05	0.70	0.00
Myotis_keenii	2.46	2.28	-0.23	0.16
Myotis_laniger	1.4	4.23	0.86	0.00
Myotis_leibii	11.11	12.04	0.30	0.06
Myotis_levis	1.6	3.12	0.81	0.00
Myotis_lucifugus	3.3	3.66	0.31	0.05
Myotis_macrodactylus	8.88	10.88	0.70	0.00
Myotis_macropus	0.59	0.81	0.65	0.00
Myotis_macrotarsus	0.9	1.8	0.62	0.00
Myotis_martiniquensis	20.11	30.7	0.80	0.00
Myotis_melanorhinus	1.98	2.47	0.61	0.00
Myotis_moluccarum	0.08	0.19	0.55	0.00
Myotis_montivagus	0.77	2.44	0.84	0.00
Myotis_muricola	0.82	2.12	0.79	0.00
Myotis_myotis	6.19	10.12	0.86	0.00
Myotis_mystacinus	5.39	8.18	0.72	0.00
Myotis_nattereri	5.91	9.08	0.84	0.00
Myotis_nesopolus	3.7	4.34	0.38	0.02
Myotis_nigricans	0.57	1.12	0.73	0.00
Myotis_nipalensis	0.86	1.4	0.72	0.00
Myotis_occultus	1.42	2.05	0.66	0.00
Myotis_oxyotus	0.96	1.66	0.68	0.00
Myotis_peninsularis	0.06	0.3	0.49	0.00
Myotis_pequinius	3.12	8.34	0.84	0.00
Myotis_pilosus	2.6	7.26	0.85	0.00
Myotis_planiceps	0.2	0.59	0.67	0.00
Myotis_pruinosus	8.45	9.61	0.45	0.00
Myotis_punicus	1.78	4.16	0.83	0.00
Myotis_ridleyi	0.61	1.75	0.83	0.00
Myotis_riparius	0.51	1.02	0.75	0.00
Myotis_rosseti	1.39	3.79	0.79	0.00
Myotis_ruber	1.47	3.04	0.82	0.00

Myotis_scotti	0.01	0.08	0.75	0.00
Myotis_septentrionalis	5.98	6.59	0.26	0.11
Myotis_sicarius	0.44	0.97	0.58	0.00
Myotis_siligorensis	0.56	2.05	0.80	0.00
Myotis_sodalis	9.5	10.88	0.48	0.00
Myotis_thysanodes	2.28	2.8	0.59	0.00
Myotis_tricolor	0.28	0.46	0.71	0.00
Myotis_velifer	2.34	3.39	0.65	0.00
Myotis_vivesi	0.34	0.52	0.53	0.00
Myotis_volans	2.03	2.37	0.43	0.01
Myotis_welwitschii	0.26	0.45	0.73	0.00
Myotis_yanbarensis	0.35	0.47	0.16	0.32
Myotis_yumanensis	1.98	2.35	0.52	0.00
Myrmecobius_fasciatus	0	0.01	0.41	0.01
Myrmecophaga_tridactyla	0.4	0.84	0.77	0.00
Mysateles_garridoi	2.35	1.85	-0.33	0.04
Mysateles_gundlachi	0.16	0.43	0.48	0.00
Mysateles_melanurus	0.67	2.5	0.66	0.00
Mysateles_meridionalis	0	0.03	0.07	0.71
Mysateles_prehensilis	0.93	2.54	0.60	0.00
Mystacina_robusta	0	0	-0.12	0.55
Mystacina_tuberculata	0.1	0.14	0.19	0.24
Mystromys_albicaudatus	1.76	2.47	0.70	0.00
Myzopoda_aurita	0.01	0.04	0.50	0.00
Myzopoda_schliemanni	0.02	0.03	0.02	0.93
Naemorhedus_baileyi	0.01	0.14	0.83	0.00
Naemorhedus_caudatus	0.8	1.85	0.77	0.00
Naemorhedus_goral	0.09	0.3	0.70	0.00
Naemorhedus_griseus	0.53	1.71	0.79	0.00
Nandinia_binotata	0.25	0.33	0.41	0.01
Nanger_dama	0	0	0.43	0.01
Nanger_granti	0.04	0.09	0.59	0.00
Nanger_soemmerringii	0.02	0.05	0.61	0.00
Nannosciurus_melanotis	0.69	1.33	0.54	0.00
Nanonycteris_veldkampii	0.75	0.89	0.27	0.10
Napaeozapus_insignis	5.21	5.14	-0.08	0.65
Nasalis_larvatus	0.26	0.61	0.74	0.00
Nasua_narica	1.49	2.44	0.67	0.00
Nasua_nasua	0.32	0.69	0.80	0.00
Natalus_espiritosantensis	0.75	1.59	0.71	0.00
Natalus_jamaicensis	7.38	9.89	0.50	0.00
Natalus_lanatus	1.75	2.72	0.55	0.00
Natalus_major	1.5	2.95	0.56	0.00
Natalus_mexicanus	1.68	2.7	0.69	0.00
Natalus_primus	0.3	0.74	0.50	0.00
Natalus_stramineus	13.13	20.69	0.85	0.00

Natalus_tumidirostris	1.03	1.65	0.63	0.00
Neacomys_dubosti	0.04	0.08	0.69	0.00
Neacomys_guianae	0.04	0.08	0.53	0.00
Neacomys_minutus	0	0.01	0.64	0.00
Neacomys_musseri	0.01	0.05	0.76	0.00
Neacomys_paracou	0.05	0.12	0.69	0.00
Neacomys_spinosus	0.09	0.21	0.75	0.00
Neacomys_tenuipes	2.81	3.88	0.60	0.00
Necromys_amoenus	0.28	0.73	0.77	0.00
Necromys_benefactus	0.93	1.95	0.72	0.00
Necromys_lactens	0.29	0.79	0.80	0.00
Necromys_lasiurus	0.71	1.46	0.75	0.00
Necromys_lenguarum	0.14	0.17	0.07	0.69
Necromys_obscurus	2.63	4.54	0.61	0.00
Necromys_temchuki	0.43	1.02	0.73	0.00
Necromys_urichi	1.7	2.45	0.68	0.00
Nectogale_elegans	0.36	1.48	0.84	0.00
Nectomys_apicalis	0.08	0.15	0.72	0.00
Nectomys_palmipes	0.79	1.55	0.72	0.00
Nectomys_rattus	0.2	0.39	0.70	0.00
Nectomys_squamipes	1.4	2.87	0.80	0.00
Nelsonia_goldmani	1.09	2.23	0.50	0.00
Nelsonia_neotomodon	0.23	0.37	0.31	0.05
Neodon_irene	0.03	0.27	0.84	0.00
Neodon_juldaschi	0.76	0.82	0.26	0.11
Neodon_sikimensis	0.09	0.23	0.74	0.00
Neofelis_diardi	0.03	0.11	0.55	0.00
Neofelis_nebulosa	0.44	1.64	0.80	0.00
Neofiber_alleni	12.67	15.27	0.70	0.00
Neohylomys_hainanensis	0.36	1.56	0.63	0.00
Neomys_anomalus	5.53	8.86	0.82	0.00
Neomys_fodiens	3.17	4.46	0.66	0.00
Neomys_teres	1.69	2.42	0.47	0.00
Neonycteris_pusilla	0	0	0.48	0.00
Neophascogale_lorentzii	0.03	0.08	0.49	0.00
Neopteryx_frosti	0.21	0.71	0.45	0.00
Neotetracus_sinensis	0.1	0.86	0.78	0.00
Neotoma_albigula	1.17	1.65	0.64	0.00
Neotoma_angustapalata	0.06	0.12	0.20	0.22
Neotoma_bryanti	0.74	1.11	0.50	0.00
Neotoma_chrysomelas	0.74	2.14	0.70	0.00
Neotoma_cinerea	1.25	1.36	0.17	0.29
Neotoma_devia	2.06	3.12	0.74	0.00
Neotoma_floridana	6.89	8.6	0.58	0.00
Neotoma_fuscipes	5	5.02	0.10	0.53
Neotoma_goldmani	0.8	1.35	0.61	0.00

Neotoma_lepida	2.23	2.57	0.58	0.00
Neotoma_leucodon	1.42	1.99	0.53	0.00
Neotoma_macrotis	7.04	7.45	0.35	0.03
Neotoma_magister	10.59	12.18	0.42	0.01
Neotoma_mexicana	1.93	2.83	0.67	0.00
Neotoma_micropus	1.66	2.18	0.52	0.00
Neotoma_nelsoni	3.12	6.21	0.53	0.00
Neotoma_palatina	0.28	0.54	0.46	0.00
Neotoma_phenax	2.32	3.51	0.47	0.00
Neotoma_stephensi	0.78	1.03	0.54	0.00
Neotomodon_alstoni	8.3	11.97	0.69	0.00
Neotomys_ebriosus	0.12	0.34	0.71	0.00
Neotragus_batesi	0.45	0.37	-0.36	0.02
Neotragus_pygmaeus	0.27	0.76	0.70	0.00
Neovison_vison	3	3.37	0.35	0.03
Nephelomys_albigularis	1.21	2.1	0.68	0.00
Nephelomys_auriventer	0.1	0.96	0.73	0.00
Nephelomys_caracolus	18.52	20.68	0.53	0.00
Nephelomys_devius	4.48	6.29	0.61	0.00
Nephelomys_keaysi	0.13	0.34	0.70	0.00
Nephelomys_levipes	0.25	0.6	0.78	0.00
Nephelomys_meridensis	2.01	2.8	0.52	0.00
Nesokia_bunnii	2.06	5.75	0.80	0.00
Nesokia_indica	1.59	2.79	0.78	0.00
Nesolagus_netscheri	0.05	0.3	0.70	0.00
Nesomys_audeberti	0	0.03	0.58	0.00
Nesomys_lambertoni	0	0	-0.12	0.55
Nesomys_rufus	0	0.02	0.57	0.00
Nesoromys_ceramicus	0	0	-0.12	0.55
Nesoryzomys_fernandinae	0.07	0.11	-0.08	0.69
Nesoryzomys_narboroughi	0.07	0.11	-0.08	0.69
Nesoryzomys_swarthi	0	0	-0.03	0.92
Nesotragus_moschatus	0.09	0.21	0.70	0.00
Neurotrichus_gibbsii	5.32	5.2	-0.05	0.79
Neusticomys_monticolus	2.73	4.39	0.73	0.00
Neusticomys_mussoi	2.87	3.69	0.60	0.00
Neusticomys_peruviensis	0	0	-0.12	0.55
Neusticomys_venezuelae	0.1	0.28	0.56	0.00
Nilgiritragus_hylocrius	1.16	2.44	0.46	0.00
Nilopegamys_plumbeus	0	0	-0.12	0.55
Ningaui_ridei	0.01	0.01	0.10	0.57
Ningaui_timealeyi	0.06	0.21	0.72	0.00
Ningaui_yvonneae	0.07	0.1	0.24	0.14
Niviventer_andersoni	0.2	1.17	0.83	0.00
Niviventer_brahma	0.04	0.06	0.25	0.12
Niviventer_cameroni	1.58	2.23	0.37	0.02

Niviventer_confucianus	1.49	4.31	0.84	0.00
Niviventer_coninga	16.81	23.85	0.82	0.00
Niviventer_cremoriventer	1.27	2.9	0.78	0.00
Niviventer_culturatus	0.12	0.39	0.50	0.00
Niviventer_eha	0.12	0.38	0.77	0.00
Niviventer_excelsior	0.07	0.75	0.81	0.00
Niviventer_fraternus	0.1	0.4	0.50	0.00
Niviventer_fulvescens	0.87	2.59	0.81	0.00
Niviventer_langbianis	0.2	0.74	0.74	0.00
Niviventer_lepturus	2.87	6	0.52	0.00
Niviventer_niviventer	0.53	1.09	0.70	0.00
Niviventer_rapit	0.02	0.05	0.36	0.02
Niviventer_tenaster	0.22	1.08	0.77	0.00
Noctilio_albiventris	0.51	0.99	0.75	0.00
Noctilio_leporinus	0.6	1.17	0.73	0.00
Nomascus_concolor	0.01	0.17	0.70	0.00
Nomascus_gabriellae	0.08	0.79	0.82	0.00
Nomascus_hainanus	0.37	3.38	0.66	0.00
Nomascus_leucogenys	0.05	0.4	0.73	0.00
Nomascus_nasutus	0.1	0.7	0.74	0.00
Nomascus_siki	0	0.14	0.80	0.00
Notiosorex_cockrumi	1.43	1.79	0.52	0.00
Notiosorex_crawfordi	2.24	2.89	0.57	0.00
Notiosorex_evotis	2.86	4.25	0.63	0.00
Notiosorex_villai	0.14	0.27	0.32	0.04
Notomys_alexis	0.02	0.02	0.21	0.19
Notomys_aquilo	0.23	0.49	0.32	0.04
Notomys_cervinus	0	0	0.35	0.03
Notomys_fuscus	0.05	0.04	-0.15	0.35
Notomys_mitchellii	0.03	0.05	0.41	0.01
Notopteris_macdonaldi	0.66	0.8	0.20	0.22
Notopteris_neocaledonica	0.13	0.4	0.55	0.00
Nyctalus_aviator	6.72	9.65	0.84	0.00
Nyctalus_azoreum	4.63	10.11	0.82	0.00
Nyctalus_furvus	5.13	6.35	0.50	0.00
Nyctalus_lasiopterus	4.27	5.91	0.60	0.00
Nyctalus_leisleri	5.54	8.49	0.81	0.00
Nyctalus_montanus	1.53	3.42	0.86	0.00
Nyctalus_noctula	4.46	6.5	0.70	0.00
Nyctalus_plancyi	1.58	4.5	0.83	0.00
Nyctereutes_procyonoides	1.87	3.81	0.87	0.00
Nycteris_arge	0.33	0.4	0.30	0.06
Nycteris_aurita	0.06	0.11	0.61	0.00
Nycteris_gambiensis	0.09	0.25	0.71	0.00
Nycteris_grandis	0.2	0.34	0.71	0.00
Nycteris_hispida	0.09	0.2	0.73	0.00

Nycteris_intermedia	0.06	0.16	0.70	0.00
Nycteris_javanica	4.8	9.56	0.76	0.00
Nycteris_macrotis	0.16	0.25	0.64	0.00
Nycteris_nana	0.07	0.18	0.71	0.00
Nycteris_thebaica	0.29	0.58	0.79	0.00
Nycteris_tragata	0.73	1.83	0.75	0.00
Nycteris_woodi	0.21	0.29	0.27	0.10
Nycticebus_bengalensis	0.64	1.74	0.78	0.00
Nycticebus_coucang	1.33	3.35	0.78	0.00
Nycticebus_javanicus	5.78	10.7	0.73	0.00
Nycticebus_menagensis	0.24	0.58	0.71	0.00
Nycticebus_pygmaeus	0.32	1.85	0.82	0.00
Nycticeinops_schlieffeni	0.13	0.28	0.78	0.00
Nycticeius_cubanus	1.32	3.06	0.55	0.00
Nycticeius_humeralis	8.21	9.97	0.57	0.00
Nyctiellus_lepidus	0.92	2.5	0.59	0.00
Nyctimene_aello	0.04	0.08	0.56	0.00
Nyctimene_albiventer	0.04	0.09	0.52	0.00
Nyctimene_cephalotes	0.18	0.52	0.57	0.00
Nyctimene_certans	0.04	0.1	0.35	0.03
Nyctimene_keasti	0.11	0.31	0.54	0.00
Nyctimene_major	0.04	0.06	0.06	0.74
Nyctimene_minutus	0.02	0.09	0.42	0.01
Nyctimene_rabori	1.55	2.98	0.64	0.00
Nyctimene_robinsoni	0.64	0.97	0.66	0.00
Nyctimene_vizcaccia	0.03	0.06	0.21	0.19
Nyctinomops_aurispinosus	1.64	2.9	0.71	0.00
Nyctinomops_femorosaccus	1.42	2.04	0.64	0.00
Nyctinomops_laticaudatus	0.61	1.2	0.74	0.00
Nyctinomops_macrotis	0.76	1.32	0.72	0.00
Nyctomys_sumichrasti	1.38	2.79	0.66	0.00
Nyctophilus_arnhemensis	0.04	0.06	0.59	0.00
Nyctophilus_bifax	0.23	0.36	0.65	0.00
Nyctophilus_geoffroyi	0.15	0.21	0.55	0.00
Nyctophilus_gouldi	0.87	1.21	0.64	0.00
Nyctophilus_howensis	0	0	-0.12	0.55
Nyctophilus_microtis	0.09	0.12	0.25	0.12
Nyctophilus_nebulosus	2.64	5.39	0.30	0.06
Nyctophilus_walkeri	0.03	0.06	0.62	0.00
Ochotona_alpina	0.09	0.18	0.67	0.00
Ochotona_argentata	0	0	-0.12	0.55
Ochotona_cansus	0.4	1.17	0.79	0.00
Ochotona_collaris	0.18	0.19	-0.03	0.88
Ochotona_curzoniae	0.01	0.08	0.82	0.00
Ochotona_dauurica	0.26	0.83	0.79	0.00
Ochotona_erythrotis	0.06	0.25	0.82	0.00

Ochotona_forresti	0.19	0.37	0.71	0.00
Ochotona_gloveri	0	0.05	0.79	0.00
Ochotona_himalayana	0	0.03	0.64	0.00
Ochotona_hoffmanni	0	0	-0.03	0.92
Ochotona_huangensis	0.82	2.39	0.81	0.00
Ochotona_hyperborea	0.15	0.24	0.54	0.00
Ochotona_iliensis	0	0	-0.05	0.81
Ochotona_koslowi	0	0	-0.21	0.27
Ochotona_ladacensis	0.01	0.04	0.71	0.00
Ochotona_macrotis	0.05	0.2	0.81	0.00
Ochotona_nubrica	0.01	0.05	0.76	0.00
Ochotona_pallasi	0.03	0.09	0.65	0.00
Ochotona_princeps	1.07	1.11	0.03	0.88
Ochotona_pusilla	0.72	0.91	0.30	0.06
Ochotona_roylei	0.03	0.12	0.70	0.00
Ochotona_rufescens	1.34	2.58	0.72	0.00
Ochotona_rutila	0.31	0.44	0.46	0.00
Ochotona_thibetana	0.04	0.32	0.82	0.00
Ochotona_thomasi	0.05	0.24	0.85	0.00
Ochotona_turuchanensis	0.3	0.31	0.00	1.00
Ochrotomys_nuttalli	7.66	9.58	0.52	0.00
Octodon_bridgesi	1.1	2.55	0.79	0.00
Octodon_degus	2.25	5.08	0.87	0.00
Octodon_lunatus	1.6	4.36	0.84	0.00
Octodon_pacificus	0.18	0.85	0.67	0.00
Octodontomys_gliroides	0.09	0.23	0.77	0.00
Octomys_mimax	0.43	1.11	0.76	0.00
Odocoileus_hemionus	1.73	1.95	0.30	0.06
Odocoileus_virginianus	3.49	4.17	0.46	0.00
Oecomys_auyantepui	0.04	0.09	0.68	0.00
Oecomys_bicolor	0.2	0.4	0.73	0.00
Oecomys_catherinae	1.03	2.12	0.78	0.00
Oecomys_concolor	0.29	0.5	0.70	0.00
Oecomys_flavicans	6.15	7.66	0.63	0.00
Oecomys_mamorae	0.19	0.3	0.59	0.00
Oecomys_phaeotis	0.02	0.16	0.75	0.00
Oecomys_rex	0.08	0.19	0.71	0.00
Oecomys_roberti	0.05	0.14	0.76	0.00
Oecomys_rutilus	0.04	0.11	0.70	0.00
Oecomys_speciosus	1.52	2.82	0.71	0.00
Oecomys_superans	0.06	0.12	0.71	0.00
Oecomys_trinitatis	0.7	1.41	0.74	0.00
Oenomys_hypoxanthus	0.11	0.21	0.71	0.00
Oenomys_ornatus	0.19	0.74	0.70	0.00
Okapia_johnstoni	0	0	-0.01	0.95
Oligoryzomys_andinus	0.18	0.46	0.67	0.00

Oligoryzomys_arenalis	0.45	1.08	0.73	0.00
Oligoryzomys_chacoensis	0.1	0.24	0.77	0.00
Oligoryzomys_delticola	0.8	1.4	0.72	0.00
Oligoryzomys_destructor	0.42	1.03	0.76	0.00
Oligoryzomys_eliurus	1.34	2.69	0.77	0.00
Oligoryzomys_flavescens	1.19	2.43	0.79	0.00
Oligoryzomys_fornesi	0.7	1.46	0.73	0.00
Oligoryzomys_fulvescens	0.97	1.76	0.65	0.00
Oligoryzomys_griseolus	2.82	3.82	0.56	0.00
Oligoryzomys_longicaudatus	0.45	1.1	0.79	0.00
Oligoryzomys_magellanicus	0.26	0.24	-0.28	0.09
Oligoryzomys_microtis	0.06	0.14	0.74	0.00
Oligoryzomys_nigripes	1.21	2.49	0.81	0.00
Oligoryzomys_stramineus	0.82	1.52	0.65	0.00
Oligoryzomys_vegetus	3.79	5.34	0.60	0.00
Ondatra_zibethicus	2.91	3.26	0.34	0.03
Onychogalea_fraenata	0.25	0.43	0.54	0.00
Onychogalea_unguifera	0.01	0.02	0.40	0.01
Onychomys_arenicola	0.9	1.4	0.60	0.00
Onychomys_leucogaster	1.73	2.09	0.35	0.03
Onychomys_torridus	2.26	2.83	0.60	0.00
Oreamnos_americanus	0.28	0.28	-0.11	0.49
Oreonax_flavicauda	0.05	0.18	0.59	0.00
Oreotragus_oreotragus	0.16	0.32	0.71	0.00
Ornithorhynchus_anatinus	0.89	1.24	0.65	0.00
Orycteropus_afer	0.13	0.24	0.71	0.00
Oryctolagus_cuniculus	2.75	4.09	0.79	0.00
Oryx_beisa	0.01	0.02	0.49	0.00
Oryx_gazella	0.1	0.19	0.67	0.00
Oryx_leucoryx	0.41	0.88	0.70	0.00
Oryzomys_couesi	2.04	3.37	0.68	0.00
Oryzomys_dimidiatus	0	0	-0.12	0.55
Oryzomys_gorgasi	10.44	10.23	-0.01	0.98
Oryzomys_palustris	8.6	10.47	0.57	0.00
Oryzorictes_hova	0	0.01	0.51	0.00
Osgoodomys_banderanus	2.13	3.22	0.58	0.00
Otocolobus_manul	0.19	0.49	0.77	0.00
Otocyon_megalotis	0.12	0.21	0.64	0.00
Otolemur_crassicaudatus	0.11	0.25	0.73	0.00
Otolemur_garnettii	0.09	0.18	0.61	0.00
Otomops_madagascariensis	0	0	0.06	0.77
Otomops_martiensseni	0.06	0.13	0.69	0.00
Otomys_anchietae	0	0.15	0.72	0.00
Otomys_angoniensis	1.37	2.15	0.74	0.00
Otomys_barbouri	0.04	0	-0.28	0.13
Otomys_burtoni	0.2	0.07	0.20	0.25

Otomys_cuanzensis	0	0.08	0.65	0.00
Otomys_denti	0.13	0.25	0.42	0.01
Otomys_irroratus	2.01	2.99	0.72	0.00
Otomys_lacustris	0.12	0.18	0.26	0.11
Otomys_laminatus	2.93	4.34	0.66	0.00
Otomys_occidentalis	0.02	0.05	0.16	0.32
Otomys_saundersiae	1.18	1.62	0.62	0.00
Otomys_sloggetti	0.53	0.91	0.69	0.00
Otomys_tropicalis	0.3	0.64	0.71	0.00
Otomys_typus	0.06	0.21	0.75	0.00
Otomys_unisulcatus	0.53	0.72	0.56	0.00
Otonycteris_hemprichii	1.27	2.47	0.79	0.00
Otonyctomys_hatti	0.96	1.72	0.62	0.00
Otopteropus_cartilagonodus	1.63	3.03	0.63	0.00
Ototylomys_phyllotis	0.97	2.09	0.63	0.00
Ourebia_ourebi	0.14	0.24	0.73	0.00
Ovibos_moschatus	0.01	0.03	0.31	0.05
Ovis_ammon	0.05	0.17	0.79	0.00
Ovis_canadensis	1	1.15	0.39	0.01
Ovis_dalli	0.04	0.05	0.10	0.57
Ovis_nivicola	0.02	0.02	-0.12	0.45
Ovis_orientalis	1.34	2.55	0.76	0.00
Oxymycterus_amazonicus	0.04	0.14	0.77	0.00
Oxymycterus_angularis	1.55	3.04	0.70	0.00
Oxymycterus_caparoae	3.44	6.31	0.70	0.00
Oxymycterus_dasytrichus	3.23	5.56	0.74	0.00
Oxymycterus_delator	0.63	1.54	0.71	0.00
Oxymycterus_hiska	0.02	0.07	0.35	0.03
Oxymycterus_hispidus	3.37	6.25	0.79	0.00
Oxymycterus_hucucha	0.55	0.04	-0.61	0.00
Oxymycterus_inca	0.13	0.11	-0.18	0.26
Oxymycterus_josei	12.05	18.61	0.61	0.00
Oxymycterus_nasutus	1.15	2.71	0.83	0.00
Oxymycterus_paramensis	0.39	1	0.82	0.00
Oxymycterus_quaestor	1.6	3.57	0.81	0.00
Oxymycterus_roberti	0.68	1.51	0.74	0.00
Oxymycterus_rufus	1.35	2.4	0.78	0.00
Ozotoceros_bezoarticus	0.16	0.41	0.76	0.00
Pachyuromys_duprasi	1.23	2.49	0.79	0.00
Paguma_larvata	1.23	2.88	0.83	0.00
Pan_paniscus	0	0	0.36	0.02
Pan_troglodytes	0.1	0.18	0.57	0.00
Panthera_leo	0.01	0.03	0.60	0.00
Panthera_onca	0.27	0.56	0.73	0.00
Panthera_pardus	0.3	0.78	0.78	0.00
Panthera_tigris	0.12	0.35	0.69	0.00

Panthera_uncia	0.01	0.04	0.72	0.00
Pantholops_hodgsonii	0	0.01	0.53	0.00
Papagomys_armandvillei	0.07	0.24	0.57	0.00
Papio_anubis	0.06	0.13	0.70	0.00
Papio_cynocephalus	0.05	0.14	0.73	0.00
Papio_hamadryas	0.25	0.62	0.72	0.00
Papio_papio	0.04	0.09	0.69	0.00
Papio_ursinus	0.44	0.71	0.70	0.00
Paracrocidura_maxima	0.12	0.31	0.47	0.00
Paracrocidura_schoutedeni	0.08	0.17	0.68	0.00
Paracynictis_selousi	0.12	0.24	0.61	0.00
Paradipus_ctenodactylus	0.36	0.48	0.34	0.03
Paradoxurus_hermaphroditus	1.4	3.25	0.80	0.00
Paradoxurus_jerdoni	1.17	3.57	0.71	0.00
Paradoxurus_zeylonensis	2.11	4.83	0.71	0.00
Paraechinus_aethiopicus	1.25	2.5	0.79	0.00
Paraechinus_hypomelas	0.91	1.81	0.71	0.00
Paraechinus_micropus	1.89	3.44	0.57	0.00
Paraechinus_nudiventris	4.65	9.8	0.89	0.00
Parahydromys_asper	0.04	0.1	0.38	0.02
Paraleptomys_rufilatus	0.04	0.18	0.61	0.00
Paralomys_gerbillus	1.21	2.93	0.74	0.00
Paramelomys_gressitti	0.02	0.07	0.26	0.11
Paramelomys_levipes	0.07	0.11	0.26	0.11
Paramelomys_lorentzii	0	0.03	0.55	0.00
Paramelomys_mollis	0.05	0.11	0.41	0.01
Paramelomys_moncktoni	0.12	0.19	0.29	0.07
Paramelomys_naso	0.01	0.09	0.64	0.00
Paramelomys_platyops	0.05	0.1	0.50	0.00
Paramelomys_rubex	0.05	0.12	0.42	0.01
Parantechinus_apicalis	0	0.02	0.57	0.00
Paranyctimene_raptor	0.04	0.09	0.55	0.00
Paranyctimene_tenax	0.04	0.09	0.55	0.00
Parascalops_breweri	12.48	12.72	0.02	0.93
Paraxerus_alexandri	0.05	0.12	0.58	0.00
Paraxerus_boehmi	0.03	0.07	0.65	0.00
Paraxerus_cepapi	0.13	0.27	0.70	0.00
Paraxerus_flavovittis	0.06	0.12	0.69	0.00
Paraxerus_ochraceus	0.15	0.3	0.64	0.00
Paraxerus_palliatus	0.09	0.2	0.75	0.00
Paraxerus_poensis	0.28	0.47	0.67	0.00
Paraxerus_vexillarius	0.08	0.12	0.23	0.16
Paraxerus_vincenti	0.01	0.09	0.52	0.00
Pardofelis_badia	0	0.01	0.40	0.01
Pardofelis_marmorata	0.14	0.6	0.79	0.00
Pardofelis_temminckii	0.26	1.09	0.78	0.00

Parotomys_brantsii	0.1	0.16	0.47	0.00
Parotomys_littledalei	0.07	0.11	0.47	0.00
Paruromys_dominator	0.21	0.63	0.61	0.00
Pattonomys_semivillosus	2.62	4.3	0.70	0.00
Paulamys_naso	0.19	0.62	0.54	0.00
Pearsonomys_annectens	0.43	0.94	0.69	0.00
Pecari_tajacu	0.7	1.28	0.73	0.00
Pectinator_spekei	0.01	0.04	0.70	0.00
Pedetes_capensis	0.29	0.46	0.71	0.00
Pedetes_surdaster	0.14	0.28	0.67	0.00
Pelea_capreolus	1.43	2.27	0.76	0.00
Pelomys_campanae	0.09	0.26	0.74	0.00
Pelomys_fallax	0.07	0.14	0.63	0.00
Pelomys_minor	0	0.04	0.73	0.00
Pentalagus_furnessi	0.46	0.63	0.14	0.38
Penthetor_lucasi	0.65	1.63	0.75	0.00
Perameles_bougainville	0.01	0.02	0.18	0.28
Perameles_gunnii	0.75	0.99	0.38	0.02
Perameles_nasuta	1.51	2.02	0.65	0.00
Perodicticus_potto	0.35	0.44	0.37	0.02
Perognathus_alticolus	3.97	3.71	-0.10	0.53
Perognathus_amplus	3.21	4.45	0.73	0.00
Perognathus_fasciatus	1.48	1.95	0.33	0.04
Perognathus_flavescens	2.54	3.02	0.34	0.03
Perognathus_flavus	1.55	2.12	0.61	0.00
Perognathus_inornatus	8.77	9.83	0.48	0.00
Perognathus_longimembris	3.18	3.63	0.54	0.00
Perognathus_merriami	2.07	2.85	0.54	0.00
Perognathus_parvus	1.47	1.54	0.18	0.26
Peromyscus_attwateri	4.2	5.41	0.59	0.00
Peromyscus_aztecus	1.79	3.77	0.68	0.00
Peromyscus_beatae	1.62	3.74	0.70	0.00
Peromyscus_boylii	1.72	2.05	0.51	0.00
Peromyscus_bullatus	2.43	6.67	0.66	0.00
Peromyscus_californicus	10.35	10.81	0.31	0.05
Peromyscus_caniceps	0	0	-0.12	0.55
Peromyscus_crinitus	1.03	1.22	0.52	0.00
Peromyscus_dickeyi	0	0	-0.12	0.55
Peromyscus_difficilis	2.44	3.72	0.64	0.00
Peromyscus_eremicus	1.21	1.74	0.62	0.00
Peromyscus_eva	0.49	1.14	0.71	0.00
Peromyscus_fraterculus	4.55	5.18	0.62	0.00
Peromyscus_gossypinus	7.67	9.58	0.60	0.00
Peromyscus_grandis	0.15	0.9	0.63	0.00
Peromyscus_gratus	2.68	4.02	0.64	0.00
Peromyscus_guardia	0	0	-0.12	0.55

Peromyscus_guatemalensis	0.83	3.99	0.79	0.00
Peromyscus_gymnotis	2.32	5.12	0.72	0.00
Peromyscus_hooperi	0.05	0.1	0.47	0.00
Peromyscus_hylocetes	9.83	12.63	0.76	0.00
Peromyscus_interparietalis	0	0	-0.12	0.55
Peromyscus_keeni	0.65	0.61	-0.07	0.69
Peromyscus_leucopus	6.07	7.09	0.47	0.00
Peromyscus_levipes	6.46	9.35	0.70	0.00
Peromyscus_madrensis	0.61	1.39	0.03	0.88
Peromyscus_maniculatus	3.15	3.56	0.33	0.04
Peromyscus_mayensis	0	1.36	0.65	0.00
Peromyscus_megalops	1.35	2.48	0.53	0.00
Peromyscus_mekisturus	2.13	3.54	0.27	0.10
Peromyscus_melanocarpus	0.48	1.07	0.52	0.00
Peromyscus_melanophrys	3.26	4.89	0.66	0.00
Peromyscus_melanotis	3.14	4.69	0.67	0.00
Peromyscus_melanurus	0.2	0.75	0.57	0.00
Peromyscus_merriami	1.21	1.82	0.56	0.00
Peromyscus_mexicanus	1.8	3.34	0.65	0.00
Peromyscus_nasutus	1.38	1.7	0.56	0.00
Peromyscus_ochraventer	0.13	0.25	0.27	0.10
Peromyscus_pectoralis	1.8	2.72	0.66	0.00
Peromyscus_perfulvus	1.4	1.97	0.49	0.00
Peromyscus_polionotus	9.73	12.11	0.62	0.00
Peromyscus_polius	0.22	0.58	0.41	0.01
Peromyscus_pseudocrinitus	0	0	-0.12	0.55
Peromyscus_schmidlyi	0.1	0.21	0.39	0.01
Peromyscus_sejugis	0	0	-0.12	0.55
Peromyscus_simulus	2.41	3.49	0.43	0.01
Peromyscus_slevini	0	0	-0.12	0.55
Peromyscus_spicilegus	0.85	1.28	0.51	0.00
Peromyscus_stephani	0	0	-0.12	0.55
Peromyscus_stirtoni	0.56	1.9	0.63	0.00
Peromyscus_truei	2.42	2.65	0.36	0.02
Peromyscus_winkelmanni	0.87	0.91	-0.05	0.79
Peromyscus_yucatanicus	0.94	1.68	0.61	0.00
Peromyscus_zarhynchus	2.41	5.92	0.78	0.00
Peropteryx_kappleri	0.48	0.93	0.72	0.00
Peropteryx_leucoptera	0.18	0.39	0.73	0.00
Peropteryx_macrotis	0.54	1.05	0.73	0.00
Peroryctes_broadbenti	0.22	0.33	0.28	0.09
Peroryctes_raffrayana	0.07	0.14	0.47	0.00
Petaurista_alborufus	0.85	2.29	0.81	0.00
Petaurista_elegans	0.68	1.9	0.81	0.00
Petaurista_leucogenys	12.89	14.52	0.56	0.00
Petaurista_magnificus	0.09	0.25	0.70	0.00

Petaurista_nobilis	0.04	0.35	0.76	0.00
Petaurista_petaurista	0.74	2.03	0.81	0.00
Petaurista_philippensis	0.64	2.1	0.81	0.00
Petaurista_xanthotis	0.12	0.49	0.82	0.00
Petauroides_volans	0.78	1.08	0.66	0.00
Petaurus_abidi	0	0	-0.12	0.55
Petaurus_australis	1.1	1.44	0.63	0.00
Petaurus_biacensis	0.41	0.47	0.21	0.19
Petaurus_breviceps	0.32	0.46	0.61	0.00
Petaurus_gracilis	0.43	0.6	0.22	0.17
Petaurus_norfolcensis	0.69	0.96	0.66	0.00
Petinomys_crinitus	0.28	0.89	0.34	0.03
Petinomys_fuscocapillus	1.76	4.83	0.73	0.00
Petinomys_genibarbis	0.96	2.27	0.78	0.00
Petinomys_lugens	0.01	0.02	-0.05	0.81
Petinomys_mindanensis	0.28	0.91	0.34	0.03
Petinomys_setosus	0.85	1.93	0.70	0.00
Petinomys_vordermanni	1.43	4.1	0.88	0.00
Petrodromus_tetradactylus	0.06	0.13	0.68	0.00
Petrogale_assimilis	0.07	0.1	0.52	0.00
Petrogale_brachyotis	0.03	0.04	0.30	0.06
Petrogale_burbidgei	0	0	-0.12	0.55
Petrogale_coenensis	0	0	0.24	0.14
Petrogale_godmani	0.05	0.02	-0.09	0.61
Petrogale_herberti	0.22	0.43	0.62	0.00
Petrogale_inornata	0.39	0.82	0.70	0.00
Petrogale_lateralis	0.12	0.1	-0.36	0.02
Petrogale_mareeba	0.07	0.12	0.41	0.01
Petrogale_penicillata	0.56	0.82	0.51	0.00
Petrogale_persephone	0.29	0.5	0.42	0.01
Petrogale_purpureicollis	0.05	0.09	0.54	0.00
Petrogale_rothschildi	0.06	0.22	0.71	0.00
Petrogale_sharmani	0	0	0.07	0.69
Petrogale_xanthopus	0	0	0.32	0.04
Petromus_typicus	0.05	0.08	0.47	0.00
Petromyscus_barbouri	0.12	0.15	0.15	0.35
Petromyscus_collinus	0.03	0.06	0.48	0.00
Petromyscus_monticularis	0.05	0.09	0.53	0.00
Petromyscus_shortridgei	0.04	0.05	0.22	0.17
Petropseudes_dahli	0.03	0.04	0.31	0.05
Phacochoerus_aethiopicus	0.01	0.03	0.54	0.00
Phacochoerus_africanus	0.09	0.21	0.73	0.00
Phaenomys_ferrugineus	7.24	10.67	0.66	0.00
Phaiomys_leucurus	0.01	0.06	0.74	0.00
Phalanger_alexandrae	0.71	0.21	-0.45	0.00
Phalanger_carmelitae	0.04	0.09	0.33	0.04

Phalanger_gymnotis	0.05	0.11	0.55	0.00
Phalanger_intercastellanus	0.16	0.21	0.14	0.38
Phalanger_lullulae	0	0	0.03	0.92
Phalanger_matabiru	2.15	5.65	0.66	0.00
Phalanger_matanim	0	0	-0.12	0.55
Phalanger_mimicus	0.02	0.06	0.34	0.03
Phalanger_orientalis	0.06	0.1	0.44	0.01
Phalanger_ornatus	0.03	0.12	0.45	0.00
Phalanger_rothschildi	0	0.11	0.14	0.43
Phalanger_sericeus	0.05	0.1	0.32	0.04
Phalanger_vestitus	0.07	0.13	0.30	0.07
Phaner_electromontis	0.01	0.05	0.45	0.00
Phaner_furcifer	0	0.01	0.42	0.01
Phaner_pallescens	0.01	0.02	0.40	0.01
Phaner_parienti	0	0.02	0.27	0.09
Pharotis_imogene	0	0	-0.12	0.55
Phascogale_calura	0.04	0.06	0.33	0.04
Phascogale_pirata	0.04	0.07	0.35	0.03
Phascogale_tapoatafa	0.9	1.32	0.64	0.00
Phascolarctos_cinereus	0.66	0.89	0.64	0.00
Phascolosorex_doriae	0.02	0.06	0.63	0.00
Phascolosorex_dorsalis	0.04	0.07	0.44	0.01
Phataginus_tricuspis	0.25	0.33	0.47	0.00
Phenacomys_intermedius	2.14	2.15	0.03	0.88
Phenacomys_ungava	0.57	0.57	-0.08	0.65
Philander_andersoni	0.06	0.11	0.62	0.00
Philander_deltae	0.14	0.31	0.56	0.00
Philander_frenatus	1.86	3.77	0.79	0.00
Philander_mcilhennyi	0.02	0.05	0.50	0.00
Philander_mondolfii	1.06	1.65	0.56	0.00
Philander_opossum	0.31	0.57	0.68	0.00
Philantomba_maxwellii	0.97	1.11	0.14	0.38
Philantomba_monticola	0.09	0.19	0.75	0.00
Philetor_brachypterus	0.39	1.09	0.80	0.00
Phloeomys_cumingi	1.13	2.59	0.55	0.00
Phloeomys_pallidus	0.2	0.47	0.57	0.00
Phodopus_campbelli	0.2	0.71	0.79	0.00
Phodopus_roborovskii	0.21	0.67	0.80	0.00
Phodopus_sungorus	1.01	1.17	0.12	0.45
Phoniscus_atrox	2.14	6.78	0.92	0.00
Phoniscus_jagorii	0.76	1.65	0.70	0.00
Phoniscus_papuensis	0.99	1.34	0.65	0.00
Phylloderma_stenops	0.54	1.04	0.72	0.00
Phyllomys_blainvillii	1	1.95	0.63	0.00
Phyllomys_brasiliensis	0.15	0.49	0.55	0.00
Phyllomys_dasythrix	1.03	2.28	0.81	0.00

Phyllomys_lundi	5.5	7.96	0.51	0.00
Phyllomys_mantiqueirensis	0.51	1.57	0.41	0.01
Phyllomys_medius	2.69	5.43	0.82	0.00
Phyllomys_nigrispinus	5.06	8.5	0.80	0.00
Phyllomys_pattoni	2.34	4.33	0.74	0.00
Phyllomys_thomasi	3.15	6.3	0.56	0.00
Phyllomys_unicolor	0.31	1.17	0.59	0.00
Phyllonycteris_aphylla	9.65	14.16	0.61	0.00
Phyllonycteris_poeyi	1.13	2.66	0.56	0.00
Phyllops_falcatus	1.14	2.67	0.56	0.00
Phyllostomus_discolor	0.54	1.05	0.72	0.00
Phyllostomus_elongatus	0.42	0.83	0.74	0.00
Phyllostomus_hastatus	0.51	1	0.74	0.00
Phyllostomus_latifolius	0.08	0.14	0.59	0.00
Phyllotis_amicus	0.95	2.11	0.71	0.00
Phyllotis_andium	0.23	0.93	0.75	0.00
Phyllotis_bonariensis	0.01	0.19	0.74	0.00
Phyllotis_caprinus	0.12	0.32	0.76	0.00
Phyllotis_darwini	1.35	3.19	0.82	0.00
Phyllotis_definitus	0.39	1.52	0.70	0.00
Phyllotis_haggardi	2.43	6.02	0.87	0.00
Phyllotis_limatus	1.02	1.93	0.74	0.00
Phyllotis_magister	0.21	0.55	0.74	0.00
Phyllotis_osilae	0.15	0.4	0.77	0.00
Phyllotis_wolffsohni	0.26	0.68	0.81	0.00
Phyllotis_xanthopygus	0.17	0.4	0.73	0.00
Pipanacoctomys_aureus	0	0	-0.16	0.42
Pipistrellus_abramus	2.89	5.94	0.85	0.00
Pipistrellus_adamsi	0.05	0.08	0.53	0.00
Pipistrellus_alaschanicus	1.91	4.66	0.86	0.00
Pipistrellus_anchietae	0.27	0.52	0.68	0.00
Pipistrellus_angulatus	0.05	0.08	0.38	0.02
Pipistrellus_brunneus	0.2	0.54	0.70	0.00
Pipistrellus_cadornae	0.29	1.3	0.76	0.00
Pipistrellus_capensis	0.23	0.35	0.66	0.00
Pipistrellus_ceylonicus	2.2	4.99	0.76	0.00
Pipistrellus_collinus	0.05	0.11	0.37	0.02
Pipistrellus_coromandra	1.36	3.09	0.79	0.00
Pipistrellus_crassulus	0.05	0.1	0.67	0.00
Pipistrellus_deserti	0.23	0.5	0.75	0.00
Pipistrellus_endoi	7.51	8.88	0.47	0.00
Pipistrellus_guineensis	0.11	0.23	0.71	0.00
Pipistrellus_hesperidus	0.23	0.43	0.67	0.00
Pipistrellus_hesperus	1.91	2.41	0.58	0.00
Pipistrellus_imbricatus	3.2	6.45	0.74	0.00
Pipistrellus_javanicus	0.93	2.34	0.80	0.00

Pipistrellus_kuhlii	2.7	4.49	0.80	0.00
Pipistrellus_maderensis	11.53	16.43	0.77	0.00
Pipistrellus_murrayi	0	18.05	0.60	0.00
Pipistrellus_nanulus	0.44	0.76	0.70	0.00
Pipistrellus_nanus	0.15	0.29	0.74	0.00
Pipistrellus_nathusii	5.91	8.45	0.70	0.00
Pipistrellus_papuanus	0.06	0.1	0.32	0.04
Pipistrellus_paterculus	0.29	1.08	0.79	0.00
Pipistrellus_pipistrellus	3.87	6.35	0.84	0.00
Pipistrellus_pulveratus	0.88	2.88	0.85	0.00
Pipistrellus_pygmaeus	6.97	11	0.79	0.00
Pipistrellus_rendalli	0.07	0.15	0.69	0.00
Pipistrellus_rueppellii	0.31	0.65	0.80	0.00
Pipistrellus_rusticus	0.11	0.26	0.74	0.00
Pipistrellus_savii	3.2	5.31	0.84	0.00
Pipistrellus_somalicus	0.04	0.1	0.64	0.00
Pipistrellus_stenopterus	0.98	2.41	0.75	0.00
Pipistrellus_subflavus	8	9.34	0.51	0.00
Pipistrellus_tenuipinnis	0.33	0.42	0.38	0.02
Pipistrellus_tenuis	1.55	3.49	0.78	0.00
Pipistrellus_wattsi	0.44	0.64	0.30	0.06
Pipistrellus_westralis	0.06	0.11	0.70	0.00
Pipistrellus_zuluensis	0.38	0.64	0.68	0.00
Pithecheir_melanurus	5.7	10.9	0.71	0.00
Pithecia_aequatorialis	0.36	0.64	0.71	0.00
Pithecia_albicans	0.08	0.14	0.25	0.12
Pithecia_irrorata	0.03	0.12	0.76	0.00
Pithecia_monachus	0.04	0.07	0.45	0.00
Pithecia_pithecia	0.07	0.16	0.76	0.00
Plagiodontia_aedium	0.29	0.78	0.52	0.00
Planigale_gilesi	0.02	0.03	0.35	0.03
Planigale_ingrami	0.01	0.02	0.49	0.00
Planigale_maculata	0.25	0.4	0.64	0.00
Planigale_novaeguineae	0.07	0.12	0.35	0.03
Planigale_tenuirostris	0.02	0.04	0.43	0.01
Platacanthomys_lasiurus	1.61	3.88	0.69	0.00
Platalina_genovensium	0.67	1.48	0.77	0.00
Platymops_setiger	0.17	0.39	0.61	0.00
Platyrrhinus_albericoi	0.27	0.43	0.61	0.00
Platyrrhinus_aurarius	0.02	0.04	0.49	0.00
Platyrrhinus_brachycephalus	0.11	0.22	0.76	0.00
Platyrrhinus_chocoensis	0.17	0.45	0.63	0.00
Platyrrhinus_dorsalis	2.35	3.58	0.64	0.00
Platyrrhinus_helleri	0.47	0.9	0.73	0.00
Platyrrhinus_infuscus	0.35	0.61	0.60	0.00
Platyrrhinus_ismaeli	0.54	1.06	0.72	0.00

Platyrrhinus_lineatus	0.83	1.7	0.77	0.00
Platyrrhinus_masu	0.01	0.09	0.74	0.00
Platyrrhinus_matapalensis	1.76	4.16	0.74	0.00
Platyrrhinus_nigellus	0.39	0.91	0.74	0.00
Platyrrhinus_recifinus	1.49	2.95	0.76	0.00
Platyrrhinus_vittatus	2.28	3.54	0.62	0.00
Plecotus_auritus	5.67	8.19	0.71	0.00
Plecotus_austriacus	7.11	11.31	0.85	0.00
Plecotus_kolombatovici	2.53	5.24	0.83	0.00
Plecotus_macrobullaris	2.83	5.16	0.79	0.00
Plecotus_ognevi	0.61	1.12	0.74	0.00
Plecotus_sacrimontis	13.21	14.73	0.40	0.01
Plecotus_sardus	5.69	9.4	0.85	0.00
Plecotus_taivanus	13.29	18.89	0.82	0.00
Plecotus_teneriffae	11.82	16.08	0.72	0.00
Podogymnura_aureospinula	0.02	0.11	0.31	0.05
Podogymnura_truei	0	0.11	0.24	0.13
Podomys_floridanus	18.37	21.74	0.72	0.00
Podoxymys_roraimae	0	0.01	0.06	0.76
Poecilogale_albinucha	0.26	0.45	0.70	0.00
Poelagus_marjorita	0	0.02	0.42	0.01
Pogonomelomys_brassi	0	0.01	0.21	0.19
Pogonomelomys_bruijnii	0.39	0.35	-0.12	0.45
Pogonomelomys_mayeri	0.04	0.1	0.70	0.00
Pogonomys_fergussoniensis	0	0	-0.18	0.30
Pogonomys_loriae	0.06	0.11	0.36	0.02
Pogonomys_macrourus	0.07	0.14	0.41	0.01
Pogonomys_sylvestris	0.04	0.09	0.39	0.01
Poiana_richardsonii	0.05	0.11	0.69	0.00
Pongo_abelii	0	0.04	0.44	0.01
Pongo_pygmaeus	0.02	0.01	0.01	0.98
Porcula_salvania	0	0.05	0.39	0.02
Potamochoerus_larvatus	0.11	0.23	0.70	0.00
Potamochoerus_porcus	0.28	0.34	0.38	0.02
Potamogale_velox	0.05	0.11	0.73	0.00
Potorous_gilbertii	0	0	-0.12	0.55
Potorous_longipes	0.02	0.04	0.21	0.19
Potorous_tridactylus	0.81	1.17	0.53	0.00
Potos_flavus	0.54	1.04	0.71	0.00
Praomys_daltoni	0.12	0.26	0.71	0.00
Praomys_degraaffi	0	0.01	0.21	0.27
Praomys_delectorum	0.23	0.39	0.58	0.00
Praomys_derooi	0.58	1.13	0.71	0.00
Praomys_hartwigi	0.07	0.2	0.26	0.11
Praomys_jacksoni	0.06	0.12	0.72	0.00
Praomys_lukolelae	0	0	0.34	0.03

Praomys_misonnei	0	0	0.15	0.37
Praomys_morio	1.06	0.4	-0.49	0.00
Praomys_obscurus	0.06	0.35	0.25	0.12
Praomys_petteri	0.09	0.35	0.72	0.00
Praomys_rostratus	0.23	0.61	0.70	0.00
Praomys_tullbergi	1.32	1.49	0.12	0.45
Presbytis_chrysomelas	0.36	0.82	0.74	0.00
Presbytis_comata	3.3	6.97	0.61	0.00
Presbytis_femoralis	2	5.26	0.86	0.00
Presbytis_frontata	0.1	0.29	0.68	0.00
Presbytis_hosei	0.31	0.71	0.70	0.00
Presbytis_melalophos	0.8	1.55	0.52	0.00
Presbytis_natunae	0.03	0.29	0.50	0.00
Presbytis_potenziani	0	0	-0.16	0.33
Presbytis_rubicunda	0.2	0.49	0.68	0.00
Presbytis_siamensis	1.7	5.3	0.91	0.00
Presbytis_thomasi	0.83	0.94	0.06	0.74
Priodontes_maximus	0.2	0.41	0.76	0.00
Prionailurus_bengalensis	1.26	3.01	0.80	0.00
Prionailurus_planiceps	0.09	0.32	0.64	0.00
Prionailurus_rubiginosus	2.55	5.64	0.73	0.00
Prionailurus_viverrinus	0.22	0.84	0.71	0.00
Prionodon_linsang	0.69	1.76	0.75	0.00
Prionodon_pardicolor	0.27	1.14	0.79	0.00
Procapra_gutturosa	0.01	0.05	0.70	0.00
Procapra_picticaudata	0.02	0.11	0.83	0.00
Procapra_przewalskii	0	0.12	0.72	0.00
Procavia_capensis	0.29	0.55	0.78	0.00
Procolobus_badius	0.16	0.53	0.70	0.00
Procolobus_gordonorum	0.01	0.01	-0.26	0.11
Procolobus_kirkii	0.88	2.39	0.69	0.00
Procolobus_pennantii	1.43	1.87	-0.01	0.98
Procolobus_preussi	0	0	-0.12	0.55
Procolobus_rufomitratus	0	0.01	0.33	0.04
Procolobus_verus	0.39	0.83	0.64	0.00
Procyon_cancrivorus	0.48	0.98	0.78	0.00
Procyon_lotor	4.31	5.06	0.45	0.00
Procyon_pygmaeus	5.35	6.35	0.34	0.03
Proechimys_brevicauda	0.07	0.15	0.74	0.00
Proechimys_canicollis	2.76	3.87	0.42	0.01
Proechimys_cuvieri	0.07	0.15	0.79	0.00
Proechimys_decumanus	3.39	6.69	0.77	0.00
Proechimys_echinothrix	0.02	0.04	0.25	0.12
Proechimys_goeldii	0.06	0.17	0.75	0.00
Proechimys_guairae	3.96	5.37	0.65	0.00
Proechimys_guyannensis	0.07	0.14	0.70	0.00

Proechimys_longicaudatus	0.17	0.39	0.71	0.00
Proechimys_pattoni	0.01	0.05	0.71	0.00
Proechimys_poliopus	0.84	0.88	-0.11	0.49
Proechimys_quadruplicatus	0.06	0.1	0.64	0.00
Proechimys_roberti	0.18	0.46	0.77	0.00
Proechimys_semispinosus	0.69	1.71	0.71	0.00
Proechimys_simonsi	0.06	0.12	0.69	0.00
Proechimys_steerei	0.02	0.08	0.74	0.00
Proechimys_urichi	4.2	10.85	0.71	0.00
Proedromys_bedfordi	0	0.23	0.74	0.00
Prolemur_simus	0	0	-0.12	0.55
Prometheomys_schaposchnikowi	0.09	0.58	0.70	0.00
Promops_centralis	0.77	1.35	0.67	0.00
Promops_nasutus	0.24	0.54	0.76	0.00
Pronolagus_crassicaudatus	1.2	2.1	0.65	0.00
Pronolagus_randensis	0.91	1.39	0.70	0.00
Pronolagus_rupestris	0.13	0.22	0.53	0.00
Pronolagus_saundersiae	0.62	1.07	0.67	0.00
Propithecus_candidus	0	0	-0.12	0.55
Propithecus_coquereli	0	0	-0.12	0.47
Propithecus_coronatus	0	0	-0.23	0.21
Propithecus_deckenii	0	0	-0.04	0.83
Propithecus_diadema	0	0.02	0.36	0.03
Propithecus_edwardsi	0	0	-0.23	0.21
Propithecus_perrieri	0	0	-0.12	0.55
Propithecus_tattersalli	0	0	-0.10	0.62
Propithecus_verreauxi	0.01	0.01	0.34	0.03
Prosciurillus_rosenbergii	0.38	0.76	0.26	0.11
Proteles_cristata	0.26	0.43	0.69	0.00
Protochromys_fellowsi	0.08	0.14	0.26	0.11
Protoxerus_stangeri	0.4	0.5	0.35	0.03
Przewalskium_albirostris	0.01	0.07	0.74	0.00
Psammomys_obesus	1.07	2.15	0.78	0.00
Pseudalopex_culpaeus	0.28	0.69	0.77	0.00
Pseudalopex_fulvipes	0.11	0.53	0.79	0.00
Pseudalopex_griseus	0.31	0.73	0.77	0.00
Pseudalopex_gymnocercus	0.55	1.16	0.79	0.00
Pseudalopex_sechurae	1.88	3.73	0.79	0.00
Pseudalopex_vetulus	0.52	1.16	0.75	0.00
Pseudantechinus_bilarni	0.01	0.01	-0.07	0.69
Pseudantechinus_macdonnellensis	0.01	0.01	-0.23	0.16
Pseudantechinus_mimulus	0.62	0.77	0.50	0.00
Pseudantechinus_ningbing	0.01	0.01	0.03	0.88
Pseudantechinus_roryi	0.05	0.14	0.71	0.00
Pseudantechinus_woolleyae	0.04	0.1	0.57	0.00
Pseudocheirus_occidentalis	0.34	0.61	0.40	0.01

Pseudocheirus_peregrinus	0.94	1.29	0.64	0.00
Pseudochirops_albertisii	0.18	0.2	-0.01	0.98
Pseudochirops_archeri	0.05	0.14	0.30	0.06
Pseudochirops_corinnae	0.04	0.1	0.44	0.01
Pseudochirops_coronatus	0	0	-0.27	0.16
Pseudochirops_cupreus	0.05	0.11	0.37	0.02
Pseudochirulus_canescens	0.06	0.11	0.50	0.00
Pseudochirulus_caroli	0.03	0.09	0.65	0.00
Pseudochirulus_cinereus	0	0	-0.24	0.17
Pseudochirulus_forbesi	0.01	0.04	0.41	0.01
Pseudochirulus_herbertensis	0.03	0.11	0.32	0.04
Pseudochirulus_larvatus	0.07	0.14	0.32	0.04
Pseudochirulus_mayeri	0.04	0.1	0.46	0.00
Pseudochirulus_schlegeli	0	0	-0.27	0.16
Pseudohydromys_ellermani	0.06	0.13	0.36	0.02
Pseudohydromys_fuscus	0.07	0.14	0.34	0.03
Pseudohydromys_murinus	0.06	0.12	0.33	0.04
Pseudois_nayaur	0.04	0.17	0.78	0.00
Pseudois_schaeferi	0	0.04	0.73	0.00
Pseudomys_albocinereus	0.05	0.08	0.44	0.01
Pseudomys_apodemoides	0.03	0.05	0.29	0.07
Pseudomys_australis	0.02	0.03	0.54	0.00
Pseudomys_bolami	0.04	0.05	0.28	0.09
Pseudomys_calabyi	0.03	0.08	0.36	0.02
Pseudomys_chapmani	0.04	0.13	0.70	0.00
Pseudomys_delicatulus	0.15	0.25	0.66	0.00
Pseudomys_desertor	0.02	0.04	0.52	0.00
Pseudomys_fieldi	0	0	-0.12	0.55
Pseudomys_fumeus	0.01	0.02	0.12	0.45
Pseudomys_gracilicaudatus	0.67	1.07	0.70	0.00
Pseudomys_hermannsburgensis	0.02	0.03	0.44	0.01
Pseudomys_higginsi	0.4	0.54	0.36	0.02
Pseudomys_johnsoni	0.01	0.02	0.47	0.00
Pseudomys_nanus	0.02	0.03	0.44	0.01
Pseudomys_novaehollandiae	0.71	0.92	0.45	0.00
Pseudomys_occidentalis	0.01	0.04	0.39	0.01
Pseudomys_oralis	0.07	0.04	0.06	0.74
Pseudomys_patrius	0.18	0.36	0.60	0.00
Pseudomys_shortridgei	0.05	0.1	0.51	0.00
Pseudoryx_nghetinhensis	0	0.07	0.82	0.00
Pseudoryzomys_simplex	0.33	0.78	0.78	0.00
Ptenochirus_jagori	0.92	1.85	0.62	0.00
Ptenochirus_minor	0.34	0.9	0.46	0.00
Pteralopex_anceps	0.01	0.03	0.25	0.12
Pteralopex_atrata	0.15	0.27	0.31	0.05
Pteralopex_flanneryi	0.01	0.01	-0.04	0.83

Pteralopex_pulchra	0	0	-0.05	0.82
Pteralopex_taki	0.02	0.02	-0.19	0.24
Pteromys_momonga	9.2	10.95	0.48	0.00
Pteromys_volans	0.84	1.17	0.61	0.00
Pteromyscus_pulverulentus	0.82	2.24	0.82	0.00
Pteronotus_davyi	1.25	2.1	0.65	0.00
Pteronotus_gymnonotus	0.68	1.24	0.65	0.00
Pteronotus_macleayii	1.51	3.18	0.57	0.00
Pteronotus_paraguanensis	6.2	9.55	0.68	0.00
Pteronotus_parnellii	0.71	1.2	0.70	0.00
Pteronotus_personatus	1.03	1.75	0.69	0.00
Pteronotus_quadridens	2.77	4.38	0.60	0.00
Pteronura_brasiliensis	0.16	0.39	0.77	0.00
Pteropus_admiralitatum	0.03	0.06	0.21	0.19
Pteropus_aldabrensis	0	0	-0.12	0.55
Pteropus_alecto	0.25	0.44	0.68	0.00
Pteropus_anetianus	0.26	0.36	0.10	0.53
Pteropus_aruensis	0.01	0	-0.18	0.26
Pteropus_caniceps	0.05	0.17	0.45	0.00
Pteropus_capistratus	0.03	0.05	0.13	0.41
Pteropus_chrysoproctus	0.09	0.18	0.39	0.01
Pteropus_cognatus	0	0	-0.12	0.55
Pteropus_conspicillatus	0.17	0.31	0.55	0.00
Pteropus_dasymallus	13.39	18.46	0.82	0.00
Pteropus_faunulus	0.29	0.61	0.36	0.02
Pteropus_fundatus	0	0	-0.18	0.35
Pteropus_giganteus	2.22	4.55	0.74	0.00
Pteropus_hypomelanus	0.57	1.21	0.65	0.00
Pteropus_insularis	4.28	2.13	-0.56	0.00
Pteropus_leucopterus	0.63	1.51	0.64	0.00
Pteropus_livingstonii	0.26	0.42	0.33	0.04
Pteropus_lylei	4.72	9.22	0.84	0.00
Pteropus_macrotis	0.04	0.08	0.52	0.00
Pteropus_mahaganus	0.01	0.01	-0.06	0.74
Pteropus_mariannus	22.41	20.83	-0.26	0.11
Pteropus_melanopogon	0.1	0.31	0.52	0.00
Pteropus_melanotus	0.41	0.82	0.64	0.00
Pteropus_molossinus	1.93	1.72	-0.13	0.41
Pteropus_neohibernicus	0.03	0.08	0.56	0.00
Pteropus_niger	8.86	16.19	0.81	0.00
Pteropus_nitendiensis	0	0	-0.12	0.55
Pteropus_ocularis	0.1	0.19	0.37	0.02
Pteropus_ornatus	0.39	0.92	0.70	0.00
Pteropus_pelewensis	0.94	0.91	-0.06	0.74
Pteropus_personatus	0.04	0.17	0.48	0.00
Pteropus_pohlei	0.03	0.15	0.51	0.00

Pteropus_poliocephalus	2.35	3.01	0.65	0.00
Pteropus_pselaphon	3.04	3.03	-0.15	0.35
Pteropus_pumilus	0.71	1.52	0.58	0.00
Pteropus_rayneri	0.03	0.06	0.30	0.06
Pteropus_rennelli	0	0	-0.12	0.55
Pteropus_rodricensis	3.05	7.5	0.78	0.00
Pteropus_rufus	0.04	0.06	0.50	0.00
Pteropus_samoensis	0.77	0.93	0.21	0.19
Pteropus_scapulatus	0.22	0.31	0.61	0.00
Pteropus_seychellensis	1.19	3.22	0.74	0.00
Pteropus_temminckii	0.1	0.19	0.37	0.02
Pteropus_tonganus	0.47	0.72	0.46	0.00
Pteropus_tuberculatus	0	0	-0.10	0.63
Pteropus_ualanus	1.72	2.09	0.11	0.49
Pteropus_vampyrus	1.13	2.63	0.78	0.00
Pteropus_vetulus	0.42	0.98	0.70	0.00
Pteropus_voeltzkowi	0.41	0.52	0.17	0.29
Pteropus_woodfordi	0.06	0.11	0.26	0.11
Pteropus_yapensis	2.57	3.61	0.30	0.07
Ptilocercus_lowii	1.49	3.96	0.85	0.00
Pudu_mephistophiles	1.05	2.46	0.78	0.00
Pudu_puda	0.34	0.91	0.76	0.00
Puma_concolor	0.94	1.41	0.67	0.00
Puma_yagouaroundi	0.56	1.12	0.73	0.00
Punomys_kofordi	0	0	-0.12	0.55
Punomys_lemminus	0	0.09	0.56	0.00
Pygathrix_cinerea	0.05	1.02	0.83	0.00
Pygathrix_nemaeus	0.05	0.51	0.77	0.00
Pygathrix_nigripes	0.07	1.36	0.79	0.00
Pygeretmus_platyurus	0.15	0.37	0.60	0.00
Pygeretmus_pumilio	0.4	0.78	0.70	0.00
Pygeretmus_zhitkovi	0.22	0.3	0.24	0.14
Pygoderma_bilabiatum	1.1	2.28	0.79	0.00
Rangifer_tarandus	0.27	0.32	0.54	0.00
Raphicerus_campestris	0.32	0.54	0.69	0.00
Raphicerus_melanotis	1.09	1.52	0.62	0.00
Raphicerus_sharpei	0.11	0.2	0.57	0.00
Rattus_andamanensis	0.31	1.11	0.79	0.00
Rattus_annandalei	2.67	7.47	0.88	0.00
Rattus_argentiventer	1.07	2.63	0.79	0.00
Rattus_arrogans	0.09	0.28	0.72	0.00
Rattus_baluensis	0.99	0.95	-0.10	0.53
Rattus_burrus	0.04	0.2	0.48	0.00
Rattus_colletti	0.12	0.21	0.67	0.00
Rattus_elaphinus	0.05	0.01	-0.68	0.00
Rattus_everetti	0.89	1.78	0.59	0.00

Rattus_exulans	0.66	1.62	0.75	0.00
Rattus_feliceus	0.03	0.12	0.43	0.01
Rattus_fuscipes	1.3	1.73	0.62	0.00
Rattus_hainaldi	0.19	0.62	0.54	0.00
Rattus_hoffmanni	0.21	0.63	0.61	0.00
Rattus_hoogerwerfi	0	0	-0.30	0.10
Rattus_jobiensis	0.23	0.33	0.31	0.05
Rattus_leucopus	0.06	0.12	0.50	0.00
Rattus_losea	1.59	4.41	0.87	0.00
Rattus_lugens	0	0	-0.15	0.36
Rattus_lutreolus	1.85	2.39	0.63	0.00
Rattus_marmosurus	0.15	0.51	0.53	0.00
Rattus_mollicomulus	0.08	0.37	0.30	0.07
Rattus_montanus	2.95	5.32	0.61	0.00
Rattus_mordax	0.2	0.27	0.20	0.22
Rattus_morotaiensis	0.03	0.12	0.45	0.00
Rattus_niobe	0.07	0.14	0.34	0.03
Rattus_nitidus	0.89	2.84	0.84	0.00
Rattus_norvegicus	3	4.31	0.72	0.00
Rattus_novaeguineae	0.11	0.27	0.30	0.06
Rattus_osgoodi	0.21	2.58	0.83	0.00
Rattus_palmarum	0.18	0.38	0.40	0.01
Rattus_pococki	0.02	0.06	0.65	0.00
Rattus_praetor	0.05	0.1	0.57	0.00
Rattus_pyctoris	0.5	0.9	0.71	0.00
Rattus_ranjiniae	31.94	43.17	0.64	0.00
Rattus_rattus	2.19	3.92	0.84	0.00
Rattus_richardsoni	0.14	0.4	0.71	0.00
Rattus_satarae	2.15	5.6	0.69	0.00
Rattus_simalurensis	0	0.11	0.67	0.00
Rattus_sordidus	0.19	0.3	0.53	0.00
Rattus_steini	0.04	0.09	0.36	0.02
Rattus_stoicus	1.2	2.74	0.70	0.00
Rattus_tanezumi	1.77	3.78	0.85	0.00
Rattus_tiomanicus	1.09	2.53	0.77	0.00
Rattus_tunneyi	0.32	0.5	0.65	0.00
Rattus_vandeuseni	0	0	-0.12	0.55
Rattus_verecundus	0.05	0.11	0.34	0.03
Rattus_villosissimus	0.01	0.02	0.26	0.11
Rattus_xanthurus	0.59	1.71	0.50	0.00
Ratufa_affinis	0.72	1.8	0.75	0.00
Ratufa_bicolor	0.65	2.02	0.80	0.00
Ratufa_indica	1.9	3.91	0.67	0.00
Ratufa_macroura	3.35	7.45	0.78	0.00
Redunca_arundinum	0.15	0.32	0.75	0.00
Redunca_fulvorufula	0.83	1.47	0.74	0.00

Redunca_redunca	0.05	0.13	0.70	0.00
Reithrodon_auritus	0.49	0.82	0.73	0.00
Reithrodon_typicus	0.79	1.49	0.70	0.00
Reithrodontomys_bakeri	0.39	0.86	0.30	0.07
Reithrodontomys_brevirostris	2.97	4.55	0.67	0.00
Reithrodontomys_chrysopsis	9.58	12.39	0.72	0.00
Reithrodontomys_creper	4.18	5.8	0.62	0.00
Reithrodontomys_darienensis	0.99	2.19	0.78	0.00
Reithrodontomys_fulvescens	3.11	4.33	0.61	0.00
Reithrodontomys_gracilis	1.14	2.49	0.65	0.00
Reithrodontomys_hirsutus	1	1.55	0.48	0.00
Reithrodontomys_humulis	8.48	10.56	0.56	0.00
Reithrodontomys_megalotis	2.68	3.27	0.50	0.00
Reithrodontomys_mexicanus	1.6	3.06	0.69	0.00
Reithrodontomys_microdon	6.92	10.49	0.77	0.00
Reithrodontomys_montanus	2.38	2.97	0.53	0.00
Reithrodontomys_raviventris	35.72	33.42	-0.43	0.01
Reithrodontomys_rodriguezi	9.96	13.88	0.67	0.00
Reithrodontomys_spectabilis	5.36	6.36	0.34	0.03
Reithrodontomys_sumichrasti	3	4.95	0.70	0.00
Reithrodontomys_tenuirostris	3.07	7.93	0.85	0.00
Reithrodontomys_zacatecae	1.14	1.74	0.60	0.00
Rhabdomys_pumilio	0.42	0.67	0.68	0.00
Rhagomys_longilingua	0.01	0.04	0.49	0.00
Rhagomys_rufescens	5.89	9.17	0.74	0.00
Rheithrosciurus_macrotis	0.24	0.57	0.71	0.00
Rheomys_mexicanus	4.77	5.73	0.17	0.29
Rheomys_raptor	2.69	4.24	0.59	0.00
Rheomys_thomasi	1.67	4.02	0.69	0.00
Rheomys_underwoodi	1.8	2.81	0.54	0.00
Rhinoceros_sondaicus	0	0.01	0.06	0.76
Rhinoceros_unicornis	0.12	0.42	0.49	0.00
Rhinolophus_acuminatus	1.73	4.55	0.86	0.00
Rhinolophus_affinis	0.92	2.69	0.83	0.00
Rhinolophus_alcyone	0.19	0.29	0.53	0.00
Rhinolophus_arcuatus	0.76	1.54	0.63	0.00
Rhinolophus_beddomei	3.02	6.36	0.76	0.00
Rhinolophus_blasii	1.55	3.05	0.81	0.00
Rhinolophus_bocharicus	1.43	1.5	0.33	0.04
Rhinolophus_borneensis	0.24	0.6	0.72	0.00
Rhinolophus_canuti	4.01	8.41	0.78	0.00
Rhinolophus_capensis	0.88	1.23	0.61	0.00
Rhinolophus_celebensis	2.3	4.69	0.70	0.00
Rhinolophus_clivosus	0.94	1.74	0.76	0.00
Rhinolophus_coelophyllus	1.63	4.02	0.76	0.00
Rhinolophus_cognatus	0.97	1.84	0.63	0.00

Rhinolophus_creaghi	0.58	1.38	0.70	0.00
Rhinolophus_darlingi	0.78	1.25	0.69	0.00
Rhinolophus_deckenii	0.2	0.35	0.70	0.00
Rhinolophus_denti	0.05	0.11	0.55	0.00
Rhinolophus_eloquens	0.12	0.26	0.68	0.00
Rhinolophus_euryale	4.63	7.91	0.85	0.00
Rhinolophus_euryotis	0.09	0.23	0.58	0.00
Rhinolophus_ferrumequinum	3.2	5.51	0.86	0.00
Rhinolophus_formosae	15.59	22.14	0.82	0.00
Rhinolophus_fumigatus	0.06	0.13	0.65	0.00
Rhinolophus_guineensis	0.03	0.04	0.16	0.32
Rhinolophus_hildebrandti	0.11	0.21	0.61	0.00
Rhinolophus_hilli	0	0	-0.12	0.55
Rhinolophus_hillorum	0.18	0.18	-0.12	0.45
Rhinolophus_hipposideros	4	6.72	0.87	0.00
Rhinolophus_inops	1.49	2.84	0.62	0.00
Rhinolophus_landeri	0.17	0.26	0.62	0.00
Rhinolophus_lepidus	1.78	4.04	0.73	0.00
Rhinolophus_luctus	0.84	2.33	0.81	0.00
Rhinolophus_maclaudi	0.17	0.18	0.10	0.57
Rhinolophus_macrotis	0.49	1.77	0.81	0.00
Rhinolophus_madurensis	2.57	8.39	0.71	0.00
Rhinolophus_malayanus	0.71	2.29	0.81	0.00
Rhinolophus_marshalli	0.51	1.85	0.76	0.00
Rhinolophus_megaphyllus	0.63	0.87	0.62	0.00
Rhinolophus_mehelyi	2.85	5.49	0.83	0.00
Rhinolophus_paradoxolophus	0.47	1.79	0.78	0.00
Rhinolophus_pearsonii	0.89	2.81	0.84	0.00
Rhinolophus_philippinensis	0.2	0.4	0.56	0.00
Rhinolophus_pusillus	1.96	3.63	0.83	0.00
Rhinolophus_rex	0.35	1.51	0.71	0.00
Rhinolophus_robinsoni	1.8	6.08	0.85	0.00
Rhinolophus_rouxii	2.45	5.86	0.82	0.00
Rhinolophus_rufus	1.07	2.15	0.56	0.00
Rhinolophus_ruwenzorii	0.1	0.18	0.50	0.00
Rhinolophus_sedulus	0.5	1.41	0.83	0.00
Rhinolophus_shameli	0.57	1.74	0.70	0.00
Rhinolophus_shortridgei	0.46	1.78	0.80	0.00
Rhinolophus_siamensis	0.08	0.48	0.78	0.00
Rhinolophus_simulator	0.36	0.68	0.70	0.00
Rhinolophus_sinicus	1.14	3.66	0.85	0.00
Rhinolophus_stheno	1.09	2.94	0.80	0.00
Rhinolophus_subbadius	0.68	1.38	0.62	0.00
Rhinolophus_swinnyi	0.32	0.53	0.60	0.00
Rhinolophus_thomasi	0.53	1.8	0.80	0.00
Rhinolophus_trifoliatus	0.83	2.04	0.79	0.00

Rhinolophus_virgo	0.89	1.79	0.62	0.00
Rhinolophus_yunanensis	0.33	1.02	0.69	0.00
Rhinolophus_ziama	0	0	-0.12	0.55
Rhinonicteris_aurantia	0.04	0.08	0.68	0.00
Rhinophylla_alethina	0.8	1.94	0.71	0.00
Rhinophylla_fischerae	0.16	0.3	0.66	0.00
Rhinophylla_pumilio	0.22	0.47	0.73	0.00
Rhinopithecus_avunculus	0.12	0.66	0.56	0.00
Rhinopithecus_bieti	0.01	0.25	0.78	0.00
Rhinopithecus_brelichi	0	0.1	0.33	0.07
Rhinopithecus_roxellana	0.36	1.75	0.80	0.00
Rhinopithecus_strykeri	0	0	-0.12	0.55
Rhinopoma_hardwickii	0.75	1.5	0.73	0.00
Rhinopoma_microphyllum	1.02	1.88	0.74	0.00
Rhinopoma_muscatellum	3.11	5.89	0.85	0.00
Rhinosciurus_laticaudatus	0.97	2.41	0.77	0.00
Rhipidomys_austrinus	0.24	0.46	0.64	0.00
Rhipidomys_couesi	4	6.12	0.74	0.00
Rhipidomys_emiliae	0.15	0.43	0.73	0.00
Rhipidomys_fulviventer	3.55	4.74	0.61	0.00
Rhipidomys_gardneri	0.01	0.04	0.68	0.00
Rhipidomys_latimanus	2.12	3.18	0.56	0.00
Rhipidomys_leucodactylus	0.06	0.16	0.75	0.00
Rhipidomys_macconnelli	0	0.01	0.49	0.00
Rhipidomys_macrurus	0.39	0.91	0.77	0.00
Rhipidomys_mastacalis	2.76	5.27	0.76	0.00
Rhipidomys_modicus	0.07	0.26	0.68	0.00
Rhipidomys_nitela	0.04	0.1	0.70	0.00
Rhipidomys_venezuelae	4	5.22	0.58	0.00
Rhipidomys_venustus	5.96	6.82	0.55	0.00
Rhipidomys_wetzeli	0.01	0.03	0.50	0.00
Rhizomys_pruinosus	0.78	2.38	0.83	0.00
Rhizomys_sinensis	0.82	2.75	0.81	0.00
Rhizomys_sumatrensis	0.71	2	0.77	0.00
Rhogeessa_aeneus	0.93	1.69	0.61	0.00
Rhogeessa_alleni	5.46	7.84	0.68	0.00
Rhogeessa_genowaysi	1.55	4.03	0.51	0.00
Rhogeessa_gracilis	1.39	2.16	0.52	0.00
Rhogeessa_io	0.48	0.92	0.71	0.00
Rhogeessa_minutilla	2.25	3.5	0.67	0.00
Rhogeessa_mira	1.31	1.57	0.45	0.00
Rhogeessa_parvula	2.2	3.25	0.58	0.00
Rhogeessa_tumida	1.51	2.9	0.68	0.00
Rhombomys_opimus	0.33	0.66	0.71	0.00
Rhynchocyon_chrysopygus	0	0.01	0.47	0.00
Rhynchocyon_cirnei	0.03	0.06	0.63	0.00

Rhynchocyon_petersi	0.58	0.93	0.71	0.00
Rhynchocyon_udzungwensis	0	0	-0.12	0.55
Rhynchogale_melleri	0.1	0.18	0.46	0.00
Rhyncholestes_raphanurus	0.24	0.85	0.78	0.00
Rhynchomeles_prattorum	0	0	-0.12	0.55
Rhynchomys_isarogensis	0.04	0.8	0.44	0.01
Rhynchomys_soricoides	0.03	0.14	0.36	0.02
Rhynchonycteris_naso	0.41	0.8	0.70	0.00
Romerolagus_diazi	7.98	9.87	0.34	0.03
Rousettus_aegyptiacus	0.87	1.75	0.79	0.00
Rousettus_amplexicaudatus	0.69	1.78	0.78	0.00
Rousettus_bidens	0.13	0.44	0.60	0.00
Rousettus_celebensis	0.21	0.6	0.59	0.00
Rousettus_lanosus	0.24	0.54	0.70	0.00
Rousettus_leschenaultii	1.67	3.8	0.79	0.00
Rousettus_madagascariensis	0.03	0.07	0.60	0.00
Rousettus_obliviosus	0.47	0.74	0.28	0.09
Rousettus_spinalatus	0.47	0.89	0.50	0.00
Rubrisciurus_rubriventer	0.21	0.63	0.61	0.00
Rucervus_duvaucelii	0.98	2.17	0.65	0.00
Rucervus_eldii	0.07	0.23	0.26	0.11
Rungwecebus_kipunji	0	0	-0.12	0.55
Rupicapra_pyrenaica	2.44	3.86	0.59	0.00
Rupicapra_rupicapra	2.62	5.12	0.77	0.00
Rusa_alfredi	0.17	0.48	0.54	0.00
Rusa_marianna	0.35	0.6	0.44	0.01
Rusa_timorensis	0.85	2.2	0.43	0.01
Rusa_unicolor	1.28	3.03	0.79	0.00
Ruwenzorisorex_suncoides	0.01	0.08	0.45	0.00
Saccolaimus_flaviventris	0.16	0.23	0.58	0.00
Saccolaimus_peli	0.36	0.41	0.29	0.07
Saccolaimus_saccolaimus	1.5	3.26	0.77	0.00
Saccopteryx_bilineata	0.61	1.13	0.71	0.00
Saccopteryx_canescens	0.39	0.69	0.70	0.00
Saccopteryx_leptura	0.53	1.03	0.75	0.00
Saccostomus_campestris	0.22	0.37	0.71	0.00
Saccostomus_mearnsi	0.07	0.17	0.58	0.00
Saguinus_bicolor	2.02	3.83	0.82	0.00
Saguinus_fuscicollis	0.03	0.1	0.75	0.00
Saguinus_geoffroyi	0.52	0.96	0.75	0.00
Saguinus_imperator	0.02	0.08	0.76	0.00
Saguinus_inustus	0	0.01	0.63	0.00
Saguinus_labiatus	0.01	0.08	0.76	0.00
Saguinus_leucopus	1.98	2.48	0.33	0.04
Saguinus_martinsi	0.05	0.12	0.50	0.00
Saguinus_melanoleucus	0.01	0.04	0.70	0.00

Saguinus_midas	0.04	0.12	0.71	0.00
Saguinus_mystax	0.01	0.04	0.51	0.00
Saguinus_niger	0.22	0.59	0.74	0.00
Saguinus_nigricollis	0.27	0.4	0.56	0.00
Saguinus_oedipus	1.47	2.05	0.29	0.07
Saguinus_tripartitus	0.06	0.33	0.45	0.00
Saiga_tatarica	0.1	0.24	0.60	0.00
Saimiri_boliviensis	0.08	0.14	0.66	0.00
Saimiri_oerstedii	0.57	2.59	0.65	0.00
Saimiri_sciureus	0.08	0.21	0.75	0.00
Saimiri_ustus	0.04	0.16	0.78	0.00
Saimiri_vanzolinii	0	0	-0.12	0.55
Salanoia_concolor	0	0	0.25	0.12
Salinoctomys_loschalchalerosorum	0	0	-0.12	0.55
Salpingotus_kozlovi	0.13	0.55	0.81	0.00
Santamartamys_rufodorsalis	1.35	3.26	0.24	0.14
Sarcophilus_harrisii	0.4	0.54	0.36	0.02
Sauromys_petrophilus	0.78	1.26	0.74	0.00
Scalopus_aquaticus	8.21	9.71	0.54	0.00
Scapanulus_oweni	0.24	1.15	0.76	0.00
Scapanus_latimanus	6.16	6.36	0.25	0.12
Scapanus_orarius	3.69	3.61	-0.02	0.93
Scapanus_townsendii	6.98	6.97	0.07	0.69
Scapteromys_aquaticus	1.14	2.2	0.76	0.00
Scapteromys_tumidus	0.95	1.76	0.73	0.00
Scaptonyx_fusicaudus	0.28	1.3	0.80	0.00
Sciurocheirus_alleni	3.91	3.33	-0.33	0.04
Sciurocheirus_gabonensis	0.1	0.18	0.58	0.00
Sciurotamias_davidianus	1.24	3.45	0.83	0.00
Sciurotamias_forresti	0.41	1.87	0.85	0.00
Sciurus_aberti	1.53	1.81	0.44	0.01
Sciurus_aestuans	0.55	1.15	0.77	0.00
Sciurus_alleni	2.8	4.35	0.74	0.00
Sciurus_anomalus	2.17	4.35	0.79	0.00
Sciurus_aureogaster	3.09	4.77	0.67	0.00
Sciurus_carolinensis	8.8	10.03	0.43	0.01
Sciurus_colliaei	1.06	1.63	0.54	0.00
Sciurus_deppei	1.38	2.68	0.67	0.00
Sciurus_granatensis	2.24	3.66	0.64	0.00
Sciurus_griseus	4.95	5.05	0.19	0.24
Sciurus_igniventris	0.07	0.14	0.66	0.00
Sciurus_lis	13.19	15.02	0.53	0.00
Sciurus_nayaritensis	0.88	1.37	0.59	0.00
Sciurus_niger	6.88	8.22	0.52	0.00
Sciurus_oculatus	7.27	10.73	0.72	0.00
Sciurus_richmondi	0.04	0.15	0.62	0.00

Sciurus_spadiceus	0.06	0.15	0.76	0.00
Sciurus_stramineus	1.08	2.85	0.76	0.00
Sciurus_variegatoides	1.02	2.48	0.70	0.00
Sciurus_vulgaris	2.19	3.25	0.73	0.00
Sciurus_yucatanensis	0.55	1.15	0.62	0.00
Scleronycteris_ega	0.01	0.02	0.42	0.01
Scolomys_melanops	0.28	0.54	0.70	0.00
Scolomys_ucayalensis	0.01	0.02	0.68	0.00
Scoteanax_rueppellii	1.51	2	0.63	0.00
Scotinomys_teguina	1.38	3.05	0.70	0.00
Scotinomys_xerampelinus	1.36	2.32	0.55	0.00
Scotoecus_hirundo	0.09	0.19	0.71	0.00
Scotoecus_pallidus	4.97	8.37	0.85	0.00
Scotomanes_ornatus	0.8	2.61	0.81	0.00
Scotonycteris_ophiodon	0.27	0.59	0.68	0.00
Scotonycteris_zenkeri	0.54	0.55	-0.12	0.45
Scotophilus_collinus	2.03	3.9	0.70	0.00
Scotophilus_dinganii	0.17	0.32	0.74	0.00
Scotophilus_heathii	1.84	3.97	0.79	0.00
Scotophilus_kuhlii	1.58	3.57	0.78	0.00
Scotophilus_leucogaster	0.06	0.13	0.71	0.00
Scotophilus_marovaza	0.01	0.01	0.14	0.38
Scotophilus_nigrita	0.09	0.23	0.72	0.00
Scotophilus_nux	0.09	0.24	0.70	0.00
Scotophilus_robustus	0.02	0.03	0.49	0.00
Scotophilus_viridis	0.12	0.25	0.70	0.00
Scotorepens_balstoni	0.05	0.08	0.43	0.01
Scotorepens_greyii	0.08	0.14	0.64	0.00
Scotorepens_orion	2.14	2.69	0.64	0.00
Scotorepens_sanborni	0.11	0.21	0.62	0.00
Scotozous_dormeri	2.74	5.35	0.69	0.00
Scutisorex_somereni	0.05	0.11	0.66	0.00
Sekeetamys_calurus	1.09	2.24	0.77	0.00
Semnopithecus_ajax	0.35	1.29	0.67	0.00
Semnopithecus_dussumieri	2.61	5.42	0.57	0.00
Semnopithecus_entellus	1.29	3.12	0.76	0.00
Semnopithecus_hector	0.52	1.51	0.77	0.00
Semnopithecus_hypoleucos	0.55	1.81	0.54	0.00
Semnopithecus_priam	2.9	7.71	0.86	0.00
Semnopithecus_schistaceus	0.25	0.8	0.72	0.00
Setifer_setosus	0.02	0.04	0.57	0.00
Setonix_brachyurus	0.44	0.63	0.39	0.01
Sicista_armenica	0.23	2.62	0.70	0.00
Sicista_betulina	1.96	2.71	0.47	0.00
Sicista_caucasica	0.04	0.98	0.77	0.00
Sicista_concolor	0.28	1.08	0.81	0.00

Sicista_kazbegica	0.26	0.33	0.15	0.35
Sicista_kluchorica	0.2	0.4	0.50	0.00
Sicista_napaea	0.73	1.02	0.36	0.02
Sicista_severtzovi	3.7	4.15	-0.02	0.93
Sicista_strandi	3.13	3.58	0.07	0.69
Sicista_subtilis	1.76	1.93	0.03	0.88
Sicista_tianshanica	0.15	0.44	0.76	0.00
Sigmodon_alleni	1.63	2.41	0.54	0.00
Sigmodon_alstoni	0.71	1.32	0.68	0.00
Sigmodon_arizonae	1.61	2.25	0.62	0.00
Sigmodon_fulviventer	1.43	2.25	0.61	0.00
Sigmodon_hirsutus	1.88	3.28	0.69	0.00
Sigmodon_hispidus	5.51	6.89	0.54	0.00
Sigmodon_inopinatus	0.84	3.64	0.84	0.00
Sigmodon_leucotis	4.29	6.21	0.66	0.00
Sigmodon_mascotensis	2.05	3.16	0.57	0.00
Sigmodon_ochrognathus	0.93	1.4	0.65	0.00
Sigmodon_peruanus	1.83	4.29	0.77	0.00
Sigmodon_planifrons	0.1	0.52	0.58	0.00
Sigmodon_toltecus	1.67	2.75	0.66	0.00
Sigmodontomys_alfari	0.83	1.44	0.56	0.00
Simias_concolor	0	0	-0.16	0.33
Sminthopsis_aitkeni	0	0	-0.12	0.55
Sminthopsis_bindi	0.05	0.07	0.10	0.53
Sminthopsis_butleri	0	0.02	0.34	0.03
Sminthopsis_crassicaudata	0.06	0.09	0.45	0.00
Sminthopsis_dolichura	0.06	0.08	0.29	0.07
Sminthopsis_douglasi	0	0.01	0.32	0.04
Sminthopsis_gilberti	0.08	0.11	0.29	0.07
Sminthopsis_granulipes	0.08	0.16	0.56	0.00
Sminthopsis_griseoventer	0.76	1.08	0.67	0.00
Sminthopsis_hirtipes	0.01	0.02	0.13	0.41
Sminthopsis_leucopus	0.87	1.23	0.55	0.00
Sminthopsis_longicaudata	0.04	0.07	0.48	0.00
Sminthopsis_macroura	0.02	0.03	0.53	0.00
Sminthopsis_murina	0.4	0.6	0.59	0.00
Sminthopsis_ooldea	0.01	0.03	0.60	0.00
Sminthopsis_psammophila	0.03	0.18	0.62	0.00
Sminthopsis_virginiae	0.09	0.15	0.54	0.00
Sminthopsis_youngsoni	0.02	0.03	0.57	0.00
Smutsia_gigantea	0.09	0.2	0.72	0.00
Smutsia_temminckii	0.08	0.18	0.69	0.00
Solenodon_cubanus	0.13	0.36	0.31	0.05
Solenodon_paradoxus	1.56	3.03	0.44	0.01
Solisorex_pearsoni	3.63	7.85	0.71	0.00
Solomys_ponceleti	0.01	0.02	0.25	0.12

Solomys_salebrosus	0.01	0.02	0.25	0.12
Solomys_sapientis	0	0	-0.12	0.55
Sooretamys_angouya	1.72	3.52	0.81	0.00
Sorex_alpinus	5.28	9.17	0.71	0.00
Sorex_araneus	2.7	3.67	0.57	0.00
Sorex_arcticus	1.4	1.53	0.10	0.57
Sorex_arizonae	1.51	1.79	0.58	0.00
Sorex_asper	0.45	0.82	0.55	0.00
Sorex_bairdi	3.24	3.42	0.21	0.19
Sorex_bedfordiae	0.25	1.29	0.86	0.00
Sorex_bendirii	4.67	4.62	-0.01	0.98
Sorex_buchariensis	0.23	0.2	0.12	0.45
Sorex_caecutiens	0.87	1.14	0.50	0.00
Sorex_camtschatica	0.08	0.09	-0.02	0.93
Sorex_cinereus	2.35	2.51	0.13	0.41
Sorex_coronatus	10.06	14.2	0.82	0.00
Sorex_cylindricauda	0.16	0.95	0.78	0.00
Sorex_daphaenodon	0.46	0.68	0.54	0.00
Sorex_dispar	8.3	8.71	0.09	0.61
Sorex_emarginatus	0.82	1.34	0.40	0.01
Sorex_excelsus	0.03	0.39	0.85	0.00
Sorex_fumeus	9.13	9.57	0.11	0.49
Sorex_gracillimus	0.85	1.17	0.55	0.00
Sorex_granarius	8.31	14.86	0.88	0.00
Sorex_haydeni	2.76	3.39	0.24	0.14
Sorex_hosonoi	4.01	3.96	-0.02	0.93
Sorex_hoyi	2.07	2.15	0.03	0.88
Sorex_isodon	0.9	1.16	0.46	0.00
Sorex_jacksoni	0.01	0.02	0.29	0.07
Sorex_longirostris	7.98	9.88	0.52	0.00
Sorex_lyelli	0.65	0.63	-0.22	0.17
Sorex_macrodon	3.88	5.97	0.60	0.00
Sorex_maritimensis	4.8	4.9	-0.02	0.93
Sorex_mediopua	3.39	5.31	0.63	0.00
Sorex_merriami	1.29	1.46	0.25	0.12
Sorex_milleri	5.18	7.33	0.75	0.00
Sorex_minutissimus	0.86	1.1	0.45	0.00
Sorex_minutus	3.13	4.42	0.70	0.00
Sorex_monticolus	0.84	0.88	0.10	0.57
Sorex_nanus	1.22	1.46	0.30	0.06
Sorex_oreopolus	26.05	30.68	0.70	0.00
Sorex_orizabae	10.33	13.59	0.71	0.00
Sorex_ornatus	10.43	10.98	0.38	0.02
Sorex_pacificus	2.75	2.66	-0.02	0.93
Sorex_palustris	2.29	2.35	0.01	0.98
Sorex_planiceps	0.06	0.15	0.46	0.00

Sorex_preblei	1.28	1.35	0.11	0.49
Sorex_pribilofensis	3.1	2.92	-0.11	0.49
Sorex_raddei	1.77	2.7	0.47	0.00
Sorex_roboratus	0.39	0.52	0.53	0.00
Sorex_rohweri	4.61	4.53	-0.03	0.88
Sorex_samniticus	12.89	18.93	0.84	0.00
Sorex_satunini	2.03	3.37	0.52	0.00
Sorex_saussurei	6.06	8.61	0.74	0.00
Sorex_sclateri	0.55	2.19	0.46	0.00
Sorex_shinto	5.13	5.36	0.02	0.93
Sorex_sonomae	3.4	3.29	-0.11	0.49
Sorex_stizodon	48.65	52.31	0.39	0.01
Sorex_tenellus	0.32	0.38	0.27	0.10
Sorex_trowbridgii	4.51	4.43	-0.01	0.98
Sorex_tundrensis	0.53	0.77	0.67	0.00
Sorex_ugyunak	0.03	0.06	0.39	0.01
Sorex_unguiculatus	0.86	1.27	0.52	0.00
Sorex_vagrans	2.36	2.36	0.00	1.00
Sorex_ventralis	8.26	10.65	0.62	0.00
Sorex_veraecrucis	3.61	5.38	0.66	0.00
Sorex_veraepacis	1.18	2.84	0.64	0.00
Sorex_volnuchini	2.06	3.15	0.48	0.00
Sorex_yukonicus	0.04	0.03	-0.22	0.17
Soricomys_kalinga	0	0	-0.12	0.55
Soricomys_musseri	0	0.11	0.59	0.00
Soriculus_nigrescens	0.07	0.33	0.75	0.00
Spalacopus_cyanus	1.91	4.31	0.85	0.00
Speothos_venaticus	0.51	0.98	0.75	0.00
Spermophilopsis_leptodactylus	0.58	0.84	0.53	0.00
Spermophilus_adocetus	14.02	26	0.93	0.00
Spermophilus_alashanicus	0.77	2.89	0.87	0.00
Spermophilus_annulatus	1.81	2.54	0.52	0.00
Spermophilus_armatus	2.13	2.29	0.11	0.49
Spermophilus_atricapillus	0	0	0.40	0.02
Spermophilus_beecheyi	5.84	6.12	0.30	0.06
Spermophilus_beldingi	0.96	0.94	-0.06	0.74
Spermophilus_brevicauda	0.42	1.03	0.74	0.00
Spermophilus_brunneus	0.77	0.54	-0.08	0.65
Spermophilus_canus	1.01	1.08	0.29	0.07
Spermophilus_citellus	4.87	7.52	0.64	0.00
Spermophilus_columbianus	1.16	1.22	0.10	0.53
Spermophilus_dauricus	1.72	3.97	0.85	0.00
Spermophilus_elegans	1.06	1.18	0.14	0.38
Spermophilus_erythrogenys	0.58	0.7	0.18	0.26
Spermophilus_franklinii	5.13	5.73	0.18	0.26
Spermophilus_fulvus	0.68	0.92	0.50	0.00

Spermophilus_lateralis	1.48	1.65	0.31	0.05
Spermophilus_madrensis	0.04	0.23	0.65	0.00
Spermophilus_major	3.06	3.99	0.30	0.06
Spermophilus_mexicanus	3.23	4.58	0.67	0.00
Spermophilus_mohavensis	5.04	5.18	0.14	0.38
Spermophilus_mollis	0.88	0.9	0.12	0.45
Spermophilus_musicus	0.84	0.43	-0.24	0.14
Spermophilus_pallidicauda	0.05	0.22	0.86	0.00
Spermophilus_parryii	0.05	0.07	0.27	0.10
Spermophilus_perotensis	3.71	6.51	0.60	0.00
Spermophilus_pygmaeus	1.12	1.17	0.10	0.53
Spermophilus_ralli	0.13	0.66	0.74	0.00
Spermophilus_relictus	1.34	1.62	0.49	0.00
Spermophilus_richardsonii	3.17	3.33	0.06	0.74
Spermophilus_saturatus	3.39	3.3	-0.08	0.65
Spermophilus_spilosoma	1.35	1.73	0.50	0.00
Spermophilus_suslicus	3.95	3.96	-0.20	0.22
Spermophilus_taurensis	0.35	0.7	0.52	0.00
Spermophilus_tereticaudus	2.68	3.53	0.65	0.00
Spermophilus_townsendii	5.89	5.53	0.09	0.61
Spermophilus_tridecemlineatus	4.43	5.13	0.33	0.04
Spermophilus_undulatus	0.37	0.55	0.45	0.00
Spermophilus_variegatus	1.75	2.44	0.65	0.00
Spermophilus_washingtoni	2.28	2.38	0.17	0.29
Spermophilus_xanthoprymnus	1.21	2.76	0.66	0.00
Sphaerias_blanfordi	0.47	1.46	0.75	0.00
Sphiggurus_insidiosus	1.16	2.44	0.73	0.00
Sphiggurus_melanurus	0.09	0.18	0.73	0.00
Sphiggurus_mexicanus	1.55	2.98	0.67	0.00
Sphiggurus_pruinosus	3.44	4.72	0.62	0.00
Sphiggurus_spinosus	0.45	1.05	0.78	0.00
Sphiggurus_villosus	1.79	3.79	0.81	0.00
Spilocuscus_kraemeri	0.03	0.03	-0.17	0.29
Spilocuscus_maculatus	0.04	0.09	0.53	0.00
Spilocuscus_papuensis	0	0.06	0.50	0.00
Spilocuscus_rufoniger	0.02	0.05	0.64	0.00
Spilocuscus_wilsoni	0.41	0.47	0.21	0.19
Spilogale_angustifrons	2.6	4.32	0.70	0.00
Spilogale_gracilis	1.87	2.19	0.52	0.00
Spilogale_putorius	5.29	6.5	0.54	0.00
Spilogale_pygmaea	1.79	2.66	0.54	0.00
Srilankamys_ohiensis	3.7	7.95	0.72	0.00
Steatomys_bocagei	0	0.05	0.76	0.00
Steatomys_caurinus	0.09	0.2	0.71	0.00
Steatomys_cuppedius	0.09	0.19	0.69	0.00
Steatomys_krebsii	0.79	1.13	0.67	0.00

Steatomys_opimus	0.01	0.01	0.08	0.65
Steatomys_parvus	0.09	0.19	0.72	0.00
Steatomys_pratensis	0.29	0.49	0.70	0.00
Stenocephalemys_albipes	0.05	0.19	0.73	0.00
Stenocephalemys_albocaudata	0.02	0.06	0.57	0.00
Stenocephalemys_griseicauda	0.01	0.07	0.70	0.00
Stenoderma_rufum	30.24	32.17	0.43	0.01
Stochomys_longicaudatus	0.57	0.54	-0.23	0.16
Strigocuscus_celebensis	0.21	0.62	0.61	0.00
Strigocuscus_pelengensis	0.03	0.01	-0.58	0.00
Sturnira_aratathomasi	1.99	3.13	0.59	0.00
Sturnira_bidens	1.19	2.1	0.67	0.00
Sturnira_bogotensis	1.75	3.08	0.68	0.00
Sturnira_erythromos	0.96	1.61	0.65	0.00
Sturnira_lilium	0.62	1.2	0.73	0.00
Sturnira_ludovici	1.79	2.91	0.64	0.00
Sturnira_luisi	0.87	1.95	0.74	0.00
Sturnira_magna	0.13	0.31	0.75	0.00
Sturnira_mordax	2.98	5.25	0.69	0.00
Sturnira_nana	0.08	0.28	0.68	0.00
Sturnira_oporaphilum	0.23	0.58	0.75	0.00
Sturnira_thomasi	13.83	20.18	0.75	0.00
Sturnira_tildae	0.43	0.87	0.76	0.00
Styloctenium_wallacei	0.21	0.63	0.61	0.00
Stylodipus_andrewsi	0.16	0.54	0.79	0.00
Stylodipus_sungorus	0.01	0.15	0.71	0.00
Stylodipus_telum	0.31	0.51	0.53	0.00
Suncus_aequatorius	0	0	-0.12	0.55
Suncus_dayi	0.62	0.72	0.18	0.26
Suncus_etruscus	3.56	6.4	0.85	0.00
Suncus_fellowesgordoni	2.05	5.72	0.70	0.00
Suncus_infinitesimus	1.53	2.45	0.67	0.00
Suncus_lixus	0.26	0.51	0.76	0.00
Suncus_madagascariensis	0.01	0.02	0.52	0.00
Suncus_megalura	0.34	0.43	0.36	0.02
Suncus_mertensi	2.34	6.07	0.46	0.00
Suncus_montanus	3.02	6.59	0.78	0.00
Suncus_murinus	1.47	3.37	0.78	0.00
Suncus_remyi	0	0.01	0.43	0.01
Suncus_stoliczkanus	2.45	4.78	0.62	0.00
Suncus_varilla	0.6	0.94	0.70	0.00
Suncus_zeylanicus	4.68	9.33	0.73	0.00
Sundamys_infraluteus	0.06	0.12	0.50	0.00
Sundamys_maxi	2.86	5.55	0.50	0.00
Sundamys_muelleri	0.74	1.85	0.75	0.00
Sundasciurus_brookei	0.03	0.07	0.43	0.01

Sundasciurus_fraterculus	0	0	-0.15	0.36
Sundasciurus_hippurus	0.68	1.74	0.75	0.00
Sundasciurus_hoogstraali	0	0.13	0.60	0.00
Sundasciurus_jentinki	0.05	0.09	0.38	0.02
Sundasciurus_juvencus	0.08	0.24	0.58	0.00
Sundasciurus_lowii	0.7	1.78	0.75	0.00
Sundasciurus_mindanensis	0.38	0.97	0.46	0.00
Sundasciurus_moellendorffi	0	0	-0.12	0.51
Sundasciurus_philippinensis	0.34	0.89	0.46	0.00
Sundasciurus_samarensis	0.19	0.56	0.43	0.01
Sundasciurus_steerii	0.01	0.26	0.54	0.00
Sundasciurus_tenuis	0.75	1.87	0.77	0.00
Surdisorex_norae	0.28	0.75	0.37	0.02
Surdisorex_polulus	0.01	0	-0.27	0.16
Suricata_suricatta	0.46	0.65	0.67	0.00
Sus_ahoenobarbus	0.05	0.17	0.61	0.00
Sus_barbatus	0.18	0.53	0.61	0.00
Sus_cebifrons	0.02	0.11	0.50	0.00
Sus_celebensis	0.17	0.49	0.60	0.00
Sus_oliveri	0	0.03	0.22	0.21
Sus_philippensis	0.1	0.31	0.38	0.02
Sus_scrofa	2.15	3.89	0.81	0.00
Sus_verrucosus	1.31	3.99	0.56	0.00
Syconycteris_australis	0.21	0.32	0.63	0.00
Syconycteris_carolinae	0.03	0.13	0.46	0.00
Syconycteris_hobbit	0	0.24	0.44	0.01
Sylvicapra_grimmia	0.15	0.28	0.71	0.00
Sylvilagus_aquaticus	6.92	8.75	0.52	0.00
Sylvilagus_audubonii	1.7	2.14	0.55	0.00
Sylvilagus_bachmani	5.63	5.94	0.31	0.05
Sylvilagus_brasiliensis	0.66	1.31	0.75	0.00
Sylvilagus_cunicularius	3.77	5.38	0.65	0.00
Sylvilagus_floridanus	5.64	6.8	0.55	0.00
Sylvilagus_graysoni	0.61	1.39	0.03	0.88
Sylvilagus_insonus	0.08	0.25	0.28	0.09
Sylvilagus_mansuetus	0	0	-0.12	0.55
Sylvilagus_nuttallii	1.14	1.35	0.26	0.11
Sylvilagus_obscurus	5.41	6.57	0.37	0.02
Sylvilagus_palustris	9.27	11.53	0.63	0.00
Sylvilagus_robustus	0.06	0.07	0.10	0.57
Sylvilagus_transitionalis	22.84	22.84	-0.01	0.98
Sylvisorex_camerunensis	0.15	0.2	0.04	0.83
Sylvisorex_granti	0.1	0.32	0.58	0.00
Sylvisorex_howelli	0.08	0.08	-0.15	0.35
Sylvisorex_isabellae	0.14	0	-0.59	0.00
Sylvisorex_johnstoni	0.12	0.23	0.61	0.00

Sylvisorex_lunaris	0.1	0.29	0.54	0.00
Sylvisorex_morio	0.19	0.08	0.25	0.12
Sylvisorex_ollula	0.02	0.05	0.67	0.00
Sylvisorex_vulcanorum	0.12	0.34	0.64	0.00
Symphalangus_syndactylus	0.49	1.33	0.62	0.00
Synaptomys_borealis	0.62	0.62	-0.04	0.83
Synaptomys_cooperi	7.44	7.98	0.17	0.29
Syncerus_caffer	0.18	0.32	0.75	0.00
Syntheosciurus_brochus	6.45	8.18	0.62	0.00
Tachyglossus_aculeatus	0.15	0.21	0.55	0.00
Tadarida_aegyptiaca	1.33	2.53	0.72	0.00
Tadarida_aloysiisabaudiae	0.03	0.12	0.68	0.00
Tadarida_ansorgei	0.08	0.16	0.66	0.00
Tadarida_australis	0.17	0.23	0.56	0.00
Tadarida_bemmeleni	0.12	0.27	0.70	0.00
Tadarida_bivittata	0.1	0.17	0.49	0.00
Tadarida_brachyptera	0.26	0.47	0.71	0.00
Tadarida_brasiliensis	1.95	2.89	0.69	0.00
Tadarida_bregullae	0.03	0.04	0.15	0.35
Tadarida_chapini	0.05	0.1	0.67	0.00
Tadarida_condylura	0.08	0.19	0.75	0.00
Tadarida_congica	0.02	0.05	0.64	0.00
Tadarida_demonstrator	0.06	0.13	0.71	0.00
Tadarida_fulminans	0.22	0.27	0.19	0.24
Tadarida_jobensis	0.05	0.09	0.59	0.00
Tadarida_jobimena	0.01	0.02	0.15	0.35
Tadarida_johorensis	1.51	5.55	0.90	0.00
Tadarida_kuboriensis	0.04	0.09	0.45	0.00
Tadarida_leucostigma	0.01	0.03	0.51	0.00
Tadarida_lobata	0.49	0.76	0.33	0.04
Tadarida_major	0.13	0.29	0.72	0.00
Tadarida_midas	0.25	0.56	0.72	0.00
Tadarida_mops	0.7	1.46	0.57	0.00
Tadarida_nanula	0.11	0.19	0.65	0.00
Tadarida_nigeriae	0.1	0.18	0.64	0.00
Tadarida_niveiventer	0.04	0.09	0.70	0.00
Tadarida_petersoni	0.31	0.65	0.74	0.00
Tadarida_plicata	1.21	2.79	0.80	0.00
Tadarida_pumila	0.18	0.37	0.72	0.00
Tadarida_solomonis	0	0	-0.12	0.55
Tadarida_spurrelli	0.12	0.32	0.70	0.00
Tadarida_teniotis	3.53	5.97	0.82	0.00
Tadarida_thersites	0.27	0.34	0.29	0.07
Tadarida_tomensis	1.04	2.53	0.62	0.00
Taeromys_celebensis	0.19	0.62	0.57	0.00
Tamandua_mexicana	1.47	2.59	0.64	0.00

Tamandua_tetradactyla	0.44	0.91	0.78	0.00
Tamias_alpinus	0.16	0.15	-0.22	0.17
Tamias_amoenus	1.47	1.49	0.05	0.79
Tamias_bulleri	0.19	0.37	0.35	0.03
Tamias_canipes	0.27	0.38	0.30	0.07
Tamias_cinereicollis	0.77	1.07	0.55	0.00
Tamias_dorsalis	3.07	3.06	0.09	0.61
Tamias_durangae	1.24	1.63	0.64	0.00
Tamias_merriami	5.56	5.59	0.07	0.69
Tamias_minimus	1.21	1.28	0.02	0.93
Tamias_obscurus	2.07	1.98	-0.01	0.98
Tamias_ochrogenys	1.64	1.25	-0.52	0.00
Tamias_palmeri	2.56	1.72	-0.35	0.03
Tamias_panamintinus	0.37	0.36	0.03	0.88
Tamias_quadrimaculatus	1.75	1.6	-0.18	0.26
Tamias_quadrivittatus	1.51	1.74	0.31	0.05
Tamias_ruficaudus	1.67	1.85	0.21	0.19
Tamias_rufus	0.69	0.93	0.44	0.01
Tamias_senex	1.14	1.01	-0.28	0.09
Tamias_sibiricus	0.87	1.45	0.78	0.00
Tamias_siskiyou	1.64	1.67	0.06	0.74
Tamias_sonomae	3.3	3.09	-0.27	0.10
Tamias_speciosus	2.78	2.74	0.08	0.65
Tamias_striatus	7.3	8.12	0.30	0.06
Tamias_townsendii	5.41	5.38	0.05	0.79
Tamias_umbrinus	1.04	1.24	0.35	0.03
Tamiasciurus_douglasii	0.7	1.18	0.68	0.00
Tamiasciurus_hudsonicus	2.61	2.8	0.19	0.24
Tamiasciurus_mearnsi	0.06	0	-0.19	0.31
Tamiops_macclellandii	0.76	2.01	0.82	0.00
Tamiops_maritimus	1.31	3.75	0.85	0.00
Tamiops_rodolphii	0.51	2.18	0.81	0.00
Tamiops_swinhoei	0.42	1.42	0.79	0.00
Tapecomys_primus	0.01	0.09	0.62	0.00
Taphozous_australis	0.93	1.47	0.60	0.00
Taphozous_georgianus	0.04	0.08	0.61	0.00
Taphozous_hildegardeae	0.47	0.82	0.66	0.00
Taphozous_hilli	0.01	0.02	0.21	0.19
Taphozous_kapalgensis	0.11	0.17	0.61	0.00
Taphozous_longimanus	1.56	3.38	0.74	0.00
Taphozous_mauritianus	0.19	0.32	0.73	0.00
Taphozous_melanopogon	1.28	3.03	0.79	0.00
Taphozous_nudiventris	2.47	4.52	0.68	0.00
Taphozous_perforatus	1.2	2.29	0.76	0.00
Taphozous_theobaldi	0.86	2.66	0.85	0.00
Taphozous_troughtoni	0.11	0.21	0.61	0.00

Tapirus_bairdii	1	2.15	0.66	0.00
Tapirus_indicus	0.1	0.56	0.71	0.00
Tapirus_pinchaque	0.06	0.26	0.70	0.00
Tapirus_terrestris	0.49	0.98	0.75	0.00
Tarsipes_rostratus	0.5	0.72	0.64	0.00
Tarsius_bancanus	0.35	0.74	0.70	0.00
Tarsius_dentatus	0.02	0.13	0.65	0.00
Tarsius_pelengensis	0	0.01	0.26	0.13
Tarsius_sangirensis	0.38	0.76	0.26	0.11
Tarsius_syrichta	0.34	0.89	0.46	0.00
Tarsius_tarsier	0.25	0.74	0.58	0.00
Tarsius_tumpara	1.52	1.99	-0.02	0.93
Tarsomys_apoensis	0	0.12	0.27	0.10
Tarsomys_echinatus	0.02	0.36	0.60	0.00
Tatera_indica	2.28	4.46	0.78	0.00
Taterillus_arenarius	0.01	0.04	0.66	0.00
Taterillus_congicus	0.01	0.01	0.43	0.01
Taterillus_emini	0.04	0.1	0.70	0.00
Taterillus_gracilis	0.12	0.27	0.71	0.00
Taterillus_lacustris	0.01	0.03	0.48	0.00
Taterillus_petteri	0	0.02	0.66	0.00
Taterillus_pygargus	0.09	0.21	0.73	0.00
Taterillus_tranieri	0	0.01	0.55	0.00
Taxidea_taxus	3.07	3.58	0.38	0.02
Tayassu_pecari	0.47	0.95	0.76	0.00
Tenrec_ecaudatus	0.02	0.04	0.57	0.00
Tetracerus_quadricornis	2.26	4.79	0.66	0.00
Thallomys_loringi	0.17	0.42	0.55	0.00
Thallomys_nigricauda	0.04	0.12	0.71	0.00
Thallomys_paedulcus	0.15	0.31	0.73	0.00
Thalpomys_cerradensis	0.3	0.68	0.76	0.00
Thalpomys_lasiotis	0.36	0.88	0.77	0.00
Thamnomys_kempi	0.39	1.08	0.46	0.00
Thamnomys_venustus	0.15	0.42	0.59	0.00
Thaptomys_nigrita	1.91	3.84	0.81	0.00
Theropithecus_gelada	0.13	0.38	0.74	0.00
Thomasomys_apeco	0	0	0.00	1.00
Thomasomys_aureus	0.53	1.22	0.71	0.00
Thomasomys_baeops	1.45	3.57	0.82	0.00
Thomasomys_caudivarius	0.82	2.78	0.80	0.00
Thomasomys_cinereiventer	2.18	2.83	0.46	0.00
Thomasomys_cinereus	0.13	0.76	0.76	0.00
Thomasomys_cinnameus	1.64	4.61	0.85	0.00
Thomasomys_daphne	0.26	0.47	0.68	0.00
Thomasomys_eleusis	0.07	0.21	0.52	0.00
Thomasomys_erro	1.7	4.73	0.84	0.00

Thomasomys_gracilis	0.03	0.09	0.51	0.00
Thomasomys_hylophilus	0.92	1.2	0.25	0.12
Thomasomys_incanus	0.08	0.22	0.58	0.00
Thomasomys_ischyrus	0.1	0.25	0.60	0.00
Thomasomys_kalinowskii	0.13	0.38	0.70	0.00
Thomasomys_ladewi	0.03	0.08	0.23	0.16
Thomasomys_laniger	1.94	2.75	0.50	0.00
Thomasomys_macrotis	0	0	-0.01	1.00
Thomasomys_monochromos	0.01	0.02	0.18	0.29
Thomasomys_niveipes	4.09	5.99	0.61	0.00
Thomasomys_notatus	0.15	0.37	0.61	0.00
Thomasomys_onkiro	0	0	-0.12	0.55
Thomasomys_oreas	0.01	0.05	0.40	0.01
Thomasomys_paramorum	1.02	3.23	0.81	0.00
Thomasomys_pyrrhonotus	0.31	1.2	0.70	0.00
Thomasomys_rhoadsi	1.72	4	0.85	0.00
Thomasomys_silvestris	3.51	7.5	0.86	0.00
Thomasomys_taczanowskii	0.07	0.46	0.68	0.00
Thomasomys_ucucha	0.51	2	0.53	0.00
Thomasomys_vestitus	2.51	3.26	0.59	0.00
Thoopterus_nigrescens	0.2	0.59	0.59	0.00
Thrichomys_apereoides	0.51	1.21	0.72	0.00
Thrichomys_inermis	0.29	0.72	0.70	0.00
Thrichomys_pachyurus	0.19	0.37	0.70	0.00
Thryonomys_gregorianus	0.12	0.21	0.48	0.00
Thryonomys_swinderianus	0.28	0.44	0.68	0.00
Thylamys_cinderella	1.06	2.22	0.72	0.00
Thylamys_citellus	0.6	1.51	0.70	0.00
Thylamys_elegans	1.29	3.17	0.80	0.00
Thylamys_fenestrae	0.29	0.63	0.72	0.00
Thylamys_karimii	0.13	0.35	0.76	0.00
Thylamys_macrurus	0.39	1.13	0.71	0.00
Thylamys_pallidior	0.29	0.66	0.77	0.00
Thylamys_pulchellus	0.27	0.66	0.75	0.00
Thylamys_pusillus	0.18	0.33	0.73	0.00
Thylamys_sponsorius	0.59	1.35	0.76	0.00
Thylamys_velutinus	1.25	2.62	0.79	0.00
Thylogale_billardierii	0.39	0.52	0.36	0.02
Thylogale_browni	0.05	0.11	0.45	0.00
Thylogale_brunii	0.06	0.1	0.34	0.03
Thylogale_calabyi	0	0	-0.12	0.55
Thylogale_lanatus	0	0	-0.12	0.55
Thylogale_stigmatica	0.95	1.39	0.64	0.00
Thylogale_thetis	0.4	0.59	0.42	0.01
Thyroptera_discifera	0.36	0.6	0.61	0.00
Thyroptera_tricolor	0.51	1	0.74	0.00

Tlacuatzin_canescens	1.56	2.45	0.56	0.00
Tokudaia_muenninki	3.48	2.06	-0.12	0.45
Tokudaia_osimensis	1.8	2.27	0.18	0.26
Tokudaia_tokunoshimensis	1.94	1.88	0.11	0.49
Tolypeutes_matacus	0.19	0.48	0.77	0.00
Tolypeutes_tricinctus	0.61	1.25	0.65	0.00
Tomopeas_ravus	0.79	1.73	0.79	0.00
Tonatia_saurophila	0.44	0.8	0.68	0.00
Toromys_grandis	0.2	0.52	0.82	0.00
Trachops_cirrhosus	0.54	1.06	0.72	0.00
Trachypithecus_auratus	4.99	9.95	0.75	0.00
Trachypithecus_cristatus	0.6	1.42	0.75	0.00
Trachypithecus_delacouri	0.56	3.37	0.78	0.00
Trachypithecus_francoisi	0.37	1.81	0.81	0.00
Trachypithecus_geei	0.4	1.34	0.63	0.00
Trachypithecus_germaini	1.08	2.41	0.80	0.00
Trachypithecus_hatinhensis	0	0.23	0.80	0.00
Trachypithecus_johnii	1.25	3.57	0.69	0.00
Trachypithecus_laotum	0.01	0.12	0.35	0.03
Trachypithecus_obscurus	1.95	5.9	0.90	0.00
Trachypithecus_phayrei	0.37	1.27	0.72	0.00
Trachypithecus_pileatus	0.82	1.5	0.58	0.00
Trachypithecus_poliocephalus	0.55	6.28	0.86	0.00
Trachypithecus_shortridgei	0	0	0.00	1.00
Trachypithecus_vetulus	2.91	6.83	0.78	0.00
Tragelaphus_angasii	0.07	0.21	0.69	0.00
Tragelaphus_buxtoni	0	0	0.20	0.28
Tragelaphus_derbianus	0	0.01	0.49	0.00
Tragelaphus_eurycerus	0.07	0.18	0.65	0.00
Tragelaphus_imberbis	0.01	0.02	0.50	0.00
Tragelaphus_oryx	0.24	0.4	0.70	0.00
Tragelaphus_scriptus	0.21	0.34	0.66	0.00
Tragelaphus_spekii	0.26	0.28	0.03	0.88
Tragelaphus_strepsiceros	0.11	0.23	0.71	0.00
Tragulus_kanchil	0.55	1.67	0.78	0.00
Tragulus_napu	0.66	1.64	0.75	0.00
Tragulus_nigricans	0	0	-0.06	0.76
Transandinomys_bolivaris	0.62	1.33	0.73	0.00
Transandinomys_talamancae	1.74	2.78	0.60	0.00
Tremarctos_ornatus	0.08	0.18	0.53	0.00
Triaenops_auritus	0	0.03	0.48	0.00
Triaenops_furculus	0.02	0.03	0.26	0.11
Triaenops_persicus	0.45	1.07	0.83	0.00
Triaenops_rufus	0.01	0.01	0.30	0.07
Trichosurus_caninus	1.26	1.78	0.62	0.00
Trichosurus_cunninghami	1.26	1.5	0.55	0.00

Trichosurus_vulpecula	0.37	0.52	0.61	0.00
Trichys_fasciculata	0.56	1.28	0.72	0.00
Trinomys_albispinus	1.9	4.01	0.78	0.00
Trinomys_dimidiatus	6.83	10.45	0.74	0.00
Trinomys_eliasi	8.89	16.24	0.79	0.00
Trinomys_gratiosus	2.8	5.69	0.70	0.00
Trinomys_iheringi	2.56	4.84	0.76	0.00
Trinomys_moojeni	0.09	0.6	0.63	0.00
Trinomys_myosuros	0.49	1.11	0.76	0.00
Trinomys_setosus	0.88	2.44	0.67	0.00
Trinomys_yonenagae	0	0.01	0.39	0.02
Trinycteris_nicefori	0.49	0.85	0.63	0.00
Trogopterus_xanthipes	0.83	2.36	0.79	0.00
Tscherskia_triton	2.09	5.14	0.87	0.00
Tupaia_belangeri	0.5	1.7	0.82	0.00
Tupaia_chrysogaster	0.01	0.01	-0.16	0.33
Tupaia_glis	1.33	3.38	0.78	0.00
Tupaia_gracilis	0.14	0.33	0.69	0.00
Tupaia_javanica	1.55	3.43	0.64	0.00
Tupaia_longipes	0.24	0.57	0.71	0.00
Tupaia_minor	0.67	1.79	0.78	0.00
Tupaia_montana	0.03	0.05	0.33	0.04
Tupaia_nicobarica	0.03	0.1	0.44	0.01
Tupaia_palawanensis	0.05	0.23	0.60	0.00
Tupaia_picta	0.49	1.11	0.73	0.00
Tupaia_splendidula	0.21	0.5	0.63	0.00
Tupaia_tana	0.36	0.8	0.59	0.00
Tylomys_bullaris	48.33	51.22	0.47	0.00
Tylomys_mirae	0.25	0.49	0.57	0.00
Tylomys_nudicaudus	1.27	2.7	0.66	0.00
Tylomys_tumbalensis	0.77	4.23	0.68	0.00
Tylomys_watsoni	1.55	3.3	0.71	0.00
Tylonycteris_pachypus	0.89	2.35	0.79	0.00
Tylonycteris_robustula	0.91	2.3	0.78	0.00
Tympanoctomys_barrerae	0.1	0.31	0.51	0.00
Typhlomys_cinereus	0.43	1.71	0.78	0.00
Uranomys_ruddi	0.1	0.23	0.72	0.00
Urocyon_cinereoargenteus	4.93	5.93	0.58	0.00
Urocyon_littoralis	0.87	0.76	-0.21	0.19
Uroderma_bilobatum	0.54	1.04	0.72	0.00
Uroderma_magnirostrum	0.4	0.75	0.70	0.00
Urogale_everetti	0.38	0.95	0.46	0.00
Uromanis_tetradactyla	0.43	0.46	0.08	0.65
Uromys_anak	0.04	0.09	0.34	0.03
Uromys_boeadii	0.41	0.47	0.21	0.19
Uromys_caudimaculatus	0.07	0.12	0.55	0.00

Uromys_emmae	0	0	-0.12	0.55
Uromys_hadrourus	0	0	-0.23	0.19
Uromys_imperator	0.14	0.26	0.30	0.06
Uromys_neobritannicus	0.03	0.06	0.17	0.29
Uromys_porculus	0.14	0.26	0.30	0.06
Uromys_rex	0.14	0.26	0.30	0.06
Uropsilus_gracilis	0.39	1.89	0.84	0.00
Uropsilus_soricipes	0.32	1.6	0.80	0.00
Urotrichus_talpoides	14.21	15.87	0.50	0.00
Ursus_americanus	1.45	1.51	0.07	0.69
Ursus_arctos	0.78	1.11	0.61	0.00
Ursus_maritimus	0.03	0.04	0.47	0.00
Ursus_thibetanus	1.32	2.94	0.82	0.00
Vampyressa_bidens	0.1	0.24	0.75	0.00
Vampyressa_brocki	0.04	0.09	0.69	0.00
Vampyressa_melissa	0.68	1.15	0.64	0.00
Vampyressa_nymphaea	0.73	1.45	0.68	0.00
Vampyressa_thyone	0.65	1.15	0.66	0.00
Vampyrodes_caraccioli	0.35	0.62	0.67	0.00
Vampyrum_spectrum	0.57	1.01	0.69	0.00
Vandeleuria_nilagirica	0.95	3.24	0.51	0.00
Vandeleuria_nolthenii	0.97	3.14	0.41	0.01
Vandeleuria_oleracea	1.77	3.95	0.77	0.00
Varecia_rubra	0	0	-0.12	0.55
Varecia_variegata	0	0	-0.12	0.55
Vernaya_fulva	0.27	1.32	0.85	0.00
Vespadelus_baverstocki	0.03	0.06	0.50	0.00
Vespadelus_caurinus	0.03	0.05	0.49	0.00
Vespadelus_darlingtoni	1.33	1.72	0.63	0.00
Vespadelus_douglasorum	0.01	0.02	0.46	0.00
Vespadelus_finlaysoni	0.02	0.04	0.55	0.00
Vespadelus_pumilus	2.26	2.82	0.62	0.00
Vespadelus_regulus	0.72	0.94	0.59	0.00
Vespadelus_troughtoni	0.33	0.52	0.64	0.00
Vespadelus_vulturnus	0.49	0.64	0.56	0.00
Vespertilio_murinus	1.9	2.81	0.61	0.00
Vespertilio_sinensis	3.61	5.38	0.83	0.00
Vicugna_vicugna	0.09	0.31	0.70	0.00
Viverra_civettina	6.01	10.16	0.77	0.00
Viverra_megaspila	0.51	2.03	0.84	0.00
Viverra_tangalunga	1.04	2.28	0.76	0.00
Viverra_zibetha	0.83	2.62	0.83	0.00
Viverricula_indica	1.65	3.92	0.81	0.00
Voalavo_antsahabensis	0	0	-0.12	0.55
Voalavo_gymnocaudus	0	0	-0.12	0.55
Vombatus_ursinus	0.73	0.99	0.55	0.00

Vormela_peregusna	1.01	1.73	0.70	0.00
Vulpes_bengalensis	2.42	4.96	0.74	0.00
Vulpes_cana	0.81	1.72	0.73	0.00
Vulpes_chama	0.5	0.76	0.69	0.00
Vulpes_corsac	0.47	0.75	0.65	0.00
Vulpes_ferrilata	0.04	0.15	0.77	0.00
Vulpes_macrotis	1.34	1.74	0.58	0.00
Vulpes_pallida	0.04	0.1	0.69	0.00
Vulpes_rueppellii	0.44	0.78	0.70	0.00
Vulpes_velox	1.7	1.93	0.29	0.07
Vulpes_vulpes	1.95	2.85	0.71	0.00
Vulpes_zerda	0.15	0.19	0.50	0.00
Wallabia_bicolor	0.66	0.92	0.64	0.00
Wiedomys_pyrrhorhinos	0.74	1.75	0.72	0.00
Wilfredomys_oenax	0.32	0.68	0.70	0.00
Xenomys_nelsoni	3.76	5.25	0.57	0.00
Xenuromys_barbatus	0.05	0.1	0.55	0.00
Xeromys_myoides	0.85	1.41	0.57	0.00
Xerus_erythropus	0.18	0.26	0.54	0.00
Xerus_inauris	0.23	0.35	0.66	0.00
Xerus_princeps	0.01	0.05	0.64	0.00
Xerus_rutilus	0.04	0.09	0.61	0.00
Zaedyus_pichiy	0.25	0.55	0.73	0.00
Zaglossus_attenboroughi	0	0.07	0.16	0.40
Zaglossus_bartoni	0.06	0.13	0.38	0.02
Zaglossus_bruijnii	0.12	0.19	0.50	0.00
Zapus_hudsonius	3.77	4.15	0.28	0.09
Zapus_princeps	1.65	1.8	0.15	0.35
Zapus_trinotatus	5.49	5.4	-0.04	0.83
Zelotomys_hildegardeae	0.04	0.11	0.70	0.00
Zelotomys_woosnami	0.02	0.05	0.59	0.00
Zenkerella_insignis	0.02	0.05	0.59	0.00
Zygodontomys_brevicauda	0.86	1.44	0.65	0.00
Zygodontomys_brunneus	3.27	5.01	0.55	0.00
Zyzomys_argurus	0.08	0.16	0.63	0.00
Zyzomys_maini	0.04	0.07	0.49	0.00
Zyzomys_palatalis	0	0	-0.23	0.23
Zyzomys_pedunculatus	0.59	0.66	0.26	0.11
Zyzomys_woodwardi	0	0.01	0.15	0.35
